# Clustering of Alzheimer’s and Parkinson’s disease based on genetic burden of shared molecular mechanisms

**DOI:** 10.1038/s41598-020-76200-4

**Published:** 2020-11-05

**Authors:** Mohammad Asif Emon, Ashley Heinson, Ping Wu, Daniel Domingo-Fernández, Meemansa Sood, Henri Vrooman, Jean-Christophe Corvol, Phil Scordis, Martin Hofmann-Apitius, Holger Fröhlich

**Affiliations:** 1 Fraunhofer Institute for Algorithms and Scientific Computing (SCAI), 53754 Sankt Augustin, Germany; 2 UCB Pharma (UCB Celltech Ltd.), 208 Bath Road, Slough, SL1 3WE Berkshire UK; 3 Bonn-Aachen International Center for IT, University of Bonn, Endenicher Allee 19c, 53115 Bonn, Germany; 4 Department of Radiology and Nuclear Medicine, Department of Medical Informatics, Erasmus MC, University Medical Center Rotterdam, PO Box 2040, 3000 CA Rotterdam, The Netherlands; 5 ICM - Hôpital Pitié Salpêtrière, 47, bd de l’hôpital, 75013 Paris, France; 6 UCB Pharma (UCB Biosciences GmbH), Alfred-Nobel-Str. 10, 40789 Monheim, Germany

**Keywords:** Drug development, Genetics research, Translational research, Computational science

## Abstract

One of the visions of precision medicine has been to re-define disease taxonomies based on molecular characteristics rather than on phenotypic evidence. However, achieving this goal is highly challenging, specifically in neurology. Our contribution is a machine-learning based joint molecular subtyping of Alzheimer’s (AD) and Parkinson’s Disease (PD), based on the genetic burden of 15 molecular mechanisms comprising 27 proteins (e.g. APOE) that have been described in both diseases. We demonstrate that our joint AD/PD clustering using a combination of sparse autoencoders and sparse non-negative matrix factorization is reproducible and can be associated with significant differences of AD and PD patient subgroups on a clinical, pathophysiological and molecular level. Hence, clusters are disease-associated. To our knowledge this work is the first demonstration of a mechanism based stratification in the field of neurodegenerative diseases. Overall, we thus see this work as an important step towards a molecular mechanism-based taxonomy of neurological disorders, which could help in developing better targeted therapies in the future by going beyond classical phenotype based disease definitions.

## Introduction

Many neurological disorders are highly multifaceted, heterogeneous and difficult to treat. The high percentages of clinical trial failures in Alzheimer’s Disease (AD) exemplify the unmet clinical need in the field: While Open Targets today lists 140 compounds that have been tested in clinical studies so far^1^, there are currently only 4 approved ones for symptomatic treatment on the market^2^. The majority of clinical trial failures in neurology (like in other disease areas) can be attributed to a lack of efficacy, and one of the contributing factors is the selection of the wrong target population^3^.

Precision medicine brings the hope of disentangling diseases into separate molecular subgroups, which could be therapeutically targeted more specifically, hence increasing the chances of successful treatment. Moreover, these molecular subtypes may be associated with particular mechanisms, which might allow the identification of novel treatment opportunities. The far-reaching vision is an entirely molecular defined taxonomy of neurological disorders, which should be seen in contrast to the traditional and purely phenotypic way, in which neurological diseases have been defined since the nineteenth century^4,5^.

The AETIONOMY project funded within the Innovative Medicines Initiative (IMI) of the European Union has taken a step into this direction (www.aetionomy.eu). While focusing on Alzheimer’s and Parkinson’s Disease (PD) as important examples, the goal of AETIONOMY was to identify and validate molecular characteristics that could help to stratify AD and PD into more homogeneous patient subgroups. Both neurodegenerative diseases share common properties, such as neuroinflammation^6^, aberrant miRNA expression^7^, and protein misfolding^8^. Accordingly, it has been suspected for a long time that, despite largely non-overlapping causal genetic variants in genome-wide association studies (GWAS), similarities may be expected at the functional or molecular mechanism level^9–11^. Hence, some authors have suggested to focus the analysis on functional categories rather than on individual genetic variants^10^.

The existence of commonly impaired biological processes or mechanisms is also potentially attractive from a therapeutic point of view, since it might open the perspective for a more causal disease treatment. However, the question arises, how homogenous AD and PD patient groups might be with respect to those shared mechanisms, i.e. whether there exist subgroups.

In this work, we explored the genetic burden by single nucleotide polymorphisms (SNPs) on genes that in the literature have been described to play a role in both diseases. We found that, based on aggregate SNP burden scores of common molecular mechanisms in AD and PD, unsupervised machine learning methods can identify distinct and reproducible joint subgroups. We show that these clusters can be associated with distinct clinical, pathophysiological and molecular features on a biological-processes and pathway level, and we investigate the potential clinical utility of these differences by prioritizing drug targets for specific patient subgroups. Altogether, this work shows the possibility of effectively using knowledge about disease mechanisms in combination with modern machine learning techniques to unravel molecular subtypes of AD and PD, which may in the future aid the development of better targeted therapies by contributing to a molecular mechanism-based definition of neurodegenerative diseases.

## Results

### Strategy for identifying mechanism based AD/PD subtypes

**Figure 1:**
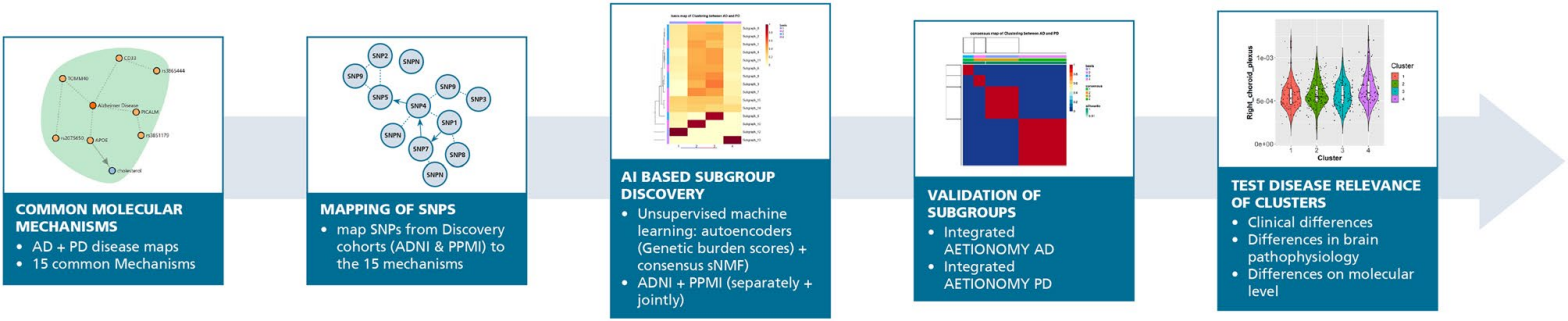
Strategy for identifying AD + PD subtypes.

Before going into more detail, we briefly outline our general approach for identifying subtypes of sporadic AD and PD idiopathic patients (Fig. 1): Following Tan et al.^12^ it is largely driven by the idea of a genetic sub-classification followed by a clinical, imaging based and biological characterization of patients in each cluster to test disease relevance.

**Figure 2:**
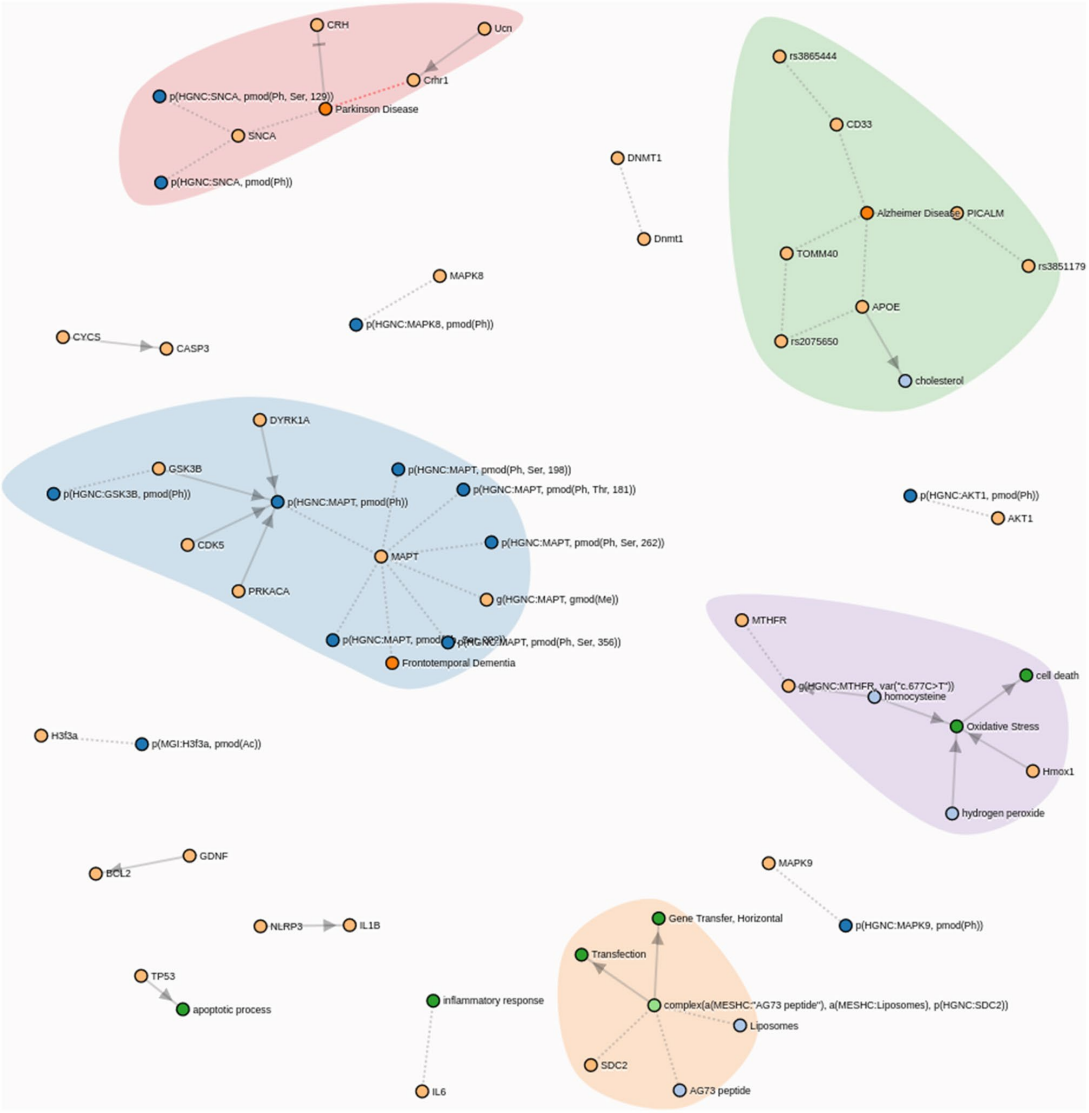
Common AD/PD disease mechanisms, see also https://clus2bio.scai.fraunhofer.de/mechanisms.

**Figure 3:**
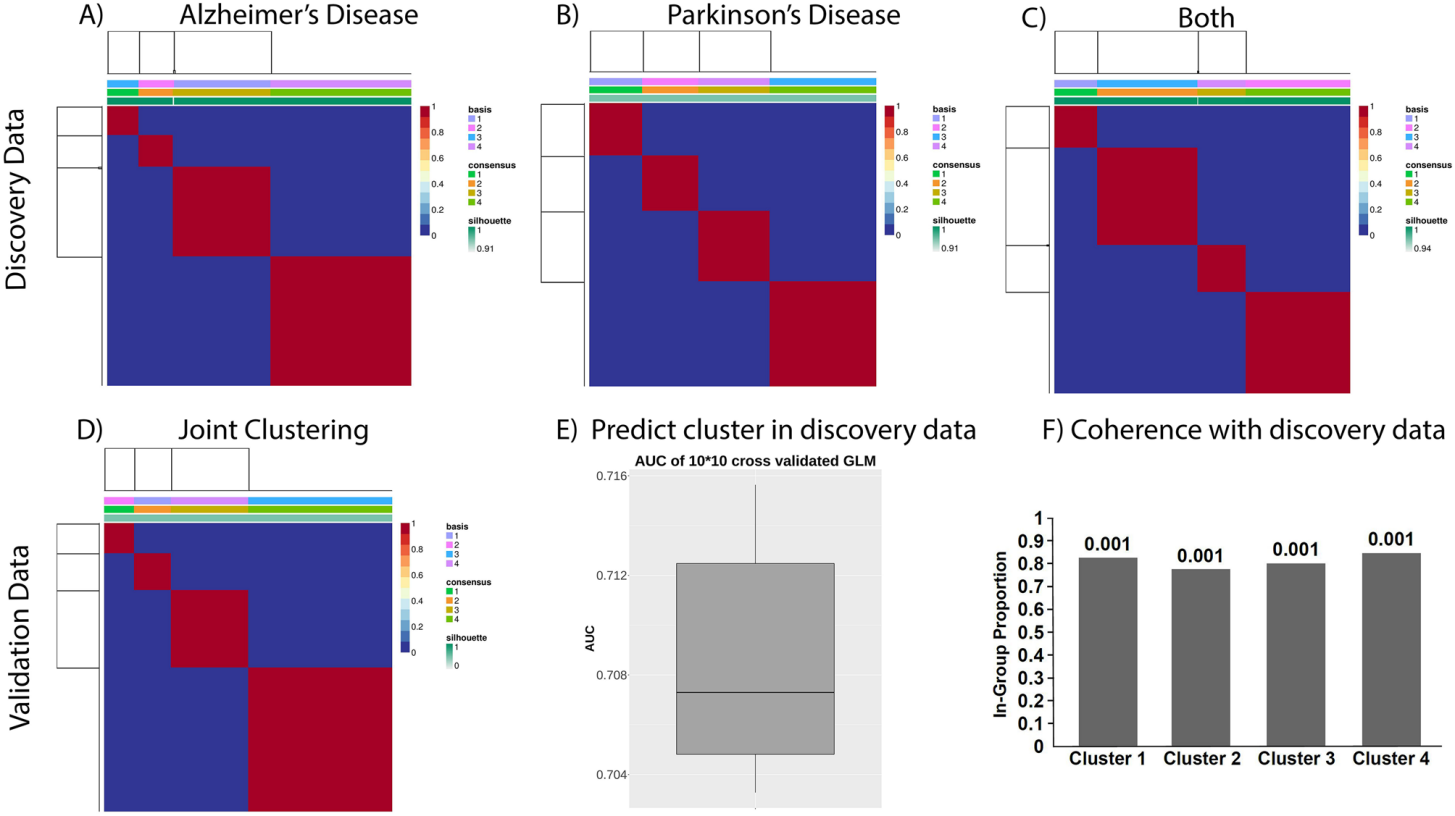
Identification of mechanism-induced subtypes in AD and PD: (**A**) Consensus clustering of ADNI AD patients (consensus matrix). (**B**) Consensus clustering of PPMI PD patients (consensus matrix). (**C**) Consensus clustering of merged ADNI + PPMI (consensus matrix). (**D**) Consensus clustering of merged validation data (integrated AETIONOMY AD + PD, consensus matrix). (**E**) Prediction performance of a classifier that allows assigning each patient in a validation cohort to a cluster in the discovery cohort. (**F**) Coherence of joint AD + PD clustering with validation cohort: Shown is the in-group proportion measure and its p-value according to a permutation test.

Genetic commonalities between AD and PD can only be expected at the biological function level. Hence, the starting point of our work was a comprehensive mapping of the molecular disease landscape of AD and PD based on the scientific literature (see “Methods” section). The result was a set of 15 molecular mechanisms comprising 27 proteins that have been implicated in both diseases (Fig. 2). We mapped 148 SNPs to these genes based on proximity as well as eQTL analysis, see details in Supplements. Using ADNI and PPMI as discovery cohorts (see descriptions in “Methods” section), we calculated for each of the 15 molecular mechanism an aggregate burden score via sparse autoencoders and then used sparse non-negative matrix factorization to identify 4 distinct patient subgroups in AD and PD, see “Methods” section. These subgroups were found independently in both diseases as well as in a merger of ADNI and PPMI patients (Fig. 3A–C).

As a next step, we validated the existence of the identified mixed AD/PD subgroups with the help of disease patients in our integrated AETIONOMY AD and PD cohorts (see description in “Methods” section and Fig. 3D–F). Finally, we tested the disease relevance of the patient subgroups by statistically analyzing the differences of clinical and brain imaging related features as well as transcriptome and methylome profiles in AD and PD patients. Following this high-level overview about our strategy, we will now describe each of the main analysis steps in more detail.

### Mechanism burden scores allow for reproducible subtyping of AD and PD patients

Using the data of 148 SNPs mapping to 15 common AD/PD disease mechanisms in 486 AD and 358 PD patients within our discovery cohorts, we developed an unsupervised machine learning approach to discover subgroups (see details in “Methods” section and Supplementary Text p. 13). This approach consisted of two basic steps: (i) sparse autoencoding of the SNPs mapping to each of the 15 mechanisms, resulting into a profile of genetic burden scores; (ii) consensus sparse non-negative matrix factorization to cluster patients and for identifying most discriminative mechanisms. Our method resulted in 4 subgroups in ADNI, PPMI as well as in a merger of ADNI and PPMI patients that were statistically stable and better discriminated than expected by pure chance (Fig. 3A-C, Tables S2–S4); details are described in the “Methods” section and in the Supplementary Text (p. 28). Interestingly, clusters found in the merged AD/PD cohort were all composed of a mixture of AD and PD patients (Figure S22). They were not identical to the ones identified in each disease individually, but showed a highly significant overlap in both cases (*p* < 1E−16, $\chi 2$-test). That means our clustering suggests the existence of certain commonalities between AD and PD patients on the level of SNP burden on specific mechanisms. We will discuss the question of disease relevance later.

Due to the particular properties of our employed clustering approach, each of the clusters can be linked back to a particular set of disease mechanisms (Figure S21, Table S1 and https://clus2bio.scai.fraunhofer.de/mechanisms for an interactive view): Cluster 1 reflects the genetic burden on AKT1. AKT1 phosphorylation regulates multiple signaling cascades that are of relevance in both AD and PD^13–15^.

Cluster 2 is—among other features—strongly associated with the genetic burden on IL1B, NLRP3, TP53^16–19^. Activation of IL1B by NLRP3 and TP53 play a role in the response of the immune system. Neuroinflammation is a common feature of AD and PD^6^.

One of the features of cluster 3 is the genetic burden on MTHFR, which is implicated in hydrogen peroxide and homocysteine regulation as well as cell death and oxidative stress^20^, Genetic variants may contribute to the risk of PD^21^ and late-onset of AD^22,23^.

Cluster 4 reflects the genetic burden on MAPK9, which is implicated in multiple signaling cascades in both diseases^24,25^.

Again, these are only examples of representative mechanisms for each cluster. A complete overview can be found in Table S1 and under https://clus2bio.scai.fraunhofer.de/mechanisms.

Our next steps particularly focused on the validation of the existence of the joint AD/PD subgroups. For this purpose, we made use of a merger of our integrated AETIONOMY AD and PD validation cohorts, and we asked two essential questions:

1. Does an independent clustering of patients in the validation data re-suggest the same number of clusters?
2. Given the panel of 148 SNPs, can we put patients from our validation cohorts into the same clusters that we had previously identified based on our discovery cohorts, and is the correspondingly induced stratification of patients in the validation cohorts coherent with the clustering of patients in the discovery data?

To answer the first question, we re-ran our developed unsupervised machine learning approach (consisting of sparse autoencoding of each of the 15 molecular mechanisms followed by consensus sparse non-negative matrix factorization), which again supported the existence of 4 clusters composed of mixture of AD and PD patients in the merged validation data (Fig. 3D, Table S5, Figure S23).

To answer question two, we first developed a predictive machine learning algorithm, which allowed us to assign any patient in a validation cohort to one of the established clusters (see “Methods” section). Cross-validation based evaluation of the prediction performance of this classifier was conducted and indicated a decent area under receiver operator characteristic curve (AUC) of ~ 70% that was significantly higher than chance level (Fig. 3E), i.e. clusters were predictable.

Secondly, we measured the coherence of the predicted stratification of patients in our validation cohorts with the one identified in our discovery cohorts. This was done by counting the fraction of patients in the validation cohort whose closest patient in the discovery cohort had the same label, yielding the In-Group Proportion (IGP) measure suggested by Kapp and Tibshirani^26^, see “Methods” section for details. Accordingly, we could verify a high and statistically significant agreement of clusters predicted for patients in the validation data with those in the merged discovery cohort (Fig. 3F). Overall, we thus concluded that our discovered joint stratification of AD and PD patients was reproducible.

### Comparison of clinical outcome measures between clusters

Our next steps focused on the question whether our identified patient clusters were disease associated or just reflecting general genetic differences in the population. For this purpose, we used clinical, imaging, transcriptome and methylome data.

We first investigated differences in clinical outcome measures of AD and PD patients across clusters. This was done separately on the basis of each of the individual study used in this paper (AD: ADNI, ROSMAP; PD: PPMI, AETIONOMY PD, ICEBERG, DIGPD), because available clinical data differs between studies (Tables 1, 2), and differences in inclusion/exclusion criteria may bias a combined analysis: Despite the fact that all patients had a time till initial diagnosis of at most 2 years there were significant differences of baseline UPDRS scores between PD studies (*p* < 1E-9 for MDS-UPDRS I, *p* = 0.02 for MDS-UPDRS II, *p* < 1E-5 for MDS-UPDRS III off treatment score; Kruskal–Wallis test), and in all cases UPDRS total (sum of MDS-UPDRS I + II + III off treatment scores) in PPMI and DIGPD were lower than in AETIONOMY PD and ICEBERG (median UPDRS total in PPMI: 30, DIGPD: 33, AETIONOMY PD: 42, ICEBERG: 47). Similarly, AD cohorts differed significantly by age (*p* < 2E−16, one-way ANOVA), level of education (*p* < 0.01, Kruskal–Wallis test) and MMSE baseline scores (*p* < 1E−10, Kruskal–Wallis test).

**Table 1:** Demographic and clinical variable summary of AD discovery (ADNI) and validation (ROSMAP and IDIBAPS) cohorts.

Cohort	Age	Gender (m/f)	Education	MMSE	CDRSB	ADAS11	ADAS13	RAVLT immediate	RAVLT learning
ADNI	75.55 (9.6)	288/198	16 (5)	24(3)	4.5 (2)	17 (8.33)	27.67 (10)	23 (9)	2 (2)
ROSMAP	87.98 (6.19)	52/142	16 (5)	NA	NA	NA	NA	NA	NA
IDIBAPS	62.42 (9.75)	15/28	12 (9)	18 (7.5)	NA	NA	NA	NA	NA

The data shows only clinically diagnosed sporadic AD cases. The Table shows the median of each variable and the inter-quartile range (IQR) in brackets.

**Table 2:** Demographic and clinical variable summary of PD discovery (PPMI) and validation (AETIONOMY PD, DIGIPD, ICEBERG) cohorts.

Cohort	Age	Gender (m/f)	UPDRS1	UPDRS2	UPDRS3 off	UPDRS3 on	MOCA	HADS anxiety	Schwab-England
PPMI	62.5 (14.14)	238/120	5 (5)	5 (6)	19 (11.75)	NA	28 (3)	NA	NA
AETIONOMY PD	64 (11.25)	59/29	7 (6.75)	7 (8.75)	28 (17)	0 (0)	27 (4)	5 (5)	2 (0)
DIGPD	61 (12)	100/73	7 (6)	5 (5)	21 (11)	0 (0)	NA	6 (4)	2 (1)
ICEBERG	67 (16.5)	30/12	10 (4)	7 (6)	29.5 (11.5)	0 (0)	27 (3)	7 (3)	2 (0)

The data shows only de novo diagnosed idiopathic PD cases. The Table shows the median of each variable and the inter-quartile range (IQR) in brackets.

Based on these observations we focused on a statistical analysis within each of the AD and PD cohorts separately. Notably, IDIBAPS was excluded at this point due to the very small sample size (only 29 cases). Summary statistics of major demographic and clinical baseline variables of all clusters in AD and PD can be found in Tables S7 and S8. Within ADNI we compared multiple cognitive assessment scores (CDRSB, ADAS11, ADAS13, MMSE, MOCA, FAQ, RAVLT, and LDELTOTAL) at the visit of first dementia diagnosis (n = 486 patients) across clusters. The provided cognitive tests cover different aspects, such as global cognitive impairment (ADAS11, ADAS13, MMSE, MOCA), logical memory (LDELTOTAL), verbal episodic memory (RAVLT) and activities of daily living (FAQ). For more detailed information about the composition of individual cognition scores we refer to the literature^27–32^. Notably, cluster labels were based on the clustering of the merged ADNI + PPMI and ROSMAP + AETIONOMY PD + ICEBERG + DIGPD cohorts, respectively. Statistical significances were corrected for multiple confounding factors, such as age, gender, time until diagnosis, ethnicity and the use of L-DOPA (the latter for PD patients). Multiple testing correction was applied via the method by Benjamini and Hochberg^33^. Details about the statistical analysis are described in the “Methods” section part of this paper.

**Figure 4:**
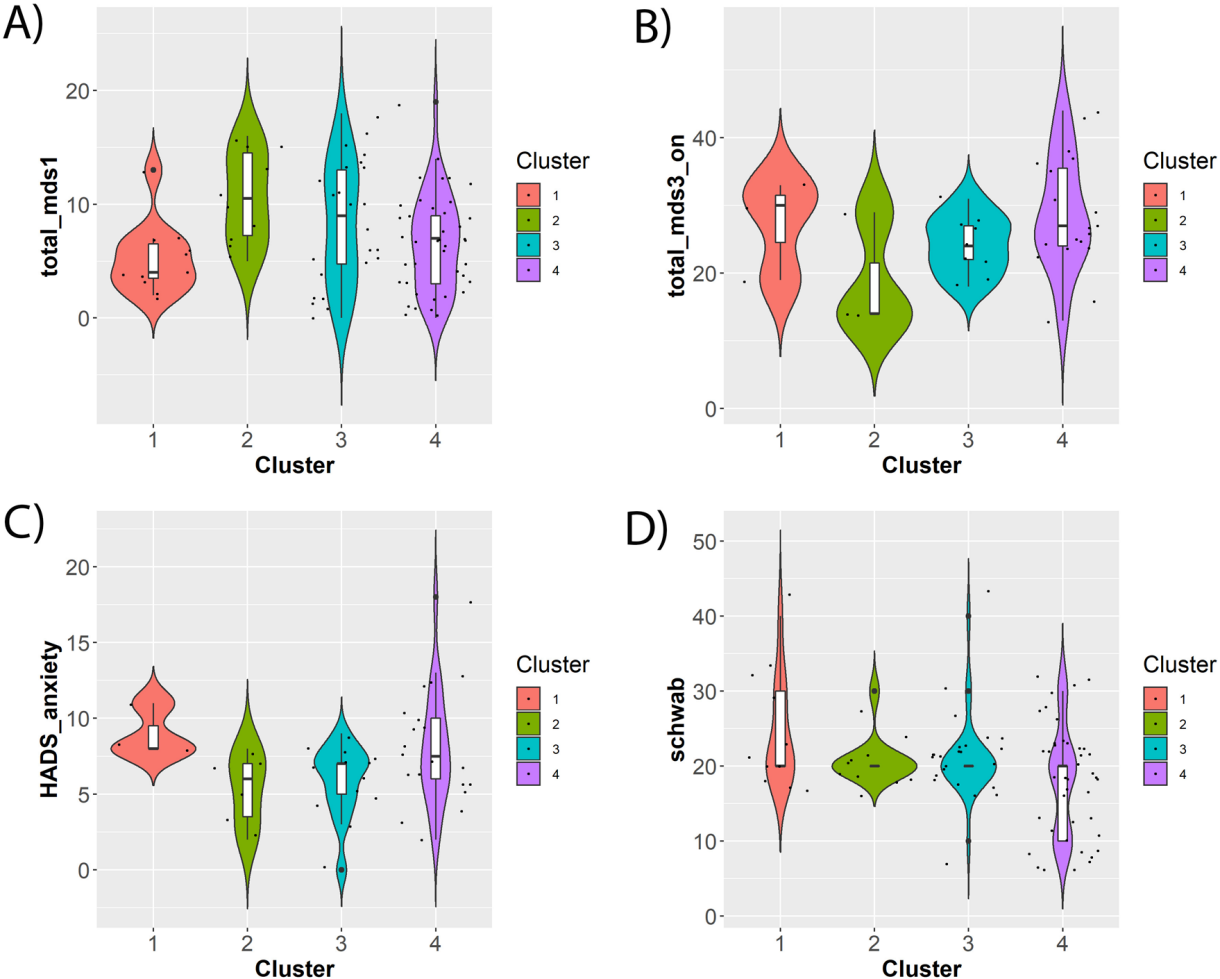
Examples of significant differences between clusters with respect to clinical baseline features in PD patients after correction for confounding effects (see “Methods” section). (**A**) MDS-UPDRS I score (AETIONOMY PD); (**B**) MDS-UPDRS III on treatment score (ICEBERG); (**C**) HADS anxiety score (ICEBERG); (**D**) Schwab-England Scale in % (AETIONOMY PD). The Figures shows statistical distributions as violin plots (i.e. boxplots plus kernel density estimates), and individual data points are shown as superimposed dots.

According to our analysis, no statistically significant differences of cognitive assessment scores could be found between clusters in AD patients at study baseline (although we notably did observe weakly significant results for working memory cognition assessments in ROSMAP patients). However, as indicated in Fig. 4A–D, PD patients in AETIONOMY PD and ICEBERG demonstrated significant pairwise differences between clusters with respect to several clinical baseline scores, namely MDS-UPDRS I (non-motor aspects of daily living; ICEBERG, AETIONOMY PD), HADS anxiety score (ICEBERG), MDS-UPDRS III (motor examination) on treatment scores (ICEBERG) and Schwab-England Scale (difficulties with activities of daily living; AETIONOMY PD). No significant results were found in PPMI and DIGPD.

In addition to this analysis of baseline variables we also conducted an analysis of follow-up longitudinal data, which was available in ADNI (AD) and PPMI (PD) cohorts. This analysis showed significant differences of the progression of MDS-UPDRS III (motor examination) scores across patient subtypes in PPMI. In ADNI we found significant differences in the progression of global cognitive impairment (ADAS11, ADAS13, CDRSB, MMSE) and verbal episodic memory (RAVLT; see Tables S12, S13).

In summary, clusters are associated with significant differences of clinical disease symptoms and symptom progression of AD and PD patients.

### Association with brain imaging derived features in AD and PD

**Figure 5:**
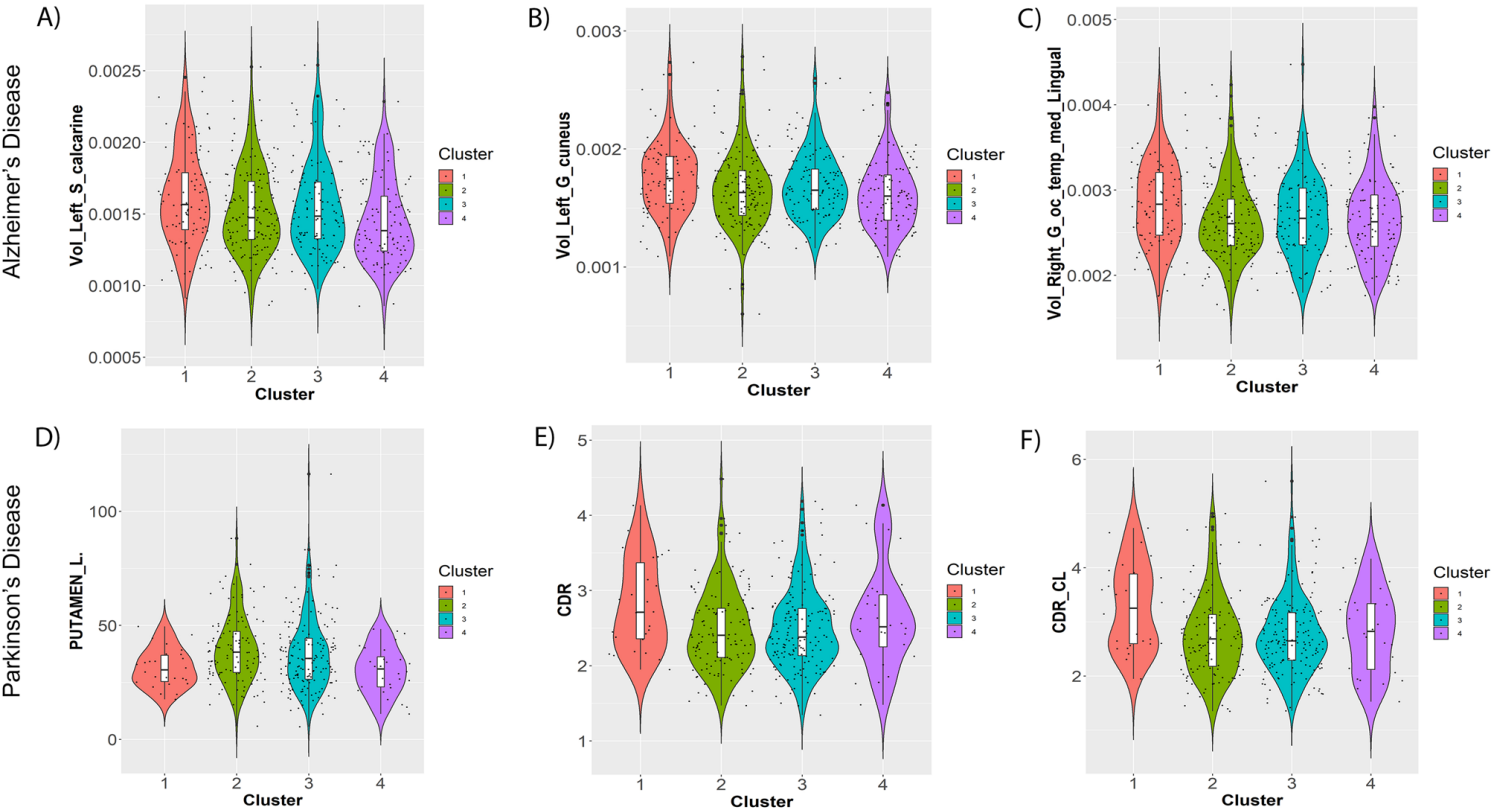
Example of significant differences between clusters with respect to brain imaging derived features at study baseline/time of first disease diagnosis (see “Methods” section). (**A**) left calcarine sulcus in AD patients; (**B**) left cuneus gyrus in AD patients; (**C**) volume of right medial occipitotemporal gyrus in AD patients; (**D**) DaTSCAN left Putamen—ratio to age expected value in healthy controls; (**E**) DaTSCAN Count Density Ratio: Caudate/Putamen; (**F**) DaTSCAN Count Density Ratio (CL): Caudate contralateral/Putamen contralateral. The Figures shows statistical distributions as violin plots (i.e. boxplots plus kernel density estimates), and individual data points are shown as superimposed dots.

In ADNI, AD patients demonstrated highly significant pairwise differences when comparing 193 intracranial volume normalized subcortical brain structures of those patients which had a recent AD diagnosis at study baseline (n = 209) and correcting statistical differences for the confounding effects of age and sex. We found significant differences in several brain regions, such as the calcarine sulcus, the cuneus gyrus and the medial occipitotemporal gyrus (Table S14, Fig. 5A–C).

In PPMI, pairwise differences between the clusters were significant for in presynaptic dopaminergic imaging (DaTSCAN) were identified in caudate and putamen (Table S16, Fig. 5D–F). Also, the dopamine receptor density ratio of caudate versus putamen differed significantly between clusters.

Altogether, we concluded that our genetically derived clusters are associated with significant pathophysiological differences in the brain.

### Association with A-$\beta$, transcriptome and methylome changes

Interestingly, the CSF protein A-$\beta$ showed significant pairwise concentration differences between all clusters in PPMI PD patients (Table S16), but not in ADNI AD subjects. However, there was only weakly significant difference in MOCA cognitive assessment scores across clusters (*p* = 0.1) and no correlation of A-$\beta$ levels with MOCA (*p* = 0.53, Kendall’s tau: 0.03). This is in agreement with Melzer et al.^34^, who reported no association of amyloid-beta deposits with cognitive decline in PD patients.

We further explored changes in transcriptome and methylome of ROSMAP AD patients on the level of Gene Ontology (GO) terms^35^ and KEGG pathways^36^ via Gene Set Enrichment Analysis (GSEA)^37^. This analysis was chosen due to the low sample size, and it can only reveal broad trends in the data, namely statistical enrichment of GO terms and pathway at the beginning or end of a fold change ranked list of genes. We here report findings of GO terms and KEGG pathways that were statistically enriched within one particular patient subtype, but not in others compared to cognitively normal controls. Enrichment maps^38^ were used to provide a condensed view on biological processes and pathways that were particularly altered within one specific cluster (Figure S25–S41). Enrichment maps represent semantic similarities between GO terms (shown as nodes) via edges, and group GO terms together based on the hierarchical relationship between them. More results (including comparisons of one specific cluster to all others) can be found under https://clus2bio.scai.fraunhofer.de).

According to the highly condensed view of enrichment maps, for example, cluster 1 in AD specifically shows changes in the meiotic cycle compared to healthy donors (Figure S26). In fact, aberrant re-entry of neurons into the cell cycle has long been seen as one of the hallmarks of AD^39,40^. Cluster 2 shows transcriptome changes in microtubule-based processes (Figure S27). Indeed, the tau protein, which under healthy conditions stabilizes microtubule, in AD patients aggregates into insoluble filaments in the brain that represent one of the hallmarks of the disease^41^. Specific features of cluster 3 are gene expression changes of processes related to the termination of protein translation (Figure S28). Reduced global translation rates (and RNA levels) have been observed previously in AD patients^42^. Alteration of apoptosis related pathways is one of the features specific for cluster 4 (Figure S29), which is well known in the context of AD^43^. In addition, patients in this cluster show DNA methylation changes in growth factor beta receptors (Figure S35), which has been reported to promote AD pathology^44^. More results can be found in the Supplements.

PPMI transcriptome and methylome data has a larger sample size, but the main limitation is the fact that measurements have been derived from blood and thus only indirectly mirror the pathological processes in the brain. Accordingly, we here again decided to only focus on GSEA results comparing PD patients in each of the clusters against healthy controls (S30–S32; S37–S41). For example, cluster 1 shows specific methylome changes in the JAK-STAT signaling pathway. Inhibition of this pathway has been suggested as a therapy against PD^45^. Cluster 2 shows methylome changes of microtubule cytoskeleton organization. Tau deposition and filament assembly is one of the hallmarks of PD^46^. Assembly of misfolded proteins in PD yields activation of adaptive immune response^47^. According, transcriptional changes can be observed in cluster 2 as well. Cluster 3 demonstrates methylome changes of lipoprotein metabolism, which has recently been found altered in PD^48^. Cluster 4 shows transcriptional changes in protein ubiquitination, which has been suggested to also play a role in idiopathic forms of PD^49,50^. In addition, methylome changes of several metabolic processes were observed, which is in agreement with recent findings that view PD as a disorder of the cell metabolism^51^. Again, more results (including enrichment maps for GO terms) can be found in the Supplements and under https://clus2bio.scai.fraunhofer.de.

Altogether, our examples suggest that—despite the obvious limitations of the employed molecular data—each of the four clusters can be associated with biological processes that are solely enriched in one cluster and that are well known in the context of both diseases. Epigenetic changes were observed to a much higher extent in PD than in AD.

### Molecular differences between clusters can be linked to known disease mechanisms

We next explored GO terms (biological processes) and KEGG pathways that were enriched in the *difference* between one cluster to all others. In other words, we looked into differential expression and differential methylation between cluster 1 and all others, cluster 2 and all others, and so on. For each of these comparisons a larger number of biological processes and pathways could be identified in both AD and PD (Tables S18, S20, S22, S24). In agreement to the findings in the last Section, significant differences between clusters in methylation could only be found in PD patients, but not in AD. Transcriptome differences between clusters were observed in both diseases.

We further explored the link between differences at the transcriptome and methylome level among clusters and known disease mechanisms in AD and PD. More specifically, we mapped our initially identified 15 common AD/PD disease mechanisms to disease specific mechanisms defined in the NeuroMMSig database^52^. That means, each of the common AD/PD mechanisms used in our clustering was identified with a certain NeuroMMSig gene set, if it was contained in that gene set. We found at least one NeuroMMSig gene set for each of the 15 mechanisms. Since each NeuroMMSig gene set equals a subgraph in one of our literature derived AD and PD disease maps (see “Methods” section), we could then systematically conduct graph mining. More specifically, we looked for shortest paths linking NeuroMMSig gene sets with biological processes and pathways identified in our omics data analysis. Shortest path calculations considered the causal direction of edges (marking e.g. a phosphorylation event) whenever possible. Due to the large number of results (over 600), we decided to implement an interactive web application for exploration (https://clus2bio.scai.fraunhofer.de/biomarkers). The web application also provides pointers to the scientific literature supporting each of the edges.

**Figure 6:**
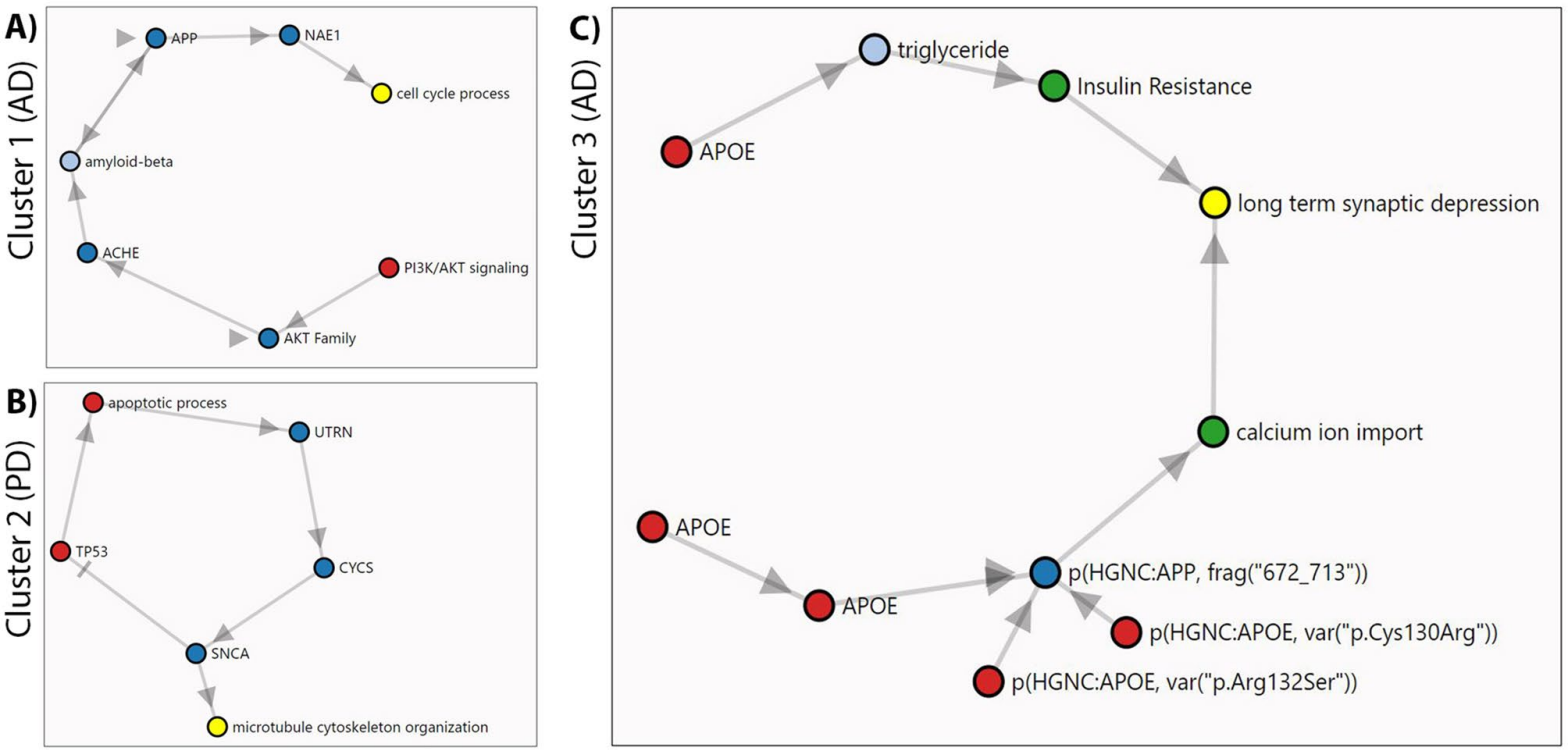
Examples of GO terms (biological processes) found significantly enriched in gene expression and/or methylome changes of one cluster compared to all others (yellow) together with their connections to genes playing a role in common AD/PD disease mechanisms (red): (**A**) Connection between AKT signaling (feature of cluster 1) and cell cycle in AD; (**B**) connection between TP53 (features of cluster 2) and microtubule cytoskeleton organization in PD; (**C**) connection between APOE4 (feature of cluster 3) and long term synaptic depression in AD. Intermediate signaling proteins are shown in blue. For more examples visit https://clus2bio.scai.fraunhofer.de/biomarkers.

In the following, we highlight only selected examples (Fig. 6): As explained previously, cluster 1 is strongly associated with the genetic burden on AKT signaling. At the transcriptional level we observed significant downregulation of genes in the cell cycle process in AD patients (adj. *p* value 0.03). Both can be linked together, as shown in Fig. 6A. AKT signaling influences acetylcholinesterase (AChE), which is thought to play a role in apoptotic processes^53^ and amyloid-beta formation^54^. Amyloid-beta increases NAE1 via APP^55^ and influences the entire cell cycle process^56^.

In cluster 2, for PD patients we observed differential methylation of genes involved in processes related to microtubule cytoskeleton organization (adj. *p* value < 0.001). Cluster 2 is—among others—associated with the genetic burden on TP53. As shown in Fig. 6B there is indeed a causal chain between TP53 and microtubule cytoskeleton organization. Elevated TP53 levels have been found to induce apoptosis and inflammation in PD^57^. Apoptotic processes yield a translocation of UTRN from the cytosol to mitochondria and subsequently increases cytochrome C^58^ and alpha-syn^59^, which itself is involved in microtubule cytoskeleton organization^60^.

In cluster 3, for AD patients we observed significant transcriptional downregulation of genes involved in “long term synaptic depression” (adj. *p* value 0.02). Cluster 3 is at the same time associated to the genetic burden on APOE. The connection between both is highlighted in Fig. 6C. For example, APOE has been suggested to increase insulin resistance^61^, which yields synaptic depression of neurons and thus suggests the perception of AD as a “type 3 diabetes”^62^.

Once again, these are only examples and further results can be explored via our web application.

### Potential implications for drug development

Our previous results indicate that our AD/PD clustering can be associated with molecular and pathophysiological differences between patient subgroups. To better understand the potential utility of these patient subgroups for improving future AD and PD therapy, we conducted a target prioritization of all 27 genes involved into the 15 mechanisms that we had previously used in conjunction with SNP data to identify cluster patients. Target prioritization was done via Open Targets^1^, which uses genetic evidence as well as literature mining to assign a confidence score to each protein as a potential drug target. In addition, tractability by small molecules and antibodies was considered. Figures S41, S42 highlight that in both diseases several potential targets could be identified via Open Targets. In addition, some of these targets could be clearly associated to one specific cluster (Table S1): In AD genes CDK5, GSK3B are strongly associated to cluster 2 (Table S1). APOE, PICALM, TOMM40, MTHFR and CD33 are linked to cluster 3. Further potential targets include SNCA, IL6 and CYCS, which are more strongly associated with clusters 2 and 3 than to the rest.

In PD, only SNCA, MAPT and APOE were identified as potential targets (Figure S42). MAPT is strongly associated with cluster 2 and APOE to cluster 3 (Table S1).

Altogether this analysis shows that our patient subtypes might be used to inform better targeted therapeutic strategies in AD and PD in the future.

## Conclusion

Precision medicine offers the hope of delivering the right treatment to the right patient, based on individual characteristics rather than population averages for these characteristics. Precision medicine is only an emerging reality at this moment, and moving closer to this vision will require non-trivial efforts in data mining and machine learning based on the entirety of available patient data^63^. Specifically, in neurology, this is extremely challenging, because on the one hand diseases are often highly multifaceted and on the other hand deep molecular multi-omics data (as frequently employed in cancer research) are difficult or even impossible to obtain for obvious reasons. Accordingly, in this work we started with an intensive literature mining effort, which mapped out the current mechanistic understanding of AD and PD pathologies and allowed us to identify shared molecular mechanisms. These shared molecular mechanisms were used as a starting point for developing a joint molecular subgrouping of AD and PD. More specifically, we used state-of-the-art unsupervised machine learning techniques to identify four mixed AD + PD patient clusters based on SNP burden scores of common AD/PD mechanisms. Importantly, the resultant disease subtypes manifest as mixtures of different mechanisms rather than being instances of single ones.

We validated the existence of patient clusters based on combined genotypes of 561 patients from AETIONOMY PD, ICEBERG, DIGPD, ROSMAP and IDIBAPS studies. Moreover, we conducted an in-depth analysis of clinical, imaging and molecular differences between patient clusters in both diseases. Our work demonstrated that SNP burden on mechanism level can be used to subdivide AD as well as PD patients jointly, and that clusters are associated with clinical, pathophysiological (specifically visible in brain imaging) and molecular differences between patients. We investigated the potential clinical utility of these differences by prioritizing drug targets for specific patient subgroups.

Overall, one should see our approach as complementary to the multitude of existing work that focuses on separate subgrouping of AD and PD based on polygenic risk scores^64^, CSF, blood and imaging biomarkers^65–67^, or based on clinical outcome measures^68,69^. We see the main distinction of our approach in a better understanding of the stratification potential of common AD and PD disease mechanisms, including the implications for future drug development.

Of course, our work is not without limitations: These can largely be associated to the limited availability of transcriptome and methylome data (with matched SNP genotypes from the same patient) in only two studies (ROSMAP and PPMI) and with relatively low sample sizes in ROSMAP. Moreover, clinical differences between cohorts imposed non-trivial challenges for reaching coherent conclusions regarding the clinical differences between patient subgroups. We thus see a need to more systematically replicate observational clinical studies in the neurology field. At the same time, such studies should preferably be longitudinal and collect multi-omics data from the same patient in a more systematic way than currently done in ROSMAP and PPMI. Such data should then be used to re-validate the findings presented here, specifically in terms of molecular differences between patient subgroups.

Altogether, we see our work as a step towards realizing the far-reaching vision of a completely molecular based definition of human disease, as formulated by Kola and Bell^4^ and Strafella et al.^5^. As pointed out before, we see the potential impact of such an effort in the development of better targeted and thus hopefully more efficacious therapies in AD and PD in the future.

## Materials and methods

### Overview about used data

#### Studies used for discovery

##### ADNI

Data were obtained from the Alzheimer’s Disease Neuroimaging Initiative (ADNI) database (www.adni.loni.usc.edu). The longitudinal observation study includes—among others—486 subjects, which were diagnosed with mild sporadic AD during the study. 206 patients had a recent clinical AD diagnosis at study baseline. Data from ADNI subjects includes SNP based genotype (two different Illumina chip platforms), APOE4 status, CSF biomarkers, volume measurements of seven brain regions as well as different clinical and neuropsychological test results. In addition to the 7 brain volume measurements provided in the original ADNIMERGE dataset we calculated 193 subcortical brain region volumes from raw images, using the parcellation by Destrieux et al.^70^, see details in Supplements. An overview about key demographic and clinical features of this study can be found in Table 1.

##### PPMI

The Parkinson’s Progression Markers Initiative (PPMI) (www.ppmi-info.org/data) consists of multiple cohorts from a network of clinical sites with the aim to identify and verify progression markers in PD. It is a longitudinal observation study with data collected using standardized protocols^71^. PPMI comprises of eight cohorts with different clinical and genetic characteristics. Here we used data of 358 de novo diagnosed idiopathic PD patients and 198 healthy controls. All PD patients were initially untreated and diagnosed with the disease for two years or less. The dataset contains information about patient demographics, patient PD history, DaTSCAN imaging, non-motor symptoms, CSF biomarkers (A-$\beta$, $\alpha$-synuclein, dopamine, phospho-tau, total tau) and UPDRS scores. Genotype was available via whole genome sequencing data. In addition, whole blood transcriptome and methylome data was available for n = 306 and n = 277 of the same patients, respectively. The number of healthy controls with available gene expression and DNA methylation data was n = 151 and n = 112. An overview about key demographic and clinical features of this study can be found in Table 2.

#### Studies used for validation

##### Integrated AETIONOMY AD

Validation data comprised 237 clinically diagnosed sporadic AD cases with available genotype from ROSMAP^72^ (n = 194) and 21 additional cases with available genotype from IDIBAPS taken from the AETIONOMY biomarker verification study^73^. We call the union of these 258 AD patients *integrated AETIONOMY AD* in the following. The data included:

- clinical characteristics: e.g. post-mortem diagnosis, age at death, gender
- genome-wide transcriptome (n = 56 AD cases with jointly available genotype and n = 50 cognitively normal controls) and methylome data (n = 53 AD cases with jointly available genotype and n = 34 cognitively normal controls) from post-mortem brain tissue (ROSMAP)

An overview about key demographic and clinical features of this study can be found in Table 1.

##### Integrated AETIONOMY PD

Validation data comprised idiopathic PD cases with available genotype that were diagnosed with PD for 2 years or less (in agreement to PPMI). 173 out of the 303 cases stem from DIGPD (NCT01564992)^74^, 42 from ICEBERG (NCT02305147) and 88 were taken from study that is henceforth referred to as AETIONOMY PD^75^. We call the union of the 303 idiopathic PD patients *integrated AETIONOMY PD* in the following. The datasets are cross-sectional and include typical clinical outcome variables, such as MDS-UPDRS, Hoehn and Yahr stage, cognitive assessment scores (MMSE, MOCA), Epworth sleepiness scale (ESS), REM sleep behavior disorder (RBD), Hospital anxiety, and depression scale (HADS). An overview about key demographic and clinical features of this study can be found in Table 2.

### Identification of common molecular mechanisms

Common molecular mechanisms between AD and PD were identified with the help of a systematic literature mining approach with post-hoc manual curation. More specifically, the text mining engine SCAIView^76^ was used to construct cause-effect relationships between molecules, pathways, biological processes and imaging features in both, AD and PD, see Domingo-Fernandez et al. and Kodamullil et al. for details^52,77^ for details. After manual curation, two computable disease maps, one for AD and one PD were created. Finally, we have also made them interactively usable via a dedicated web application (https://neurommsig.scai.fraunhofer.de/).

Calculation of the intersection of cause-effect relationships described in the AD and PD disease maps resulted into 27 genes grouped into 15 cause-effect relationship sub-graphs, called *mechanisms* from now on (see Fig. 2 and https://clus2bio.scai.fraunhofer.de/mechanisms for an interactive view). While some of these mechanisms describe only posttranslational modifications of a single protein, others reflect more complex protein–protein interactions and signaling cascades (Table S1). Key proteins described in both diseases include e.g. APOE, TAU, SNCA and TOMM40. These proteins are involved into several known disease relevant processes that we have made computationally accessible via our earlier developed NeuroMMSig database^52^.

We mapped 148 genetic variants (SNPs) measured in ADNI1, ADNI2/GO as well as PPMI to the 27 common AD/PD disease genes via a combination of two strategies: a) proximity (using a 10 kbp window size); and b) eQTL mapping, see details in Supplements on page 2.

### Calculation of SNP burden on molecular mechanism level

SNP data is inherently extremely sparse, i.e. even “common” genetic variants are comparably seldom in the data. This imposes a major challenge for any clustering algorithm, because the distance between two arbitrary SNP profiles based on the usual 0, 1, 2 encoding then becomes almost identical. That means clustering of raw SNP profiles is prone to become statistically unstable and noisy. To account for this fact, we embedded the 148 SNP profiles of AD and PD patients into a lower dimensional latent space while taking into account the grouping of SNPs according to 15 molecular cause-effect relationship subgraphs (aka molecular mechanisms) defined in the last section. We aimed for making this embedding non-linear to capture SNP-SNP interactions. Very recently, autoencoder networks (a specific deep learning technique) have been proposed for that purpose^78,79^. Autoencoders allow for learning a non-linear and low dimensional representation of SNP data for each patient, i.e. in essence a SNP burden score per mechanism (see Supplements for details). Based on the SNP burden scores a grouping of AD and PD patients can be established via clustering. Details will be explained later.

To maximize the chance for a later interpretation of the clustering and to avoid an imbalance due to differences in the number of mapped SNPs, we learned (sparse) autoencoder based SNP burden scores for each of the 15 mechanisms. That means we ended up with a 15 dimensional vector of genetic burden scores for each patient. Each of these 15 scores can be interpreted in terms of the relative contribution of each SNP to the overall score learned by the autoencoder network (Figures S2–S16). Details about the training procedure for our sparse autoencoder networks are described in the Supplementary Material on page 13.

### Unsupervised machine learning for patient (Bi-)clustering

Based on the 15 dimensional SNP burden profile of each patient derived from SNP data we clustered patients. We here relied on sparse non-negative matrix factorization (sNMF). Briefly, sNMF factorizes a patients times mechanisms matrix $X$ into a product of two non-negative matrices $W$ and $H$, where $W$ represents a sparse mapping of mechanisms to clusters and $H$ a soft assignment of patients to clusters^80,81^. That means, for each patient cluster it is possible to identify the most influencing mechanisms (see Supplements for details). Hence, sNMF effectively yields a bi-clustering. The entire (bi-)clustering procedure is in practice is an iterative process that is dependent on the initialization of both matrices and should thus be repeated a number of times (here: 50) to yield a consensus. This consensus was used for further analysis.

The number of clusters $k$ corresponds to the number of columns of matrix $W$ and the number of rows of matrix $H$. We chose $k$ based on inspection of three statistical criteria (proportion of ambiguously clustered pairs, silhouette index, cophenetic correlation) and in comparison to a randomly permuted cluster assignment^82–84^. We then decided for the minimal number of clusters $k$ yielding the most stable clustering solution (lowest proportion of ambiguously clustered pairs) that was at the same time exhibiting a significantly larger silhouette index and cophenetic correlation than expected by chance. Details are explained and shown in Supplements on page 28.

### Validation of patient subtypes via independent studies

**Figure 7:**
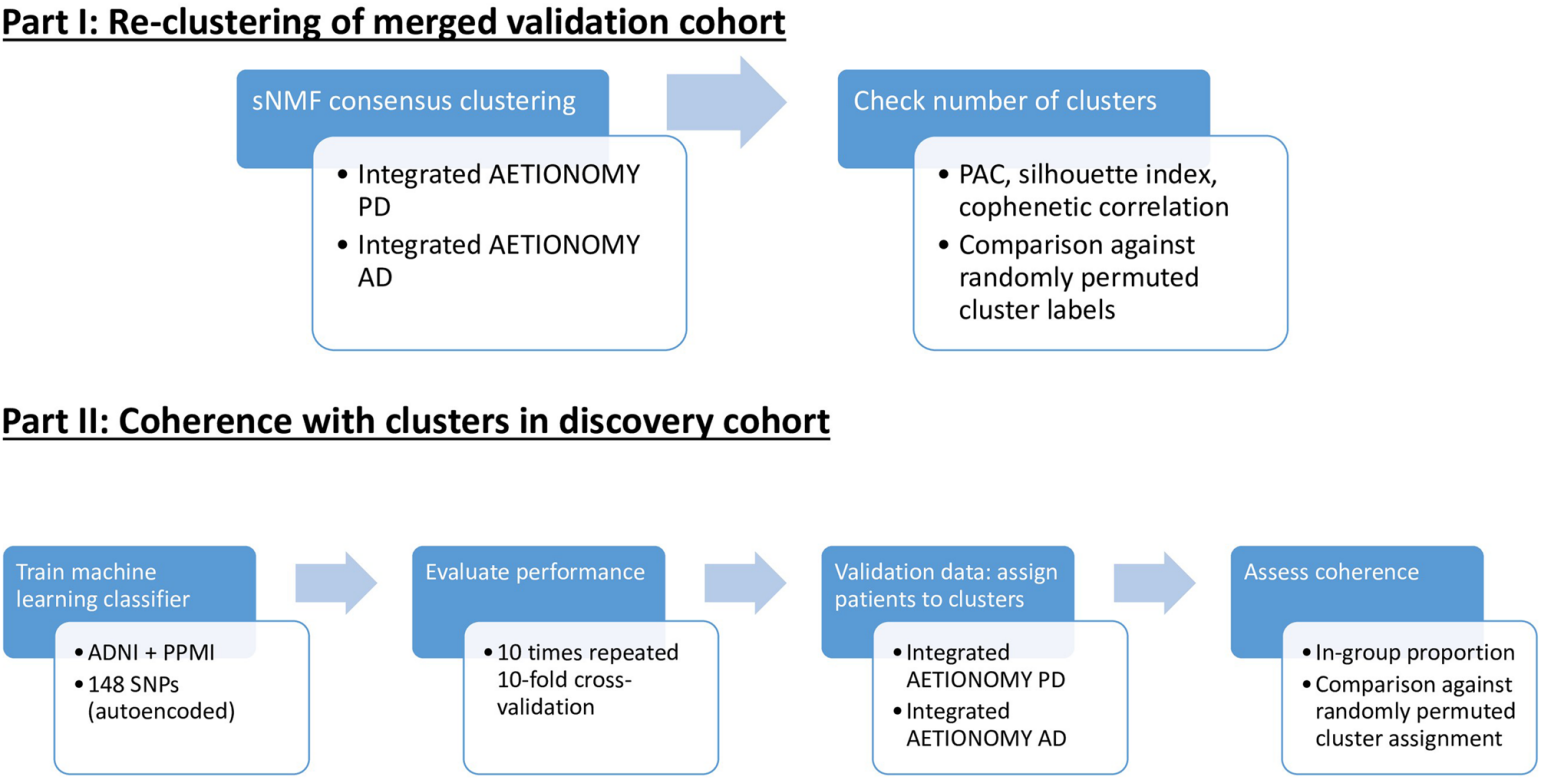
Strategy for validating genetically defined patient clusters.

Figure 7 gives an overview of our overall validation strategy, which consists of two parts: In the first part we re-clustered patients in our merged AD/PD validation cohort using the same workflow that we had established for our discovery cohort, which re-confirmed the possible existence of 4 clusters in AD and PD (Table S5).

In the second part of our validation, we followed the idea of assigning patients in an independent study to the pre-existing clusters discovered in ADNI and PPMI and then measuring the degree of coherence between the cluster assignments and originally discovered groups. For this purpose, we here adopted an approach proposed in Kapp and Tibshirani^26^: Following that approach we first developed a supervised machine learning classifier on the basis of the SNP data of patients in ADNI and PPMI. This allowed us to predict for any patient in an independent validation cohort the membership to a cluster in the discovery cohort based on the 148 SNP panel described above. We used an $l_{1}$ penalized logistic regression (i.e. LASSO) as a classification algorithm, and we evaluated the prediction performance of this classifier via a conventional 10 times repeated tenfold cross-validation procedure. That means we subsequently left out 1/10 of our discovery data for testing the classifier and only trained on the remaining 9/10 of the data. Autoencoder training and $l_{1}$ penalty hyper-parameter optimization was done within the cross-validation loop to prevent overoptimism. The corresponding cross-validated multi-class AUC of 70% is shown in Fig. 3E.

After classifier development, we were able to assign patients from independent studies to the clusters discovered in our discovery cohort (ADNI + PPMI). The in-group proportion measure (IGP) proposed by Kapp and Tibshirani then measured the proportion of patients in the validation study, whose nearest neighbors in the discovery cohort had the same cluster label. An IGP closer to 1 indicates a stronger coherence of the statistical distribution of data in the validation cohort with the clustering of the discovery cohort. An IGP closer to zero indicates disagreement.

To further assess the statistical significance of observed IGP values we performed a permutation test, in which we randomly permuted the cluster assignment of patients and re-calculated the IGP. This was done for 1000 times. None of the randomly permuted cluster assignments exceeded the IGP of the original clustering, i.e. our obtained results were highly significant.

It is worthwhile mentioning that we also re-calculated the IGP for our integrated AETIONOMY AD and PD cohorts separately to exclude that the observed high coherence was only true for one of the two diseases. Figures S23, S24 clearly demonstrate that no corresponding biases could be observed, i.e. IGP values were in a comparable range.

### Statistical analysis of clusters

#### Clinical data

Clinically observed differences between patient subgroups might be impaired by multiple confounding factors. To identify these confounders, we initially performed a stepwise multinomial logistic regression (R-package “nnet”) with the cluster indicator as response and several potential confounders as predictors. This approach was chosen to account for the fact that many clinical variables show a highly skewed distribution. Considered confounders included:

- baseline diagnosis (ADNI),
- age (all),
- gender (all),
- marriage status (ADNI),
- education level (ADNI, ROSMAP, AETIONOMY PD),
- sub-study (ADNI1, ADNI2, ADNIGO, ADNI3; ROS, MAP),
- duration of the disease since the first diagnosis (PPMI, AETIONOMY PD, DIGPD, ICEBERG, ROSMAP),
- smoking history (AETIONOMY PD)
- coffee and alcohol consumption (AETIONOMY PD)
- ethnicity, including Spanish origin (PPMI, AETIONOMY PD, ICEBERG, DIGPD, ROSMAP), and
- prior neurological drug treatment (ROSMAP, PPMI, AETIONOMY PD, ICEBERG, DIGPD), including L-DOPA for PD (PPMI, AETIONOMY PD, ICEBERG, DIGPD).

The Akaike Information Criterion (AIC) was used for model selection, resulting in an “optimal” confounder set. It is worthwhile mentioning that none of the considered confounders demonstrated univariately significant association to cluster membership in any dataset according to a likelihood ratio test against the null model.

To determine the influence of clinical outcome measures (e.g. UPDRS3) in a second step we fitted a multinomial logistic regression model that included in addition to the selected confounders exactly one of the clinical variables of interest. In other words, there was a separate multinomial logistic regression for each clinical outcome measure. We then used a likelihood ratio test (Analysis of Deviance/type III ANOVA) to estimate the significance of the influence of the clinical variable of interest while correcting for confounders. In case of nominal significance (*p* < 0.05) we conducted a post-hoc analysis of pairwise differences between clusters using a Wald test. Due to multiple pairwise comparisons and the existence of several clinical variables of interest within each study, we jointly corrected P-values resulting from all statistical tests for multiple testing. This was done via Benjamini and Hochberg’s method^33^. Corresponding results are shown in Tables S9–S11.

Statistical analysis of longitudinal clinical data from ADNI and PPMI studies was performed via a generalized linear mixed model (R-package “lme4”) between each pair of clusters. For this purpose, we subtracted from each clinical outcome score its baseline value and divided by the standard deviation of the outcome score at baseline, resulting into a patient specific progression score. Two alternative approaches to model time were considered, namely either as a numerical value or as a categorical factor. Model selection via the AIC was used to choose among these alternatives. Notably, we also included an interaction effect between cluster and time to model potentially existing time inhomogeneous effects (none of them being significant, though). Furthermore, we included a random intercept for each patient. Akin to the situation for the baseline data we performed a stepwise regression to initially select an “optimal” confounder set. Afterwards, a type III ANOVA was conducted to estimate the significance of a given clinical outcome. P-values of pairwise differences between clusters were corrected for multiple testing correction in the same way as described before. Results of the clinical time series analysis are shown in Tables S12–S13.

#### Brain imaging

Statistical analysis of features derived from MRI imaging in ADNI and DaTSCAN in PPMI in principle followed the same approach as those derived from for clinical data at baseline. The only difference was that for analysis of MRI imaging derive features we always used age and sex as confounders, and no further confounders were considered. Results of the statistical analysis are shown in Tables S14–S15. For ADNI we used two types of imaging data:

1. 7 precalculated brain volume measurements available in the ADNIMERGE package. We used always data from that visit, at which the first dementia diagnosis had been given.
2. 193 subcortical brain volumes calculated from Distrieux’s parcellation approach, see details in Supplements.

All brain volume measurements were divided by intercranial volumes for normalization purposes before statistical analysis.

#### CSF biomarkers

CSF biomarkers were analyzed in the same way as clinical variables. Results are shown in Table S16. For ADNI AD patients we used always data from that visit, at which the first dementia diagnosis had been given.

#### Omics data

Analysis of transcriptomics (ROSMAP, PPMI), methylomics (ROSMAP, PPMI) data followed common practice in bioinformatics. Details are explained in the Supplements of this paper. Confounder analysis was done akin to clinical data. Accordingly, no confounders were identified in ROSMAP. However, initial quality control suggested a batch effect between the ROS and MAP sub-studies in DNA methylation data, which we corrected via ComBat^85^. In PPMI we used gender (RNAseq) and age (DNA methylation) as confounders. Complete analysis results are available under https://clus2bio.scai.fraunhofer.de/.

#### Analysis tools

We used bcftools (version 1.8) and PLINK (version 1.90b4.1) for SNP recoding from whole genome sequencing and genotyping data respectively.

R 3.5.1 was used for all data analyses purpose. R-package h2o (cluster version 3.26.0.2) was used for the autoencoder model training and logistic regression. We have used R-package NMF (version 0.21.0) for clustering. R-package biomart (version 2.36.1) was used for gene annotation. R-package SNPlocs.Hsapiens.dbSNP.20120608 (version 0.99.11) was used to map genomic coordinates to rsIDs. We have used R-package ggplot2 (version 3.0.0) for producing all the analysis plots in addition to inbuilt tool provided by R-package NMF package (version 0.21.0) for clustering and visualizations.

## Supplementary information

Supplementary Information.

## Abbreviations

A-*β*: Amyloid beta
AChE: Acetylcholinesterase
AD: Alzheimer’s disease
ADAS: Alzheimer’s disease assessment scale
ADNI: Alzheimer’s disease neuroimaging initiative
AUC: Area under the receiver operating characteristic curve
CDRSB: Clinical dementia rating sum of boxes
CSF: Cerebrospinal fluid
ESS: Epworth sleepiness scale
eQTL: Expression quantitative trait loci
FAQ: Functional activities questionnaire
GO: Gene ontology
GWAS: Genome wide association study
HADS: Hospital anxiety and depression scale
IGP: In-group proportion
KEGG: Kyoto encyclopedia of genes and genomes
MCI: Mild cognitive impairment
MMSE: Mini-mental state examination
MOCA: Montreal Cognitive Assessment
MRI: Magnetic resonance imaging
NMF: Non-negative matrix factorization
PPMI: Parkinson’s progression markers initiative
PET: Positron emission tomography
PD: Parkinson’s disease
RBD: Rapid eye movement sleep behavior disorder
SNP: Single nucleotide polymorphism
UPDRS: Unified Parkinson's disease rating scale
