# Supplementary material for: Clustering of Alzheimer’s and Parkinson’s disease based on genetic burden of shared molecular mechanisms
Source: Sci Rep. 2020 Nov 5;10:19097. doi: 10.1038/s41598-020-76200-4 (PMC7645798; doi:10.1038/s41598-020-76200-4)
Supplement: Supplementary file 1 — Supplementary Information. [file 41598_2020_76200_MOESM1_ESM.docx]

Supplementary Material: Clustering of Alzheimer’s and Parkinson’s Disease Based on Genetic Burden of Shared Molecular Mechanisms

Mohammad Asif Emon 1, 3, +), Ashley Heinson 2,+), Ping Wu 2,+), Daniel Domingo-Fernández 1, 3), Meemansa Sood 1, 3), Henri Vrooman 4), Jean-Christophe Corvol 5), Phil Scordis 2), Martin Hofmann-Apitius 1, 3), Holger Fröhlich 1, 3, 6, *)

1. Fraunhofer Institute for Algorithms and Scientific Computing (SCAI), 53754 Sankt Augustin, Germany
2. UCB Pharma (UCB Celltech Ltd.), 208 Bath Road, Slough, Berkshire, SL1 3WE, UK
3. University of Bonn, Bonn-Aachen International Center for IT, Endenicher Allee 19c, 53115 Bonn, Germany
4. Erasmus MC, University Medical Center Rotterdam, Department of Radiology & Nuclear Medicine and Department of Medical Informatics, PO Box 2040, 3000 CA Rotterdam, Netherlands
5. ICM - Hôpital Pitié Salpêtrière, 47, bd de l'hôpital, 75013 Paris, France
6. UCB Pharma (UCB Biosciences GmbH), Alfred-Nobel-Str. 10, 40789 Monheim, Germany

+) These authors contributed equally

*) correspondence: holger.froehlich@scai.fraunhofer.de

[**Common Mechanisms Identified in AD and PD**](#_jznzow5ehzr8) 2

[**SNP Genotyping, Filtering and Gene Mapping**](#_hwkuo1imbmyl) 3

[**Sparse Autoencoders for Learning SNP Burden Scores**](#_lgj1zmh2aqeo) 13

[**Consensus sNMF Clustering**](#_7bm82zctp2qi) 28

[**Validation of Patient Subtypes in Independent Studies**](#_2727vv7xx1go) 36

[**Statistical Analysis of Clusters**](#_d9yov31kgqmt) 39

[Clinical Outcome Measures](#_l55fvopxbs8f) 39

[Brain Imaging](#_drm3mjjg4v2w) 46

[CSF Biomarkers](#_t63e9r1ges9) 48

[Genome-wide Transcriptome Analysis](#_olrdnefuxnyd) 48

[Genome-wide Methylome Analysis](#_mkdcdci9hqqv) 54

[**Calculation of Subcortical Brain Volumes**](#_lzjwbiujd41) 62

[**Potential Implications for Drug Development**](#_7ot5e9fsm9p7) 62

[Alzheimer’s Disease](#_vh4242kflttm) 62

[Parkinson’s Disease](#_tehg4qpn3xko) 63

[**Study Groups**](#_vunozbzmrdq) 63

[AETIONOMY](#_m9vkoi93vlxa) 63

[DIGPD](#_asbd7240d6va) 64

[ICEBERG](#_d85fubya97q1) 65

[**References**](#_gbhluc6yp8qc) 67

# Common Mechanisms Identified in AD and PD

**Table S1:** Cause-effect relationships (aka mechanisms) commonly found in AD and PD. Mechanisms are grouped based on patient cluster. The blue color refers to those mechanisms that are most representative for a given patient cluster, according to the consensus sparse non-negative matrix factorization described later. The impact of each mechanism on the clustering was calculated as described in Section *Consensus sNMF Clustering.* The web link points to an interactive visualization of each mechanism subgraph.

| **Cluster** | **Feature ID** | **Gene(s)** | **Mechanisms** | **Description** | **Weblink** |
| --- | --- | --- | --- | --- | --- |
| **1** | 12 | AKT1 | 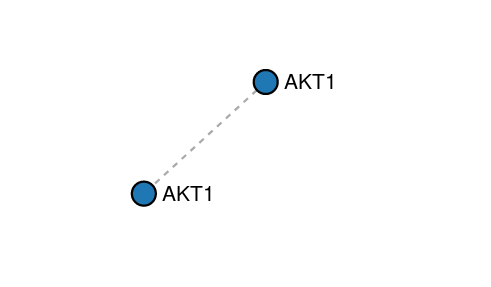 | This subgraph represents the phosphorylation of the AKT1 protein. This event regulates multiple signaling cascades and has been associated with both Alzheimer’s (AD) and Parkinson’s disease (PD) (Ohta *et al*., 2011; Ran *et al.*, 2011; Rickle *et al.*, 2004) | <https://clus2bio.scai.fraunhofer.de/visualize_mechanisms?subgraphs%5B%5D=12> |
| **2** | 1 | MAPT, CDK5, GSK3B, DYRK1A, PRKACA | 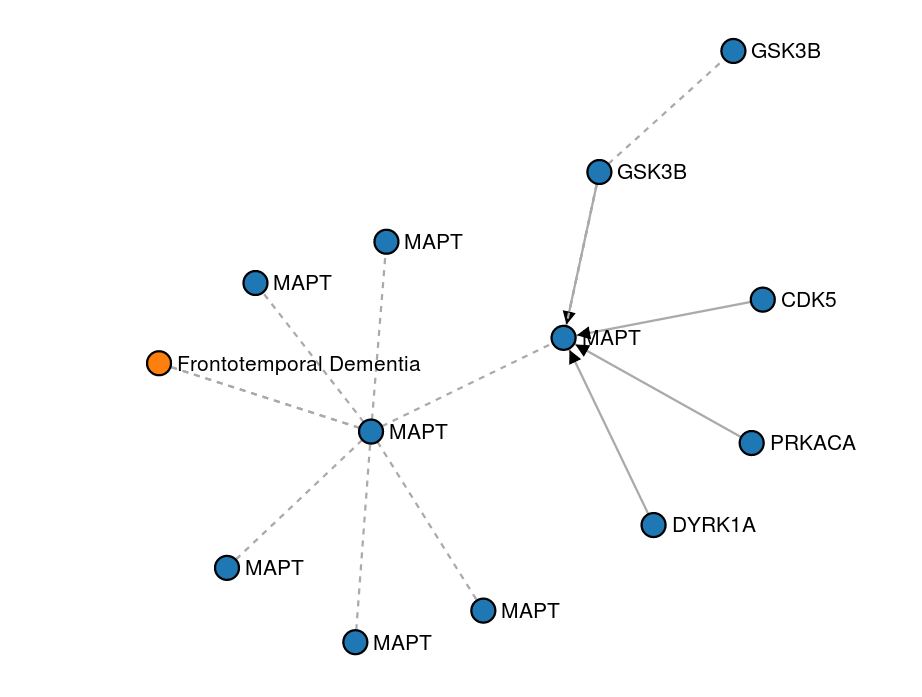 | This subgraph outlines a set of relationships across well-known key players in AD and PD that have reported in both disorders. The subgraph is centered around Tau protein different proteins that have been shown to phosphorylate it such as CDK5, GSK3B and DYRK1A. | <https://clus2bio.scai.fraunhofer.de/visualize_mechanisms?subgraphs%5B%5D=1> |
|  | 10 | IL1B, NLRP3 | 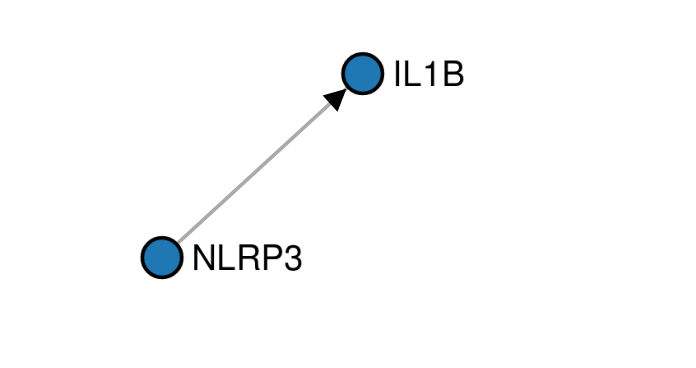 | This subgraph represents the activation of two molecules that participate in the activation of the inflammasome and other immune system and inflammatory responses. | <https://clus2bio.scai.fraunhofer.de/visualize_mechanisms?subgraphs%5B%5D=10> |
|  | 14 | MAPK8 | 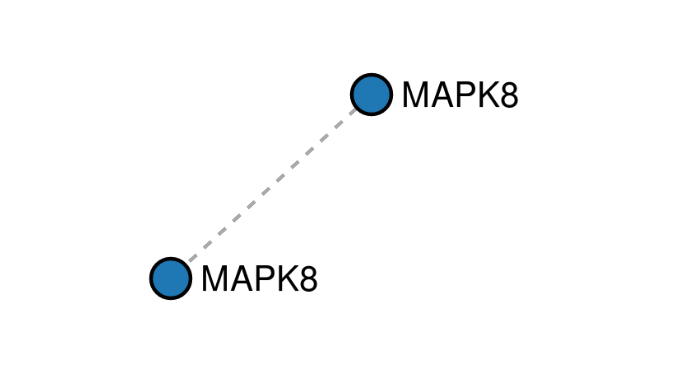 | This subgraph contains the relationship representing the activation of one of the c-Jun N-terminal kinases (JNKs). MAPK8 is involved in multiple signaling pathways such as neuronal plasticity, regeneration, apoptosis and cellular senescence. | <https://clus2bio.scai.fraunhofer.de/visualize_mechanisms?subgraphs%5B%5D=14> |
|  | 15 | H3F3A | 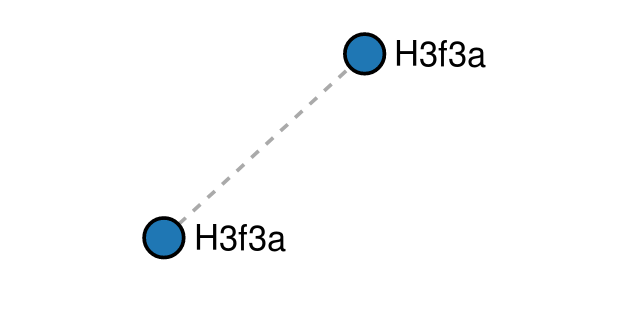 | This subgraph focuses on H3F3A one of the histones responsible for nucleosome structure. | <https://clus2bio.scai.fraunhofer.de/visualize_mechanisms?subgraphs%5B%5D=15> |
|  | 6 | IL6 | 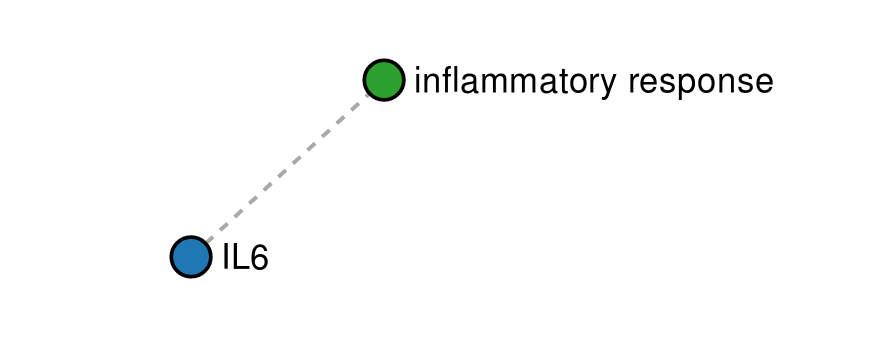 | This subgraph depicts the association between Interleukin 6 and inflammation response which has been related with both AD and PD. | <https://clus2bio.scai.fraunhofer.de/visualize_mechanisms?subgraphs%5B%5D=6> |
|  | 7 | TP53 | 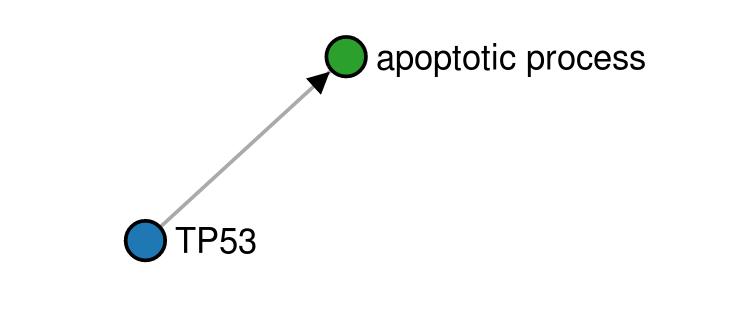 | This subgraph shows the link between p53 (TP53) and apoptosis. | <https://clus2bio.scai.fraunhofer.de/visualize_mechanisms?subgraphs%5B%5D=7> |
| **3** | 11 | DNMT1 | 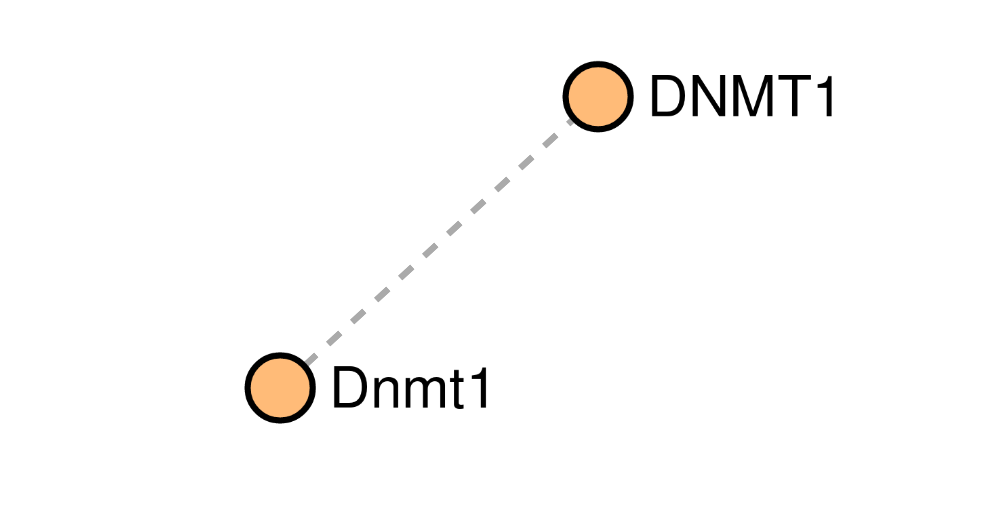 | This subgraph contains DNMT1, an enzyme responsible for DNA methylation, indirectly ensuring the maintenance of epigenetic patterns. | <https://clus2bio.scai.fraunhofer.de/visualize_mechanisms?subgraphs%5B%5D=11> |
|  | 2 | SDC2 | 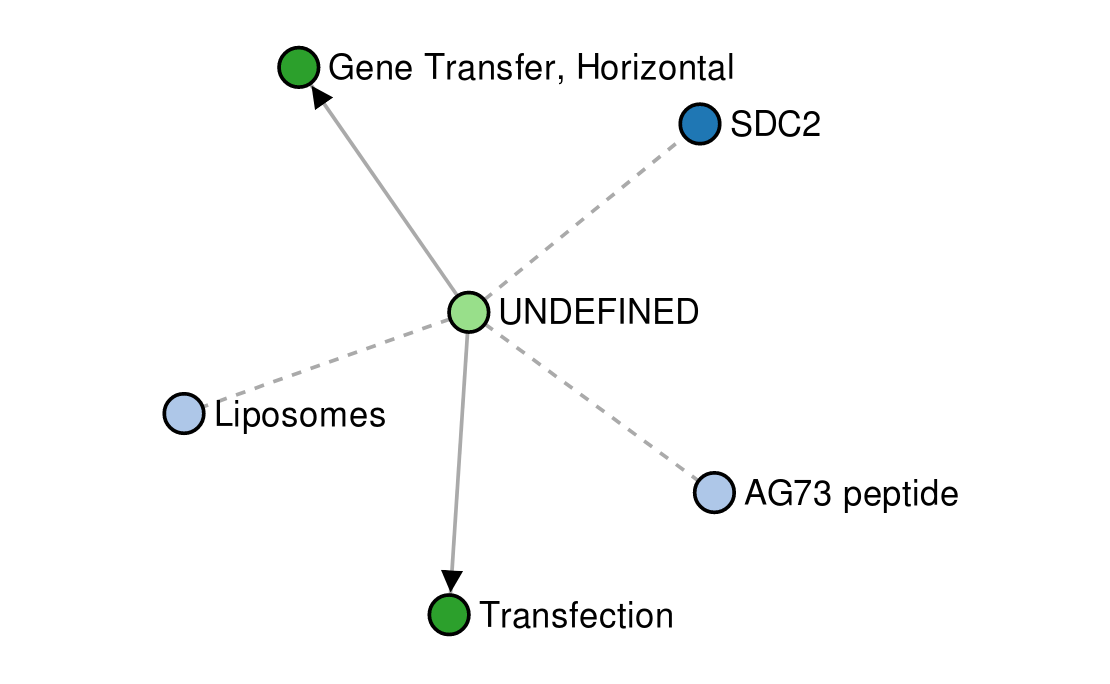 | This subgraph is centered around a member of the syndecan proteoglycan family, SDC2 which associated with AD was described by Letoha et al. | <https://clus2bio.scai.fraunhofer.de/visualize_mechanisms?subgraphs%5B%5D=2> |
|  | 3 | APOE, PICALM, TOMM40, CD33 | 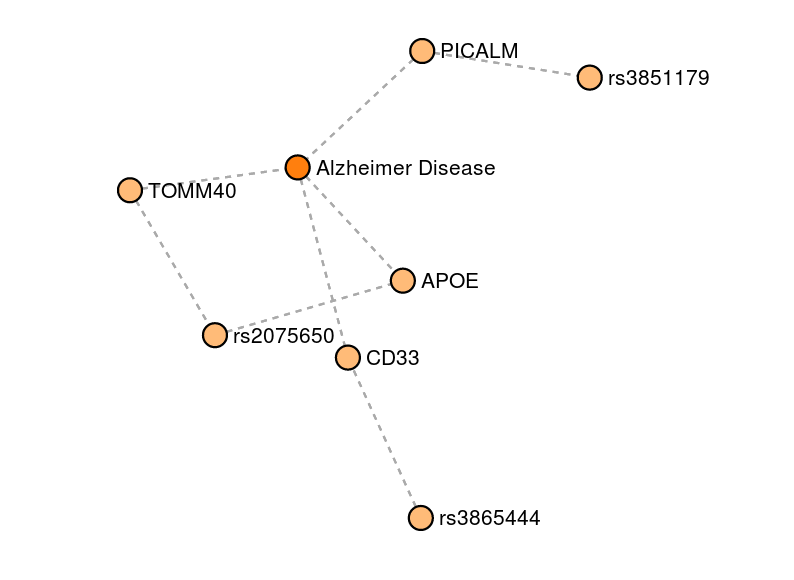 | This subgraph illustrates the interactions between genes that have been associated with both disorders (Xu *et al.,* 2016; Bradshaw *et al.,* 2013; Kim *et al.,* 2009; Lyall *et al.,* 2014). | <https://clus2bio.scai.fraunhofer.de/visualize_mechanisms?subgraphs%5B%5D=3> |
|  | 4 | SNCA, CRH, UCN | 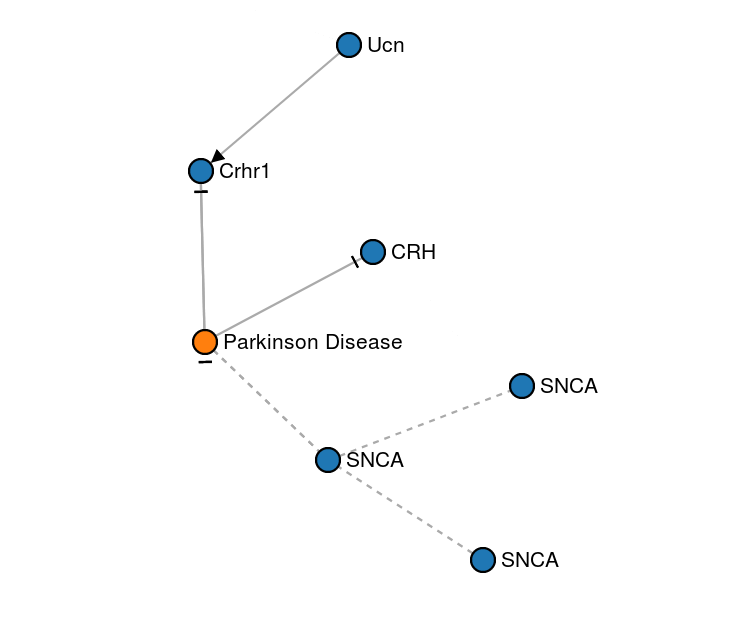 | This subgraph is centered around alpha synuclein (SNCA) one of the hallmarks of both AD and PD (Kim *et al.,* 2014). | <https://clus2bio.scai.fraunhofer.de/visualize_mechanisms?subgraphs%5B%5D=4> |
|  | 5 | MTHFR | 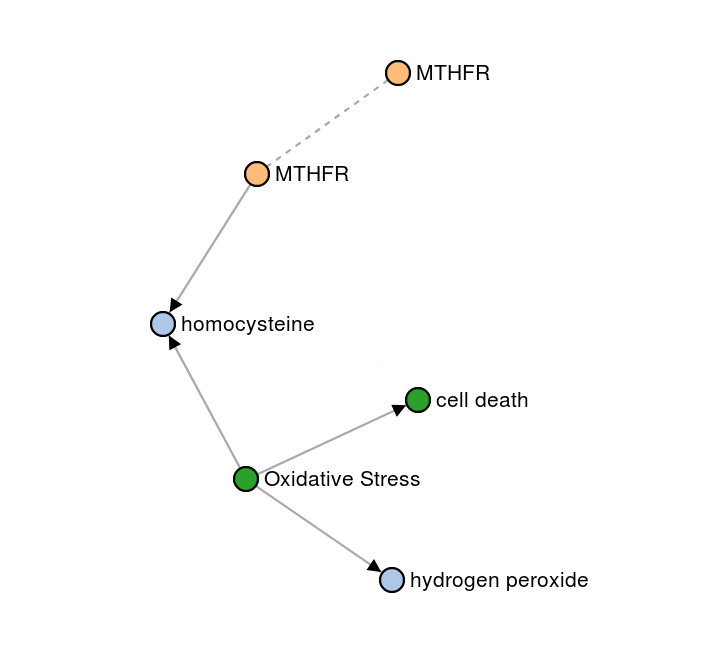 | This subgraph contains relationships between MTHFR, hydrogen peroxide and homocysteine. Moreover, the links between these molecules and other biological processes such as cell death and oxidative stress are present. These processes and molecules have been associated with both AD and PD (Roman *et al.*, 2015; Morris *et al.*, 2003; Licking *et al.*, 2017). | <https://clus2bio.scai.fraunhofer.de/visualize_mechanisms?subgraphs%5B%5D=5> |
|  | 8 | CASP3, CYCS | 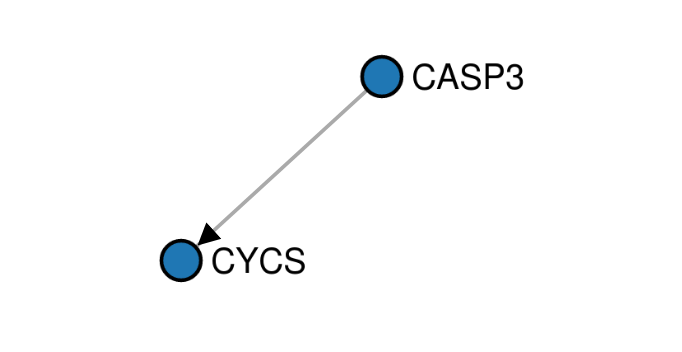 | This subgraph contains two key molecules involved in apoptosis-related processes. | <https://clus2bio.scai.fraunhofer.de/visualize_mechanisms?subgraphs%5B%5D=8> |
|  | 9 | BCL2, GDNF | 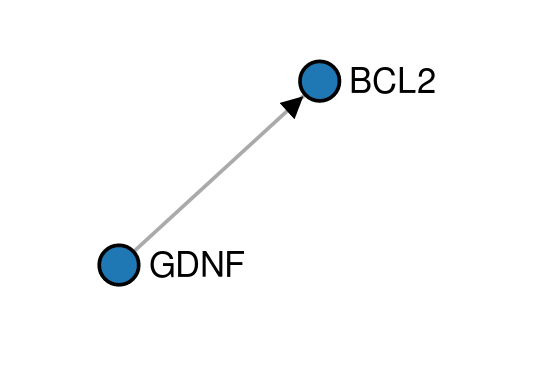 | This subgraph shows the link between the glial cell-line derived neurotrophic factor and BCL2, a main regulator of apoptotic processes. | <https://clus2bio.scai.fraunhofer.de/visualize_mechanisms?subgraphs%5B%5D=9> |
| **4** | 13 | MAPK9 | 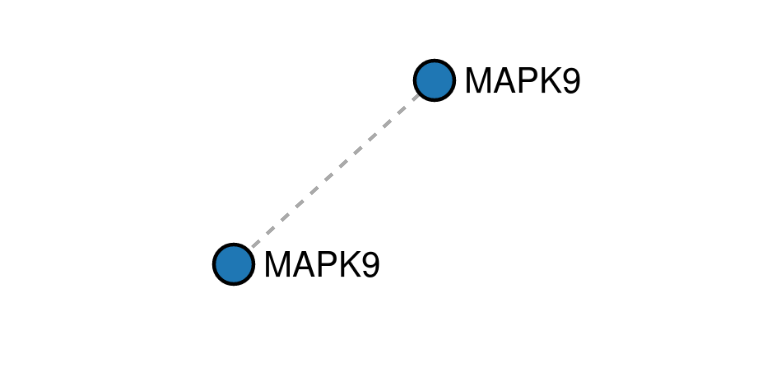 | Similar to subgraph 14, this subgraph depicts the activation of MAPK9, which is implicated in multiple signaling pathways. | <https://clus2bio.scai.fraunhofer.de/visualize_mechanisms?subgraphs%5B%5D=13> |

# SNP Genotyping, Filtering and Gene Mapping

SNP based genotype information was available in the following form for the different cohorts analyzed here:

- ADNI (discovery):
  - ADNI 1: 620,901 SNPs measured via Illumina Human610-Quad BeadChip platform
  - ADNI2/GO: 730,525 SNPs measured via Illumina HumanOmniExpress BeadChip
- PPMI (discovery): Whole Genome Sequencing was performed on whole-blood extracted DNA samples of PD cases or healthy control, and VCF ﬁles (aligned to Genome Reference Consortium Human Build 38) was downloaded from Parkinson’s Progression Markers Initiative server (<https://www.ppmi-info.org/accessdata-specimens/download-data/>). Only those variants passing all pre-deﬁned ﬁlters in the VCF ﬁle were used, which includes ExcessHet, lowQual and VQSR (Variant Quality Score Recalibration). The genotypes of SNPs were extracted, and were re-coded following common convention by the occurrence of a minor allele (0, 1, 2).
- ROSMAP (validation): Two batches of genotype data are available in ROS and MAP studies. The first batch was generated in 2009 on 1709 individuals using the Affymetrix GeneChip 6.0 (Affymetrix, Inc, Santa Clara, CA, USA) at the Broad Institute’s Center for Genotyping or the Translational Genomics Research Institute. The second batch was generated in 2012 on 382 individuals using the Illumina HumanOmniExpress (Illumina, Inc, San Diego, CA, USA) at the Children’s Hospital of Philadelphia. Only individuals with European ancestry were genotyped to minimize population heterogeneity. Sample-level quality control assessment included exclusion of samples with genotype success rate <95%, discordance between inferred and reported gender, and excess inter/intraheterozygosity. SNP-level quality control assessment included exclusion of SNPs with Hardy-Weighberg equilibrium (p<0.001), MAF < 0.01, genotype call rate < 0.95, misshap test < 1x10-9. Population outliers were identified and removed using EIGENSTRAT with default parameters. (https://www.synapse.org/#!Synapse:syn3157325).
- IDIBAPS, AETIONOMY PD, ICEBERG (validation): The AETIONOMY PD, ICEBERG and IDIBAPS cohorts have been genotyped at the ICM using Illumina NeuroX2 microarrays, and were processed jointly for quality control and imputation. Sample-level quality control steps included exclusion of individuals with <95% of genotype call rates, those who deviate >3SD from the mean heterozygosity rate of the whole cohort, those with discordance between inferred and reported gender, most related individuals (Pi-hat >0.1875) and the ethnic outliers identified with PCA approach. SNP-level quality control assessment included exclusion of SNPs with MAF < 0.001, genotype call rate < 0.98, Hardy-Weighberg equilibrium of p<1e-10 for patients and p<1e-6 for healthy controls. 615 individuals passed filters and QCs.
- DIGPD (validation): DIGPD cohort was genotyped with the Multi-Ethnic Global array (MEGACHIP). Due to the different chip platform the data was processed separately from IDIBAPS, AETIONOMY PD and ICEBERG, but the same steps of quality control and filtering were applied. 415 individuals passed filters and QCs.

For the discovery of disease subtypes we only used SNPs that were measured in both ADNI chip platforms as well as in PPMI, i.e. no imputation was used. But, SNP imputation was conducted in validation cohorts. It was carried out via the Michigan Imputation Server [(Das et al., 2016)](https://www.zotero.org/google-docs/?2wN4ze) or Sanger Imputation Service [<https://imputation.sanger.ac.uk>] using the Haplotype Reference Consortium (HRC) reference panel, which consists of 64,976 haplotypes [(The Haplotype Reference Consortium et al., 2016)](https://www.zotero.org/google-docs/?Nduot1). SNPs were considered as reliable imputed, if the $r^{2}$score was above 0.3 (default setting).

There were 57,779 SNPs jointly available in both ADNI chip platforms as well as PPMI. These SNPs were mapped to genes based on two alternative criteria:

- proximity to the closest gene (+/- 50 kbps): We downloaded all human genes with their HGNC symbols, chromosome name, start position, and end position from ENSEMBL database (using genome version hg19) by using R package ‘biomart - v 2.36.1’. Then, using SNPs genomic positions from our datasets we mapped them to the closest gene by using a window of 50 kbps either up- or down- stream of the gene start position.
- eQTL mapping based on brain tissue gene expression available from GTEx [(Carithers et al., 2015)](https://www.zotero.org/google-docs/?dpL7PY): We downloaded brain tissue-specific significant variant-gene associations from GTEx portal from the release V7. The genomic coordinates in this dataset are based on the human assembly GRCh37 (also known as hg19). A SNP was mapped to a particular gene, if there was a significant (false discovery rate < 5%) association to gene expression in any brain tissue. We used the R package ‘SNPlocs.Hsapiens.dbSNP.20120608’ (version 0.99.11) to retrieve and map to rsIDs for the variant IDs presented by genomic coordinates in the dataset. Biomart package was used as well to map ENSEMBL gene IDs in the dataset to the standard gene symbol.

Figure 1 shows the number of SNPs that were mapped to shared disease genes in that way. Table 2 shows all 148 SNPs together with their genomic location in GRCh37.


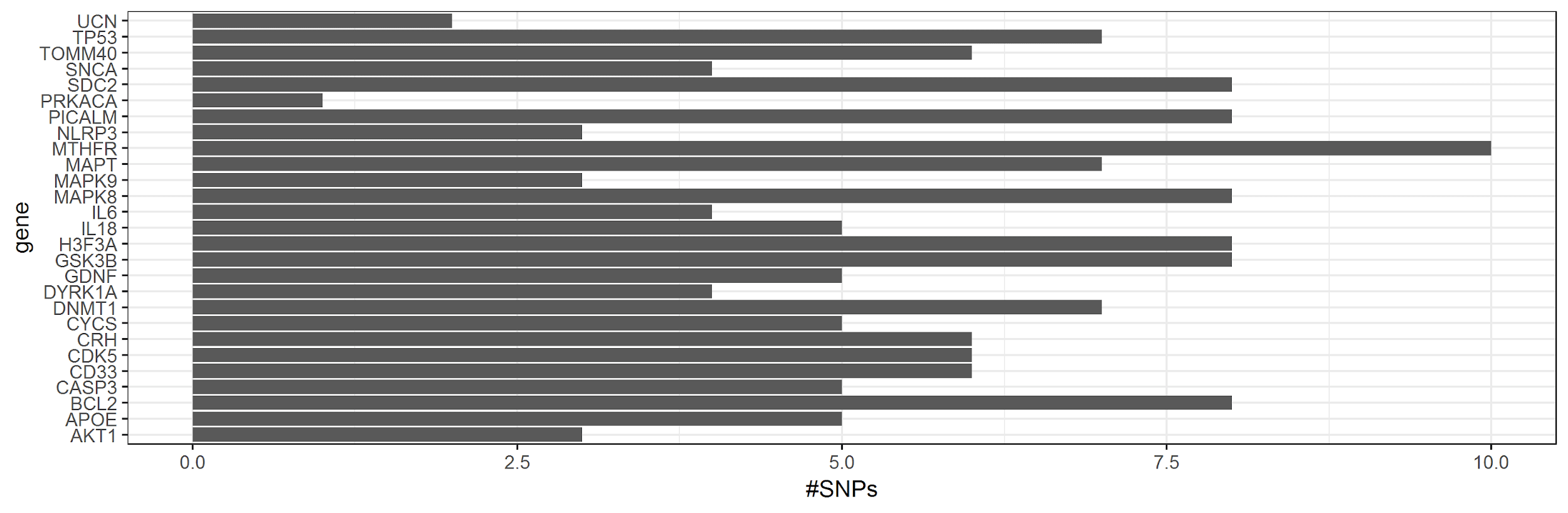


**Figure 1**: Number of SNPs mapped to genes implicated in common disease mechanisms in AD and PD.

**Table S2**: Panel of 148 SNPs together with their genomic location in GRCh37.

| **rsID** | **hgnc_symbol** | **source** | **chr** | **position (GRCh37)** | **ref** | **alt** |
| --- | --- | --- | --- | --- | --- | --- |
| **rs11160804** | **AKT1** | **proximity** | **14** | **104792254** | **G** | **A** |
| **rs4906445** | **AKT1** | **proximity** | **14** | **104780294** | **T** | **C** |
| **rs7161071** | **AKT1** | **proximity** | **14** | **104730980** | **G** | **A** |
| **rs1816466** | **APOE** | **proximity** | **19** | **44903015** | **C** | **T** |
| **rs2571151** | **APOE** | **proximity** | **19** | **44908313** | **G** | **T** |
| **rs2734453** | **APOE** | **proximity** | **19** | **44930880** | **C** | **T** |
| **rs2734456** | **APOE** | **proximity** | **19** | **44930348** | **C** | **T** |
| **rs2734459** | **APOE** | **proximity** | **19** | **44928885** | **G** | **T** |
| **rs11151202** | **BCL2** | **proximity** | **18** | **63207873** | **A** | **G** |
| **rs12327472** | **BCL2** | **proximity** | **18** | **63170422** | **T** | **C** |
| **rs17074957** | **BCL2** | **proximity** | **18** | **63204981** | **T** | **C** |
| **rs2012927** | **BCL2** | **proximity** | **18** | **63297672** | **A** | **G** |
| **rs2156058** | **BCL2** | **proximity** | **18** | **63312311** | **G** | **T** |
| **rs4632239** | **BCL2** | **proximity** | **18** | **63141287** | **C** | **T** |
| **rs4941343** | **BCL2** | **proximity** | **18** | **63196837** | **G** | **A** |
| **rs8091665** | **BCL2** | **proximity** | **18** | **63151948** | **G** | **T** |
| **rs10028767** | **CASP3** | **proximity** | **4** | **184688001** | **T** | **G** |
| **rs4241780** | **CASP3** | **proximity** | **4** | **184638760** | **T** | **C** |
| **rs4400034** | **CASP3** | **proximity** | **4** | **184586738** | **T** | **G** |
| **rs4568278** | **CASP3** | **proximity** | **4** | **184689417** | **G** | **A** |
| **rs4862251** | **CASP3** | **proximity** | **4** | **184687644** | **T** | **C** |
| **rs1465557** | **CD33** | **proximity** | **19** | **51281225** | **T** | **G** |
| **rs4802740** | **CD33** | **proximity** | **19** | **51263432** | **G** | **A** |
| **rs7248759** | **CD33** | **proximity** | **19** | **51263510** | **T** | **G** |
| **rs7253017** | **CD33** | **proximity** | **19** | **51280756** | **G** | **A** |
| **rs8101336** | **CD33** | **proximity** | **19** | **51254550** | **A** | **G** |
| **rs919276** | **CD33** | **proximity** | **19** | **51231068** | **A** | **G** |
| **rs11761588** | **CDK5** | **proximity** | **7** | **151094048** | **T** | **C** |
| **rs2108726** | **CDK5** | **proximity** | **7** | **151069411** | **A** | **G** |
| **rs378140** | **CDK5** | **proximity** | **7** | **151009517** | **A** | **G** |
| **rs432519** | **CDK5** | **proximity** | **7** | **151013263** | **C** | **A** |
| **rs446886** | **CDK5** | **proximity** | **7** | **151053043** | **A** | **G** |
| **rs6957858** | **CDK5** | **proximity** | **7** | **151068262** | **C** | **T** |
| **rs10099098** | **CRH** | **proximity** | **8** | **66134803** | **A** | **G** |
| **rs1877829** | **CRH** | **proximity** | **8** | **66188263** | **G** | **A** |
| **rs4236939** | **CRH** | **proximity** | **8** | **66148098** | **C** | **T** |
| **rs4737206** | **CRH** | **proximity** | **8** | **66129990** | **C** | **T** |
| **rs7017178** | **CRH** | **proximity** | **8** | **66151929** | **A** | **G** |
| **rs7815138** | **CRH** | **proximity** | **8** | **66180525** | **G** | **A** |
| **rs10275560** | **CYCS** | **proximity** | **7** | **25071824** | **T** | **C** |
| **rs39425** | **CYCS** | **proximity** | **7** | **25105552** | **A** | **G** |
| **rs39431** | **CYCS** | **proximity** | **7** | **25109146** | **A** | **C** |
| **rs39438** | **CYCS** | **proximity** | **7** | **25119752** | **A** | **C** |
| **rs39446** | **CYCS** | **proximity** | **7** | **25128788** | **T** | **C** |
| **rs11883054** | **DNMT1** | **proximity** | **19** | **10157219** | **T** | **G** |
| **rs1551570** | **DNMT1** | **proximity** | **19** | **10218030** | **C** | **T** |
| **rs1673130** | **DNMT1** | **proximity** | **19** | **10135687** | **T** | **C** |
| **rs2305795** | **DNMT1** | **proximity** | **19** | **10226052** | **G** | **A** |
| **rs4804570** | **DNMT1** | **eqtl** | **19** | **11256059** | **C** | **T** |
| **rs8113341** | **DNMT1** | **proximity** | **19** | **10165932** | **A** | **G** |
| **rs9749171** | **DNMT1** | **proximity** | **19** | **10135711** | **C** | **T** |
| **rs2226827** | **DYRK1A** | **proximity** | **21** | **37494010** | **G** | **A** |
| **rs2239567** | **DYRK1A** | **proximity** | **21** | **37536408** | **T** | **C** |
| **rs2835228** | **DYRK1A** | **proximity** | **21** | **37375643** | **C** | **T** |
| **rs2835286** | **DYRK1A** | **proximity** | **21** | **37518362** | **A** | **G** |
| **rs1019496** | **GDNF** | **proximity** | **5** | **37773877** | **A** | **G** |
| **rs2216711** | **GDNF** | **proximity** | **5** | **37828844** | **C** | **T** |
| **rs2910709** | **GDNF** | **proximity** | **5** | **37811864** | **T** | **C** |
| **rs2910718** | **GDNF** | **proximity** | **5** | **37790089** | **T** | **C** |
| **rs3096140** | **GDNF** | **proximity** | **5** | **37832833** | **C** | **T** |
| **rs1147683** | **GSK3B** | **proximity** | **3** | **120083854** | **A** | **G** |
| **rs1147695** | **GSK3B** | **proximity** | **3** | **120119446** | **G** | **A** |
| **rs11929146** | **GSK3B** | **proximity** | **3** | **119943453** | **A** | **C** |
| **rs1488762** | **GSK3B** | **proximity** | **3** | **119893257** | **C** | **T** |
| **rs1488763** | **GSK3B** | **proximity** | **3** | **119884663** | **C** | **A** |
| **rs1733354** | **GSK3B** | **proximity** | **3** | **120100054** | **G** | **A** |
| **rs2199503** | **GSK3B** | **proximity** | **3** | **119778489** | **A** | **G** |
| **rs334543** | **GSK3B** | **proximity** | **3** | **119832621** | **C** | **A** |
| **rs10915924** | **H3F3A** | **eqtl** | **1** | **226234883** | **C** | **T** |
| **rs10915958** | **H3F3A** | **eqtl** | **1** | **226389655** | **G** | **A** |
| **rs2671272** | **H3F3A** | **proximity** | **1** | **226015116** | **T** | **C** |
| **rs2740170** | **H3F3A** | **proximity** | **1** | **226024797** | **T** | **C** |
| **rs3008192** | **H3F3A** | **eqtl** | **1** | **226404457** | **G** | **A** |
| **rs360097** | **H3F3A** | **proximity** | **1** | **226065755** | **C** | **A** |
| **rs360102** | **H3F3A** | **proximity** | **1** | **226067862** | **G** | **A** |
| **rs6701318** | **H3F3A** | **eqtl** | **1** | **226239830** | **C** | **T** |
| **rs2518369** | **IL18** | **proximity** | **11** | **112138835** | **T** | **C** |
| **rs2564872** | **IL18** | **proximity** | **11** | **112123095** | **T** | **C** |
| **rs2564881** | **IL18** | **proximity** | **11** | **112139158** | **T** | **C** |
| **rs4937257** | **IL18** | **proximity** | **11** | **112183408** | **A** | **G** |
| **rs919479** | **IL18** | **proximity** | **11** | **112108556** | **C** | **A** |
| **rs1476482** | **IL6** | **proximity** | **7** | **22730709** | **G** | **A** |
| **rs1989838** | **IL6** | **proximity** | **7** | **22701167** | **A** | **C** |
| **rs2905325** | **IL6** | **proximity** | **7** | **22712877** | **G** | **A** |
| **rs2961294** | **IL6** | **proximity** | **7** | **22695031** | **T** | **C** |
| **rs12414155** | **MAPK8** | **proximity** | **10** | **48471020** | **T** | **C** |
| **rs2853838** | **MAPK8** | **proximity** | **10** | **48431110** | **A** | **C** |
| **rs2853840** | **MAPK8** | **proximity** | **10** | **48442146** | **A** | **G** |
| **rs4075854** | **MAPK8** | **proximity** | **10** | **48485956** | **A** | **G** |
| **rs4922511** | **MAPK8** | **proximity** | **10** | **48402818** | **A** | **G** |
| **rs7093975** | **MAPK8** | **proximity** | **10** | **48424759** | **T** | **C** |
| **rs878263** | **MAPK8** | **proximity** | **10** | **48456176** | **C** | **T** |
| **rs9421742** | **MAPK8** | **proximity** | **10** | **48483044** | **C** | **T** |
| **rs2619756** | **MAPK9** | **proximity** | **5** | **180285668** | **C** | **T** |
| **rs6882940** | **MAPK9** | **eqtl** | **5** | **179648989** | **T** | **C** |
| **rs7737438** | **MAPK9** | **eqtl** | **5** | **179619222** | **C** | **A** |
| **rs1635291** | **MAPT** | **eqtl** | **17** | **43751913** | **C** | **T** |
| **rs2001381** | **MAPT** | **proximity** | **17** | **45940302** | **T** | **C** |
| **rs415430** | **MAPT** | **eqtl** | **17** | **44859144** | **G** | **A** |
| **rs417968** | **MAPT** | **eqtl** | **17** | **43728376** | **C** | **T** |
| **rs7220419** | **MAPT** | **proximity** | **17** | **45908405** | **C** | **T** |
| **rs7224296** | **MAPT** | **eqtl** | **17** | **44800046** | **G** | **A** |
| **rs991108** | **MAPT** | **proximity** | **17** | **45887142** | **C** | **T** |
| **rs12095517** | **MTHFR** | **proximity** | **1** | **11797186** | **T** | **C** |
| **rs4073574** | **MTHFR** | **proximity** | **1** | **11802434** | **G** | **T** |
| **rs4845875** | **MTHFR** | **proximity** | **1** | **11824133** | **A** | **C** |
| **rs4845877** | **MTHFR** | **proximity** | **1** | **11824303** | **T** | **C** |
| **rs4845877** | **MTHFR** | **eqtl** | **1** | **11824303** | **T** | **C** |
| **rs4845881** | **MTHFR** | **eqtl** | **1** | **11828319** | **G** | **A** |
| **rs4845881** | **MTHFR** | **proximity** | **1** | **11828319** | **G** | **A** |
| **rs4846028** | **MTHFR** | **proximity** | **1** | **11779499** | **T** | **C** |
| **rs4846048** | **MTHFR** | **proximity** | **1** | **11846252** | **G** | **A** |
| **rs6541003** | **MTHFR** | **eqtl** | **1** | **11855867** | **G** | **A** |
| **rs6541003** | **MTHFR** | **proximity** | **1** | **11855867** | **G** | **A** |
| **rs6687381** | **MTHFR** | **proximity** | **1** | **11768869** | **A** | **C** |
| **rs7539455** | **MTHFR** | **proximity** | **1** | **11754168** | **G** | **T** |
| **rs1778541** | **NLRP3** | **proximity** | **1** | **247419664** | **C** | **T** |
| **rs2793289** | **NLRP3** | **proximity** | **1** | **247401645** | **G** | **A** |
| **rs7555638** | **NLRP3** | **proximity** | **1** | **247371918** | **T** | **C** |
| **rs10792839** | **PICALM** | **proximity** | **11** | **85938133** | **C** | **A** |
| **rs10792845** | **PICALM** | **proximity** | **11** | **85986518** | **G** | **A** |
| **rs10792847** | **PICALM** | **proximity** | **11** | **86053419** | **A** | **G** |
| **rs2374721** | **PICALM** | **proximity** | **11** | **85957745** | **A** | **G** |
| **rs292092** | **PICALM** | **proximity** | **11** | **86086581** | **A** | **G** |
| **rs292109** | **PICALM** | **proximity** | **11** | **86098680** | **A** | **G** |
| **rs3851182** | **PICALM** | **proximity** | **11** | **85908537** | **T** | **C** |
| **rs7105671** | **PICALM** | **proximity** | **11** | **86101234** | **G** | **A** |
| **rs1982632** | **PRKACA** | **proximity** | **19** | **14139004** | **T** | **C** |
| **rs2221222** | **SDC2** | **proximity** | **8** | **96452712** | **T** | **G** |
| **rs2318876** | **SDC2** | **proximity** | **8** | **96593539** | **T** | **G** |
| **rs2318878** | **SDC2** | **proximity** | **8** | **96583551** | **C** | **A** |
| **rs2873824** | **SDC2** | **proximity** | **8** | **96594769** | **T** | **C** |
| **rs3104965** | **SDC2** | **proximity** | **8** | **96594137** | **G** | **A** |
| **rs4524756** | **SDC2** | **proximity** | **8** | **96651499** | **G** | **T** |
| **rs6471533** | **SDC2** | **proximity** | **8** | **96470300** | **G** | **A** |
| **rs7011044** | **SDC2** | **proximity** | **8** | **96551404** | **A** | **G** |
| **rs11097200** | **SNCA** | **proximity** | **4** | **89761563** | **T** | **C** |
| **rs11735482** | **SNCA** | **proximity** | **4** | **89776246** | **C** | **T** |
| **rs1965869** | **SNCA** | **proximity** | **4** | **89677537** | **T** | **C** |
| **rs2609255** | **SNCA** | **proximity** | **4** | **89811195** | **G** | **T** |
| **rs1816466** | **TOMM40** | **proximity** | **19** | **44903015** | **C** | **T** |
| **rs2571104** | **TOMM40** | **proximity** | **19** | **44840789** | **A** | **G** |
| **rs2571151** | **TOMM40** | **proximity** | **19** | **44908313** | **G** | **T** |
| **rs2734453** | **TOMM40** | **proximity** | **19** | **44930880** | **C** | **T** |
| **rs2734456** | **TOMM40** | **proximity** | **19** | **44930348** | **C** | **T** |
| **rs2734459** | **TOMM40** | **proximity** | **19** | **44928885** | **G** | **T** |
| **rs11656201** | **TP53** | **proximity** | **17** | **7672047** | **C** | **A** |
| **rs2278637** | **TP53** | **eqtl** | **17** | **8062102** | **G** | **T** |
| **rs307627** | **TP53** | **proximity** | **17** | **7625063** | **T** | **C** |
| **rs4233018** | **TP53** | **proximity** | **17** | **7698599** | **G** | **A** |
| **rs4791806** | **TP53** | **proximity** | **17** | **7729184** | **T** | **C** |
| **rs7141** | **TP53** | **proximity** | **17** | **7614601** | **G** | **A** |
| **rs7213894** | **TP53** | **proximity** | **17** | **7722365** | **C** | **T** |
| **rs11126836** | **UCN** | **proximity** | **2** | **27273128** | **C** | **T** |
| **rs1866561** | **UCN** | **proximity** | **2** | **27351279** | **G** | **A** |

# Sparse Autoencoders for Learning SNP Burden Scores

An autoencoder is a special kind of neural network, which takes a feature vector$x\mathfrak{\in R}{}^{d}$ as input and transforms / encodes it to a hidden representation $\underline{x}\mathfrak{\in R}{}^{q}$via

$\underline{x}=s(Wx+b)$ (1)

where $s(\cdot)$is a non-linear activation function, e.g. sigmoid or rectified linear unit. Matrix $W$ consists of weights and $b$ is a bias vector. Several encoding steps can be performed sequentially, resulting into a deep autoencoder. The latent representation $\underline{x}$ can be decoded / mapped back via

$z=s(W{{}^{T}\underline{x}+b}){}^{T}$ (2)

Standard autoencoders are trained to minimize the difference between reconstructions $z$ and original inputs $x$. In our case the mean squared error (MSE) was used for that purpose. Sparsity w.r.t. inputs (i.e. SNPs) can be enforced by introducing drop-out units into the input layer of the autoencoder network [(Srivastava et al., 2014)](https://www.zotero.org/google-docs/?cp5oUP). In addition, we used an $l_{2}$ penalty for all weights.

Tuning hyper-parameters of autoencoder networks included the activation function (rectified linear unit or hyperbolic tangent), the input dropout ratio (0%, 5%, 20%, 50%), $l_{2}$-penalty (10^-4^, 10^-3^, …, 10^4^), and the network architecture. More specifically, we tested the following architectures:

● one hidden layer with one hidden unit

● two hidden layers: first layer with n/2 units, second with one hidden unit (n = number of SNPs mapping to a particular mechanism)

● three hidden layers: first layer with n/2 units, second with n/4 units and third with one hidden unit

For each combination of hyper-parameters a separate autoencoder training was performed for at most 500 epochs, but stopped earlier, if the reconstruction error (here: mean squared error = MSE) did not improve for 5 rounds. The best autoencoder model was selected according to the 5-fold cross-validated MSE criterion. We here relied on the h2o autoencoder implementation (<http://docs.h2o.ai/>). In particular, h2o allows for analyzing the contribution of each individual SNP to autoencoded mechanism scores via the Gedeon method [(Gedeon, 1997)](https://www.zotero.org/google-docs/?NmL1E6). Results are shown in Figures S2 - S16.

It is worthwhile to mention that a ready trained autoencoder allows for mapping unseen test data via Eq. (1).


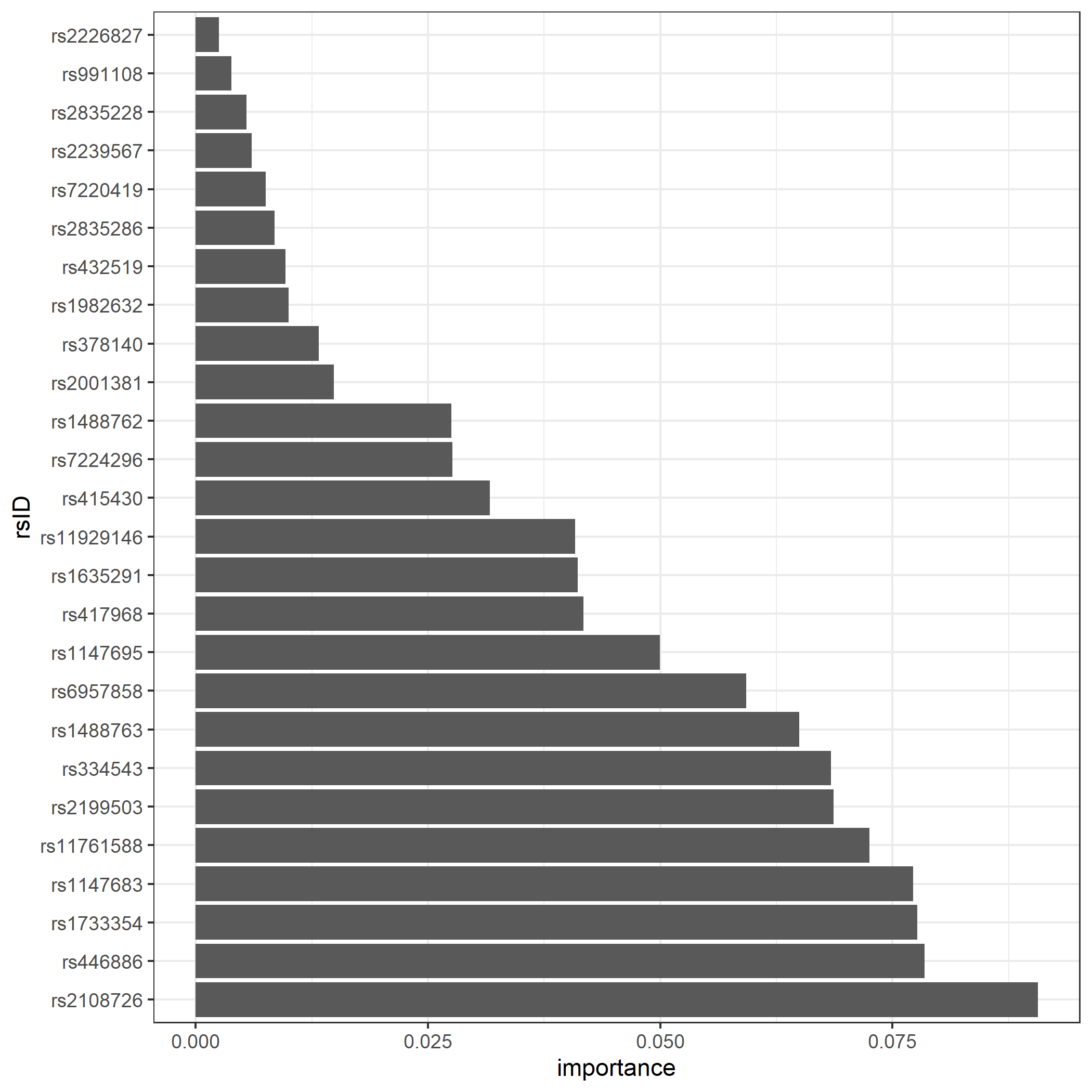


**Figure S2**: Relative impact of SNPs on mechanism 1.


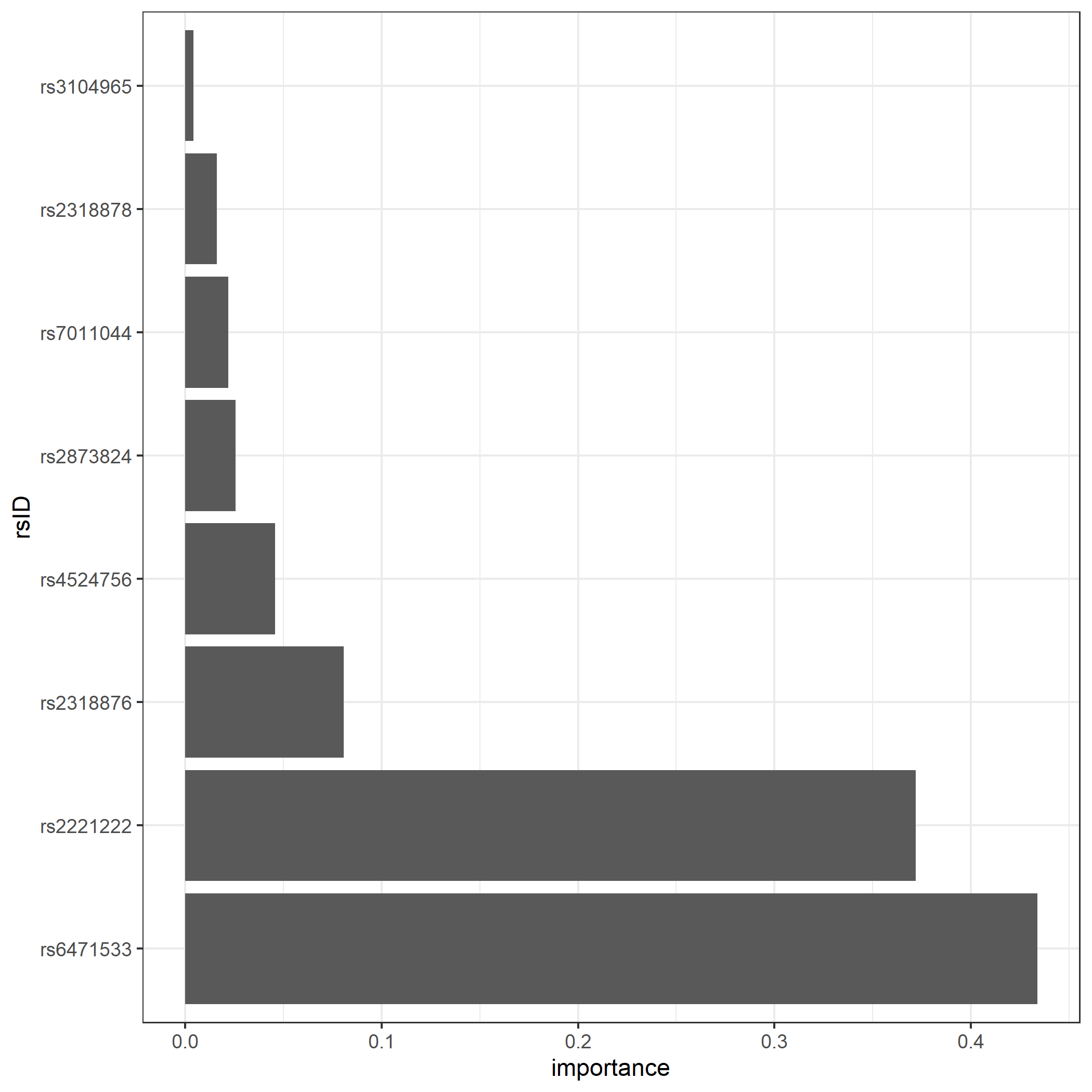


**Figure S3**: Relative impact of SNPs on mechanism 2.


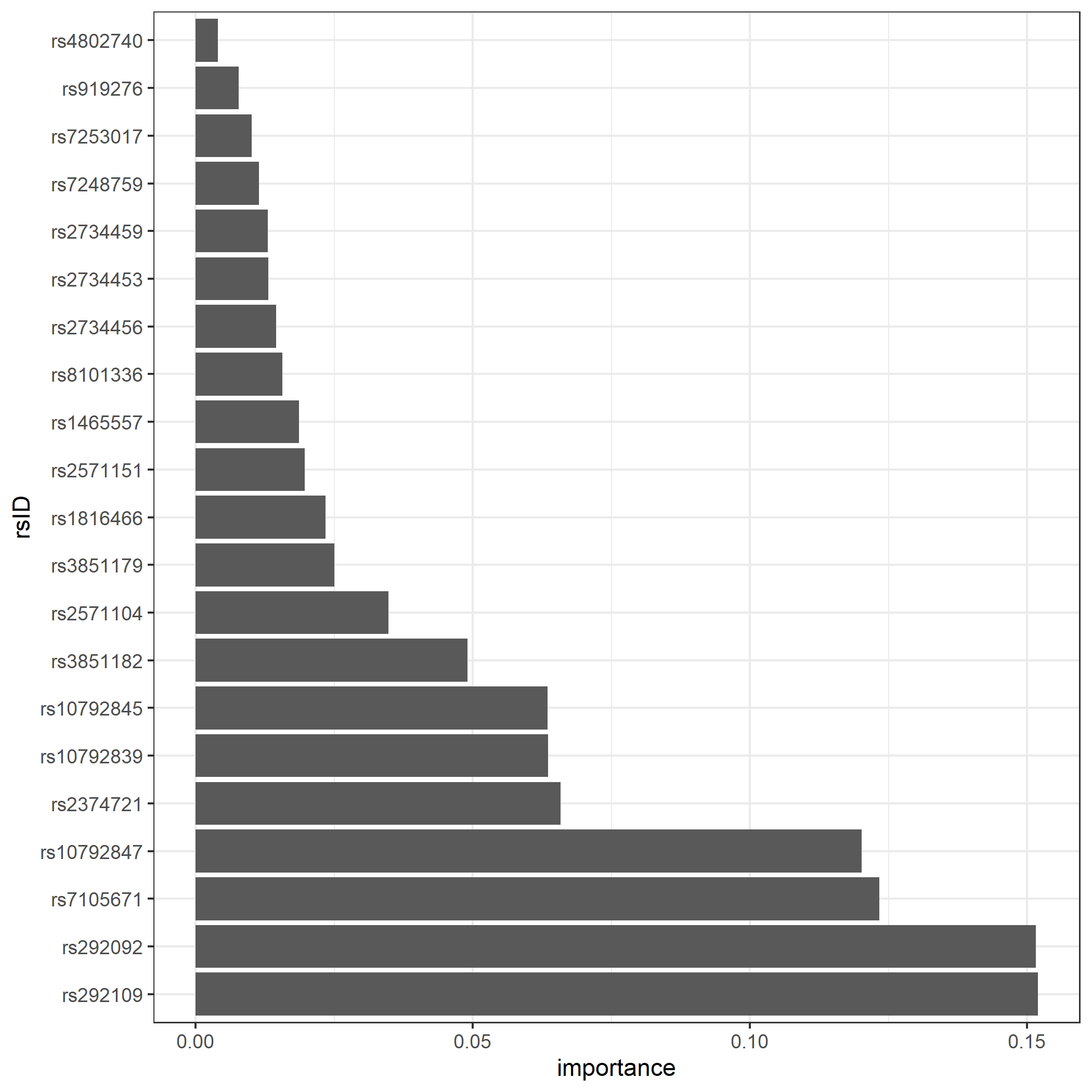


**Figure S4**: Relative impact of SNPs on mechanism 3.


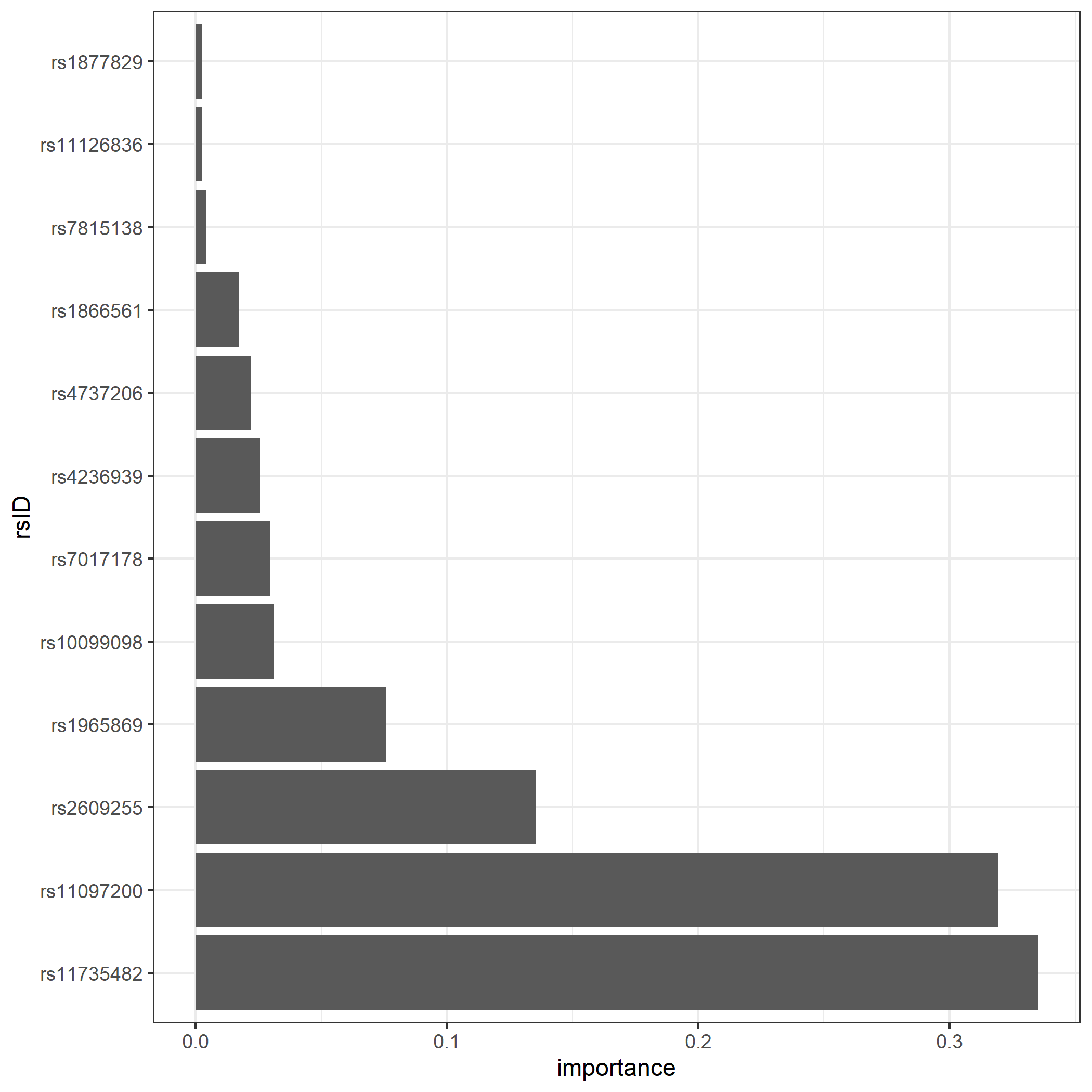


**Figure S5**: Relative impact of SNPs on mechanism 4.


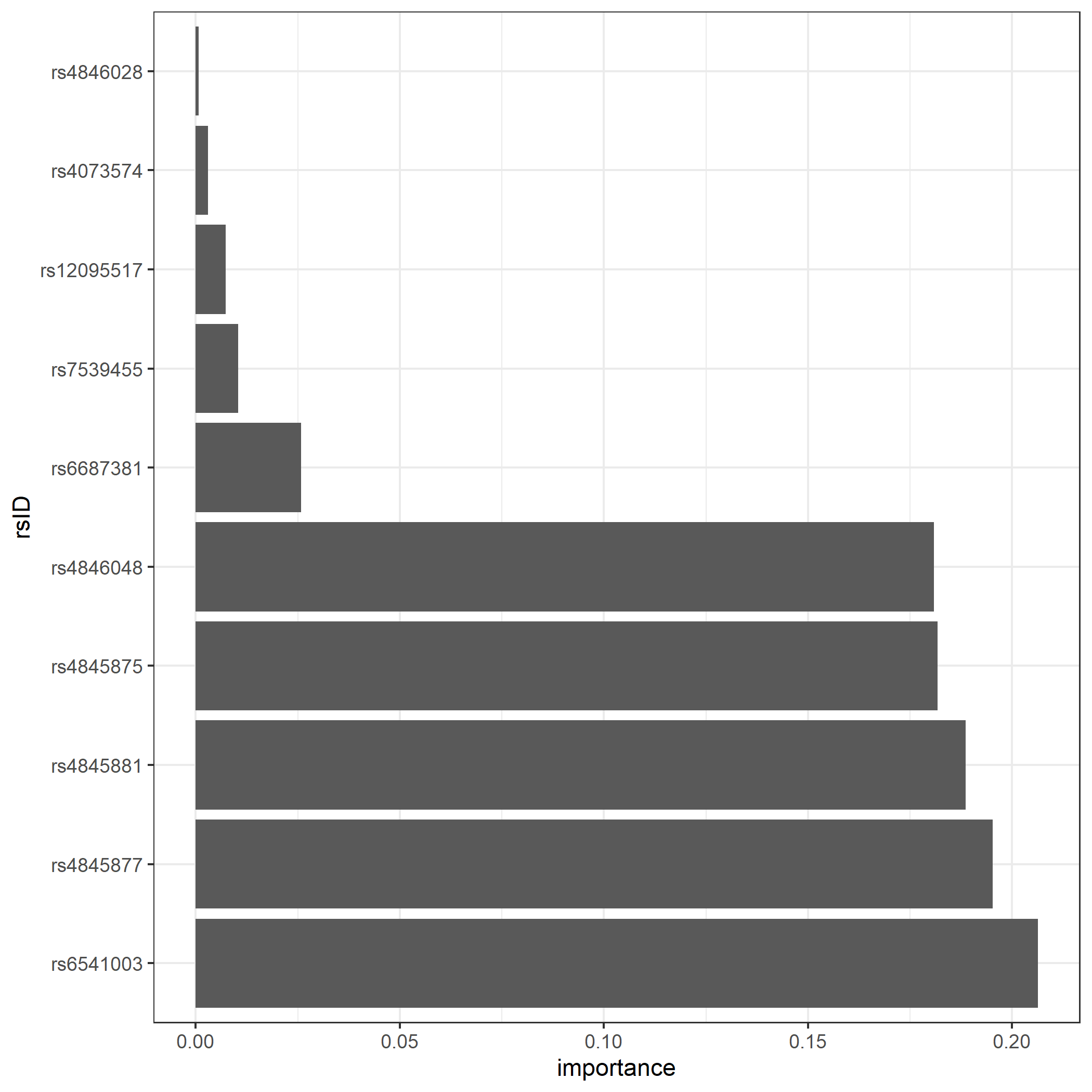


**Figure S6**: Relative impact of SNPs on mechanism 5.


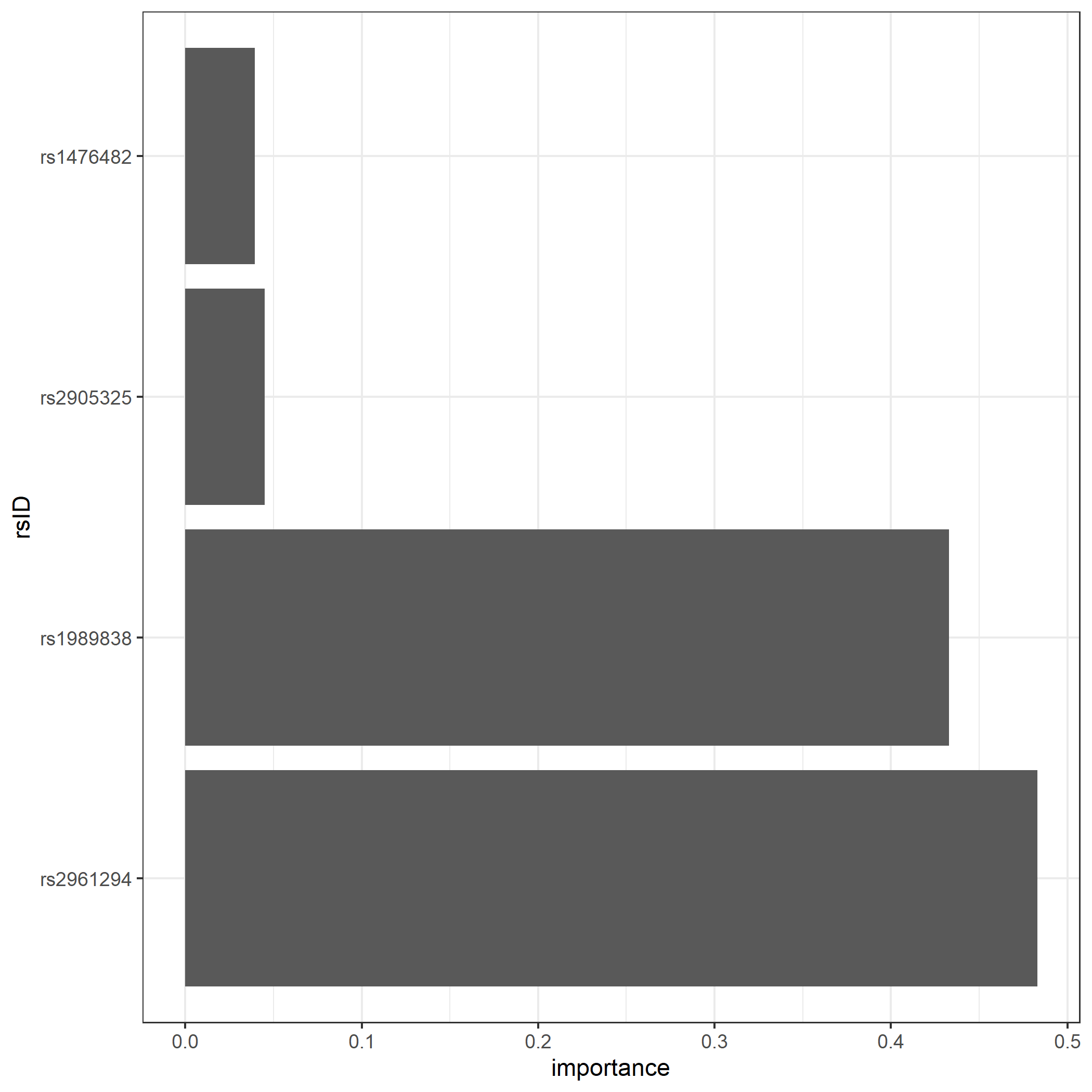


**Figure S7**: Relative impact of SNPs on mechanism 6.


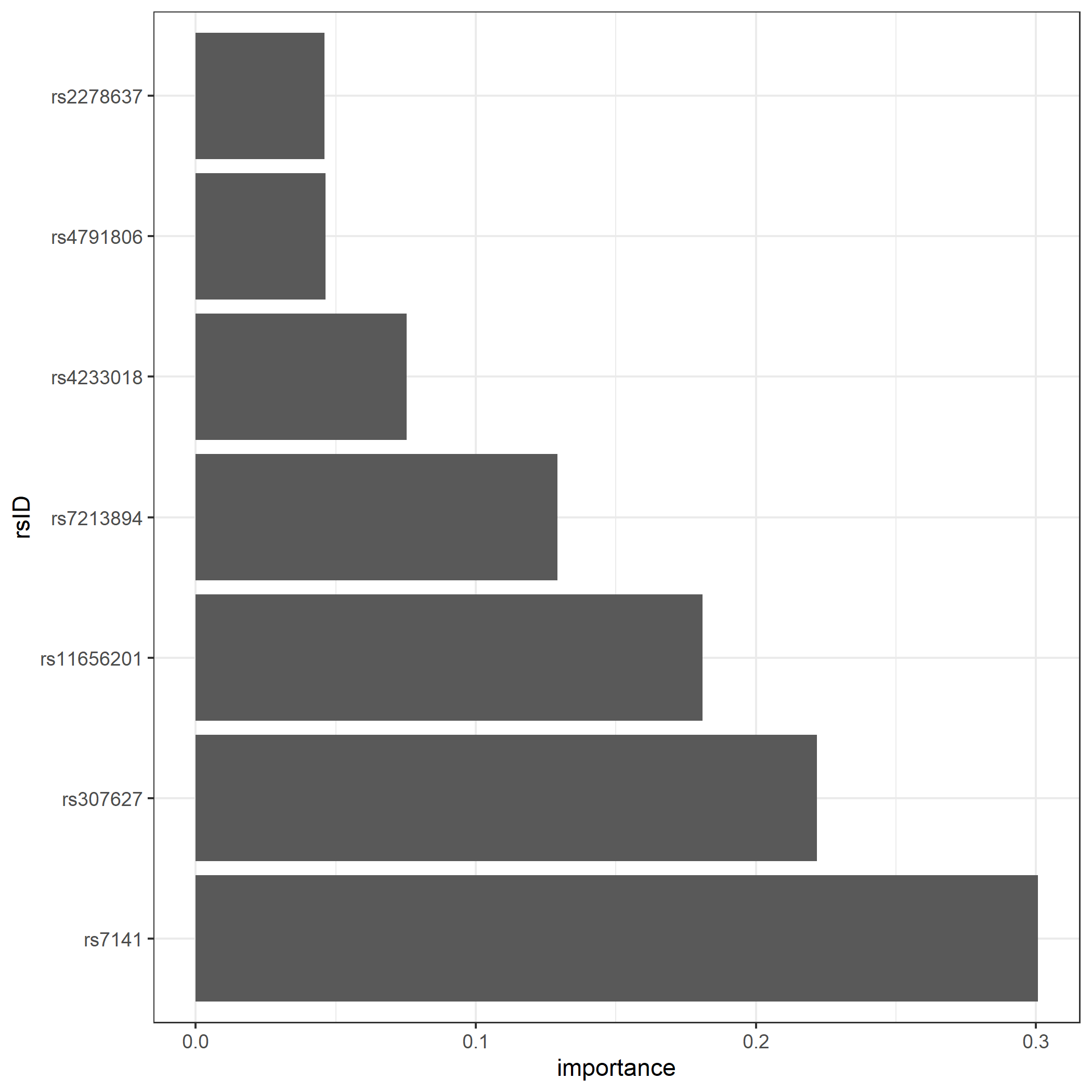


**Figure S8**: Relative impact of SNPs on mechanism 7.


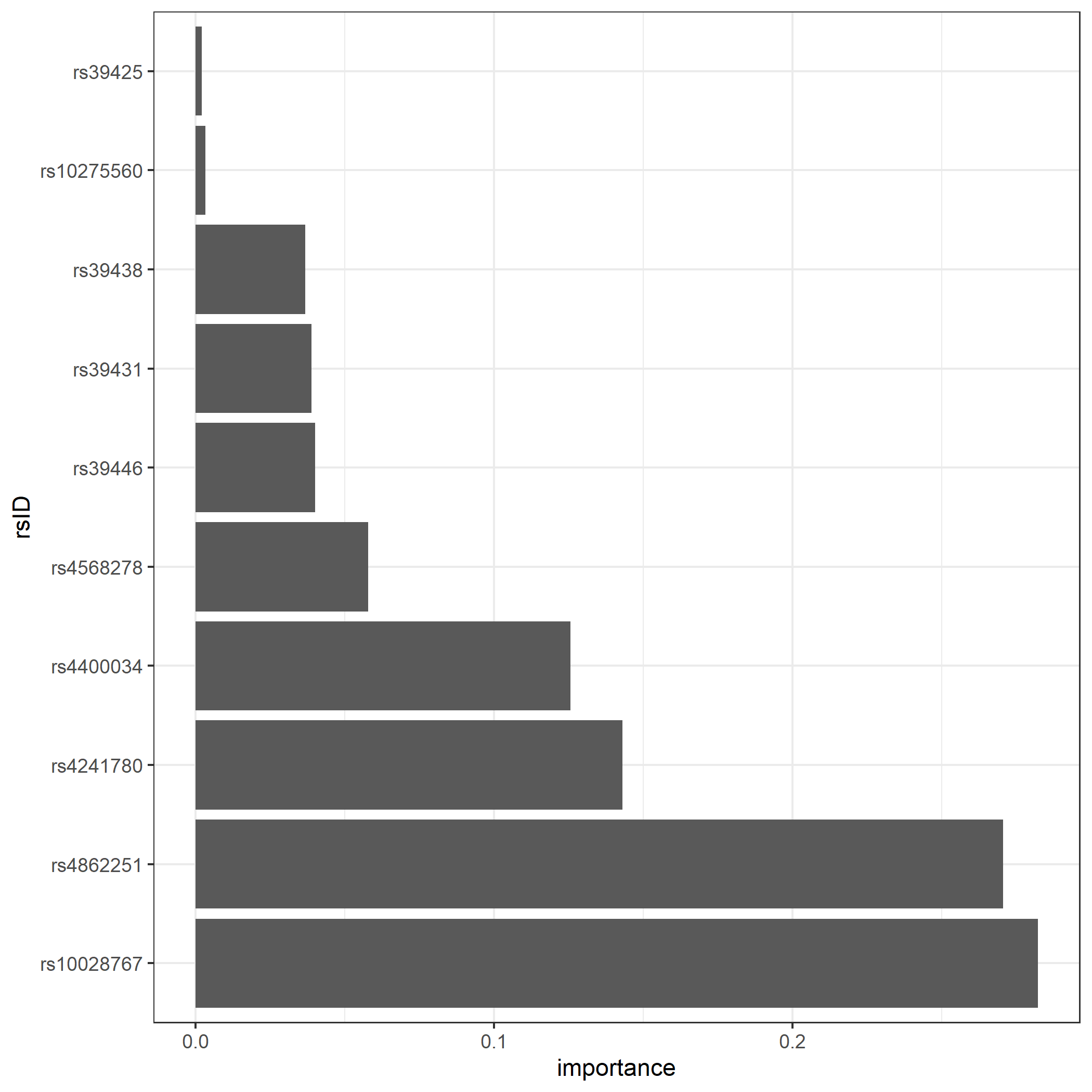


**Figure S9**: Relative impact of SNPs on mechanism 8.


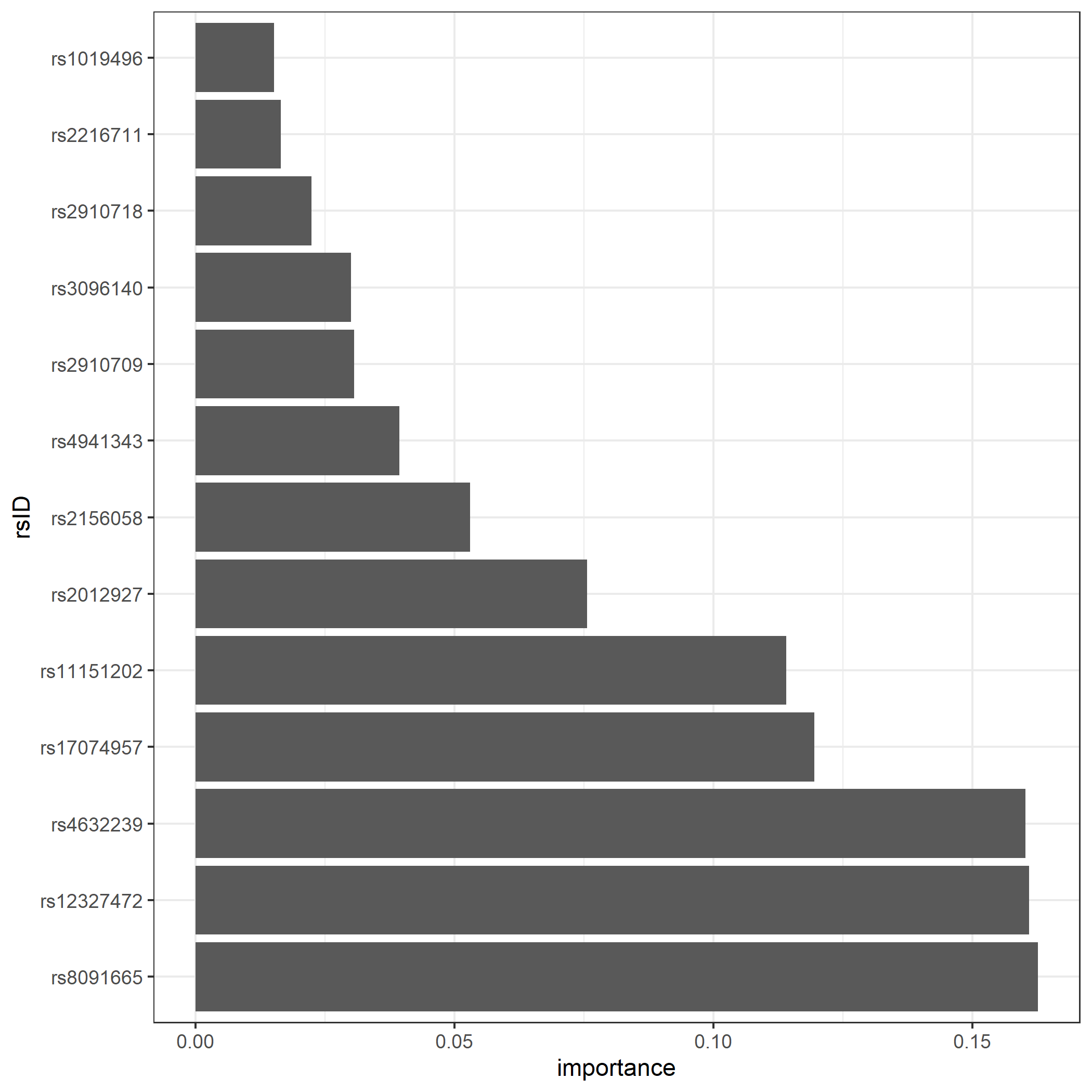


**Figure S10**: Relative impact of SNPs on mechanism 9.


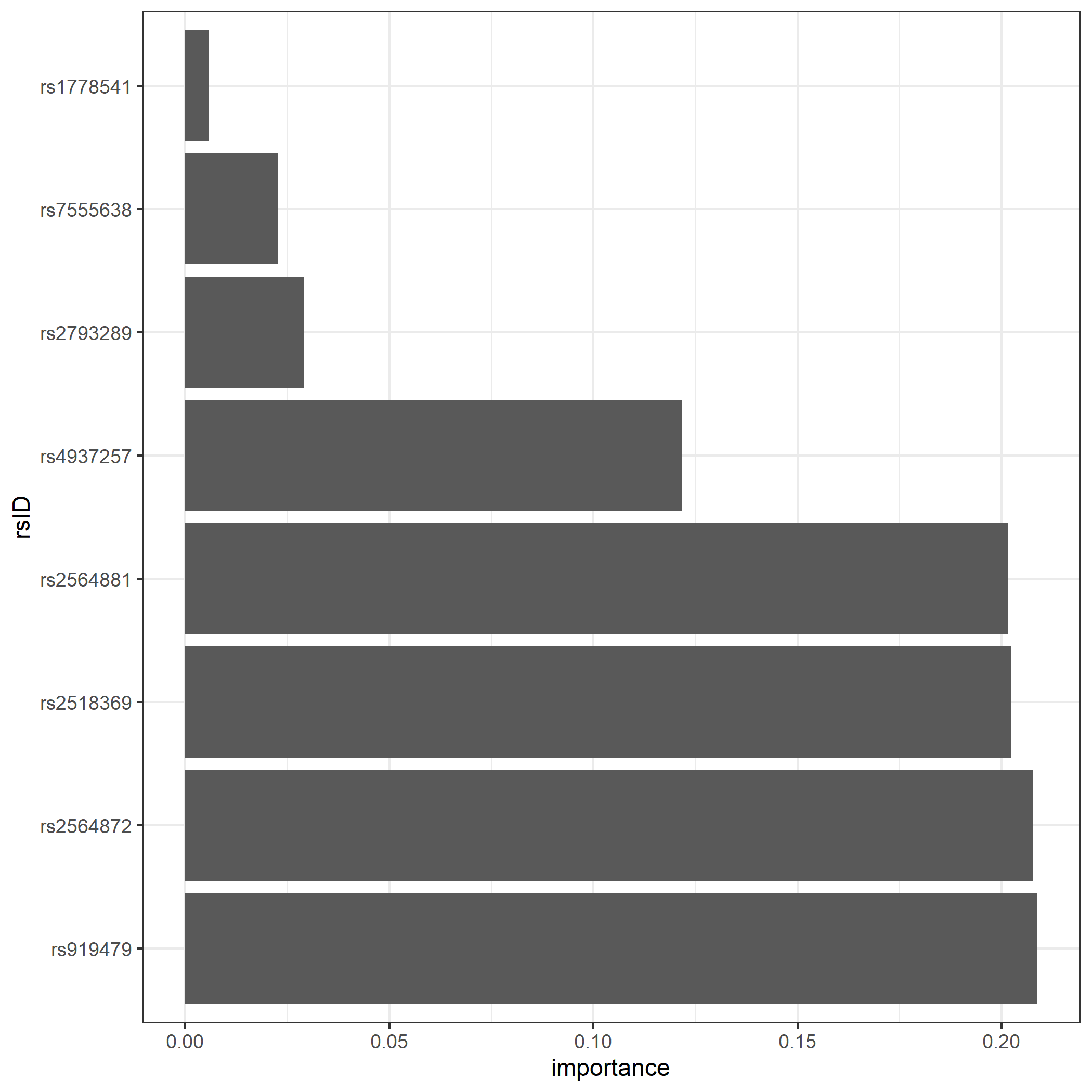


**Figure S11**: Relative impact of SNPs on mechanism 10.


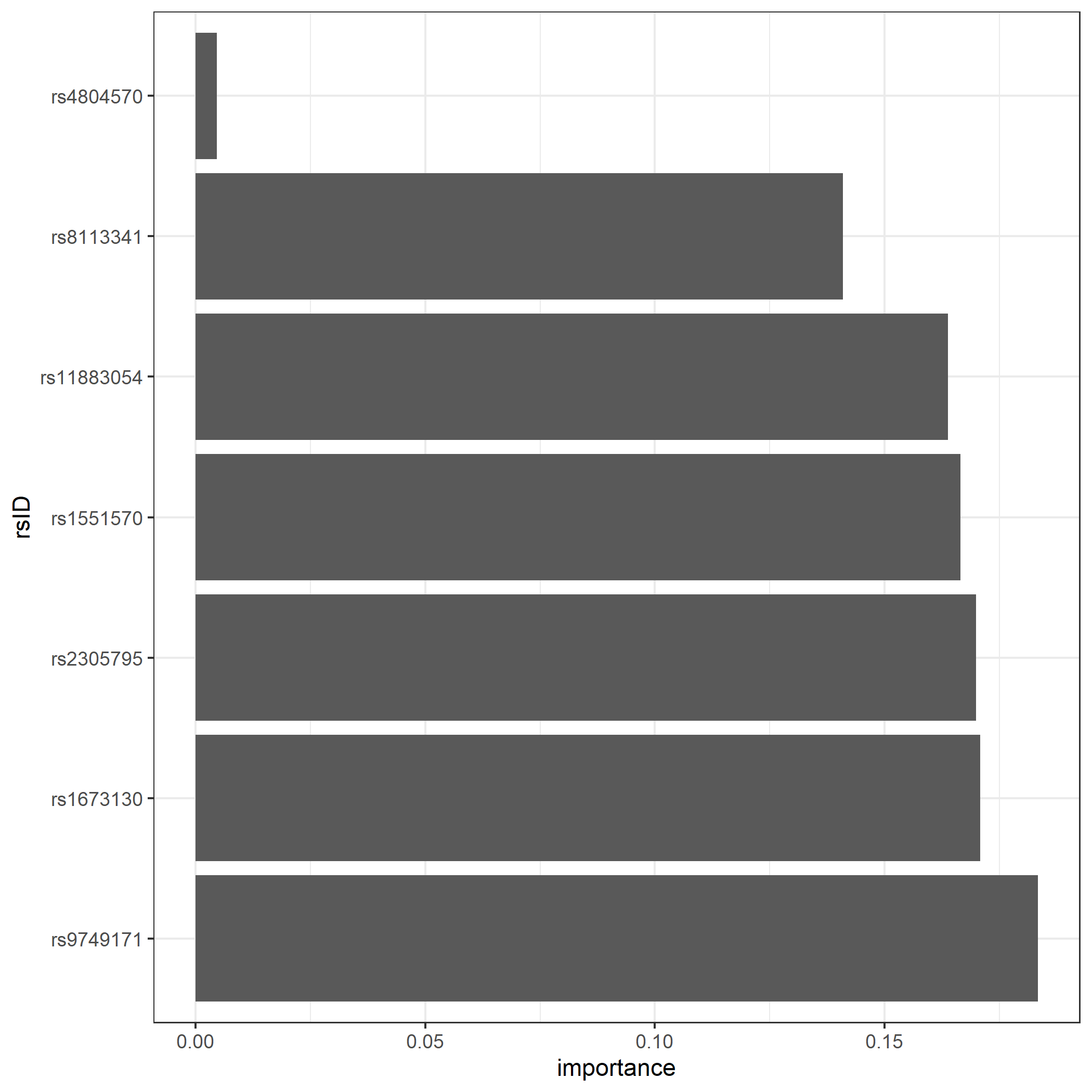


**Figure S12**: Relative impact of SNPs on mechanism 11.


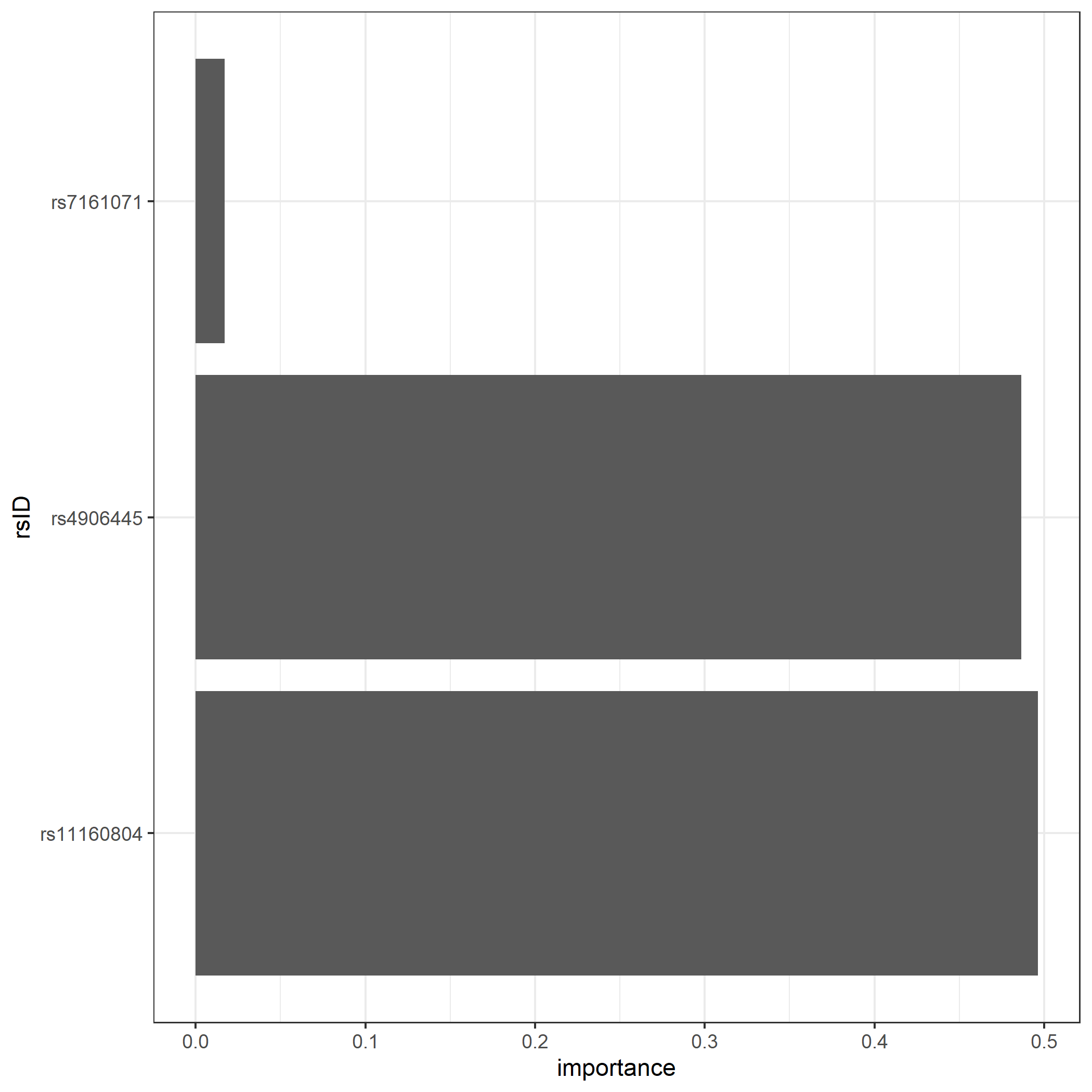


**Figure S13:** Relative impact of SNPs on mechanism 12.


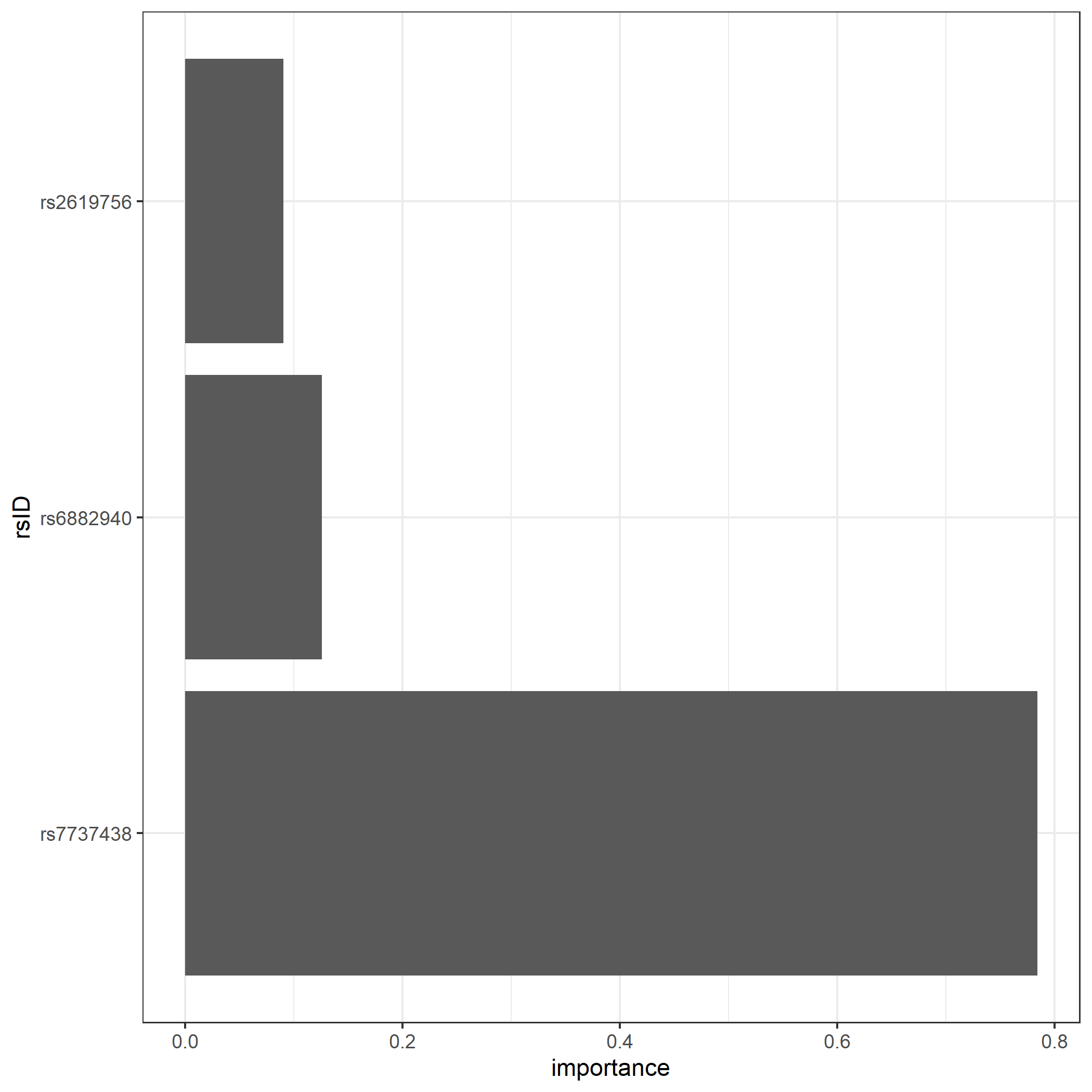


**Figure S14**: Relative impact of SNPs on mechanism 13.


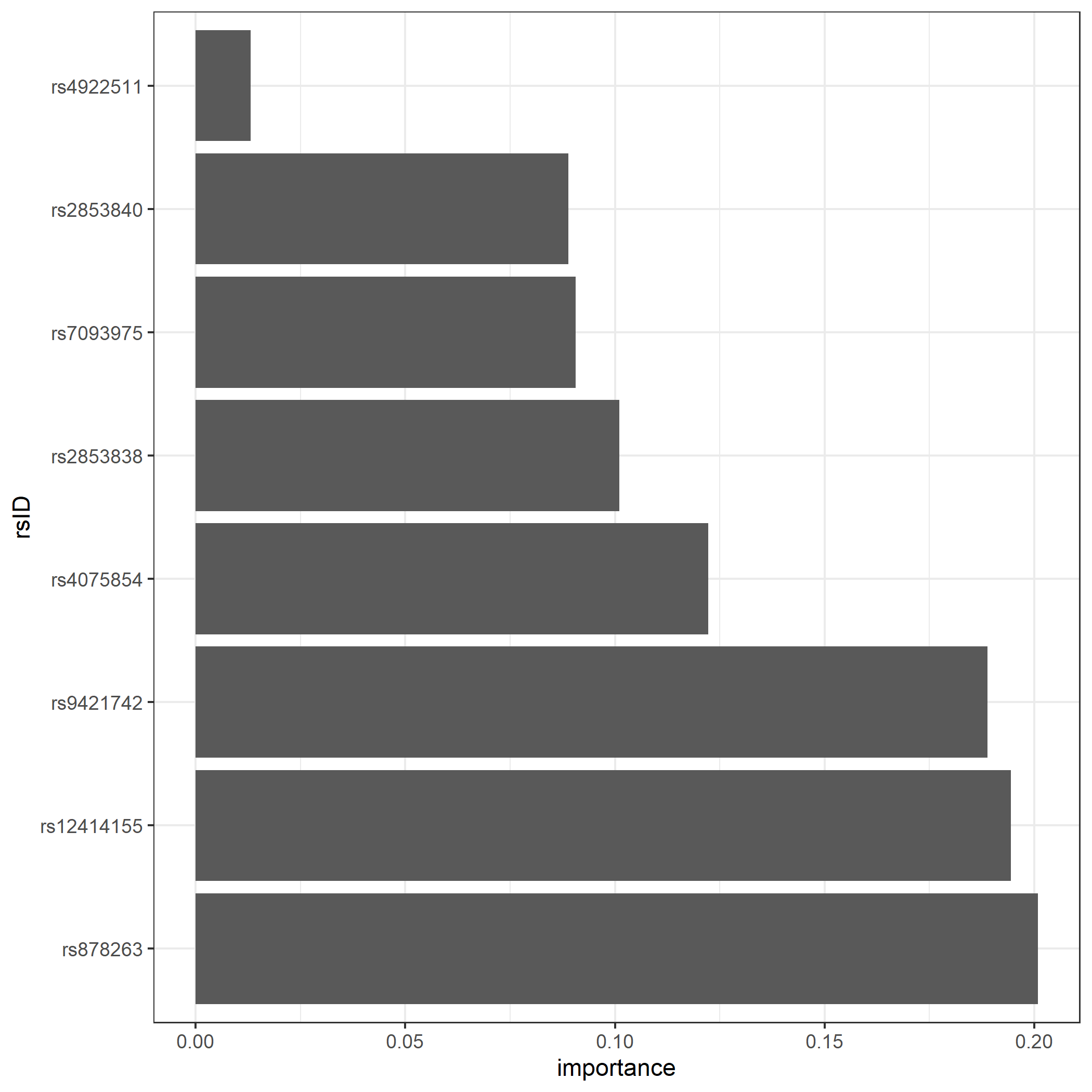


**Figure S15**: Relative impact of SNPs on mechanism 14.


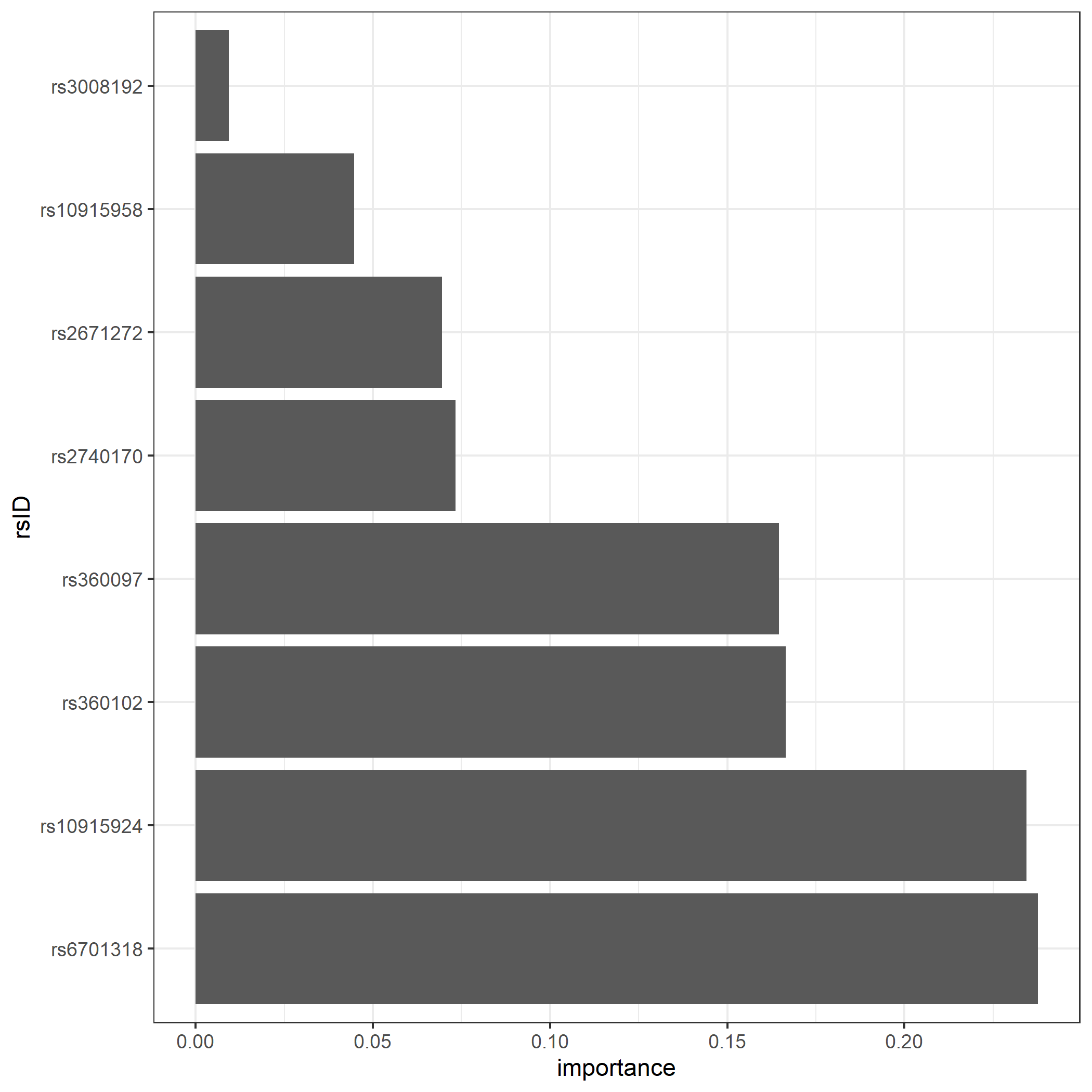


**Figure S16**: Relative impact of SNPs on mechanism 15.

# Consensus sNMF Clustering

We used the consensus sNMF approach by [(Kim and Park, 2007)](https://www.zotero.org/google-docs/?QTMCJT) implemented in R-package NMF [(Gaujoux and Seoighe, 2010)](https://www.zotero.org/google-docs/?BMOgaY). 50 repeats starting from a modified independent component analysis (ICA) based initialization were run [(Benachir et al., 2013)](https://www.zotero.org/google-docs/?wfuhhN), and the resulting consensus matrix clustered via agglomerative hierarchical clustering. Resulting consensus matrices for ADNI, PPMI and a joint ADNI + PPMI dataset for $k=4$clusters are shown in Figures S17 - S19. The choice of $k=4$ clusters was based on the analysis of the proportion of ambiguously clustered pairs (PAC), silhouette index (SI) and cophenetic correlation, as described in the main text (Tables S3 - S5). SI is a standard measure commonly used to validate the consistency within clusters of data. More specifically: The silhouette *s*(*i*) for data point (here: patient) *i* assigned to cluster *C_i_* is defined as


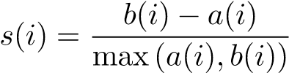


provided that |*C_i_*| *>* 1, and 0 otherwise. Furthermore, *a*(*i*) is the mean distance of data point *i* to all other data points in cluster *C_i_*, and *b*(*i*) is defined as


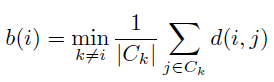


where d(i,j) is the distance between data points i and j. The silhouette s(i) ranges from -1 to 1, where 0 indicates that data point i falls in between two clusters. The silhouette index SI is defined as the mean s(i) over all data points. A value closer to 1 indicates a more tightly grouping of data points into clusters; values close to 0 indicate that samples are on or very close to the decision boundary of the neighboring clusters. Finally, negative values indicate that those samples might be wrongly assigned in the clusters.

We repeated the entire consensus sNMF clustering based on a randomly permuted dataset for 30 times and then estimated the 95% confidence interval of the silhouette index and cophenetic correlation of the randomly permuted data (based on the assumption of approximate normality according to the central limit theorem). The random permutation was done on the raw SNP profile, and subsequently the sparse autoencoding step was run, as described before.

Figures S20, S21 show the coefficient matrix $H$ and basis matrix $W$ obtained from the final joint clustering of ADNI AD and PPMI PD patients. The latter highlights the contribution of specific mechanisms to each of the clusters. According to [(Kim and Park, 2007)](https://www.zotero.org/google-docs/?xyag7l) a score $S{}_{i}$for feature $i$ can be defined as

$$S{}_{i} = 1 + \frac{1}{log{}_{2}k} \sum_{q=1}^{k} p(i,q) log{}_{2} p(i,q)$$

Where $p(i,q)$ is the probability that the i-th feature contributes to basis $q$:

$$p(i,q)=\frac{W(i,q)}{\sum_{r=1}^{k} W(i,r)}$$

The feature scores are real values within the range [0,1]. The higher the feature score the more cluster-specific the corresponding feature.

In agreement with Kim and Park in Table S1 we highlighted those features, for which

1. the feature score is greater than µ+3σ, where µ and σ are the median and the median absolute deviation (MAD) of the feature scores respectively;
2. the maximum contribution to a basis component is greater than the median of all contributions (i.e. of all elements of $W$).


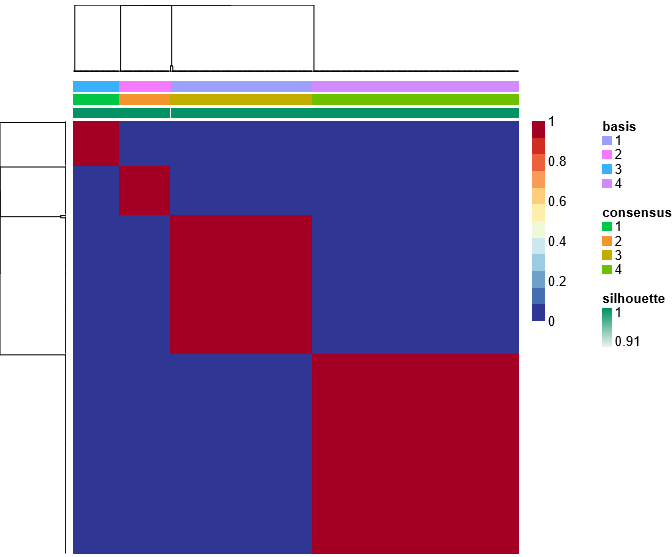


**Figure S17**: Consensus matrix resulting from repeated sNMF clustering of ADNI patients (n = 486)


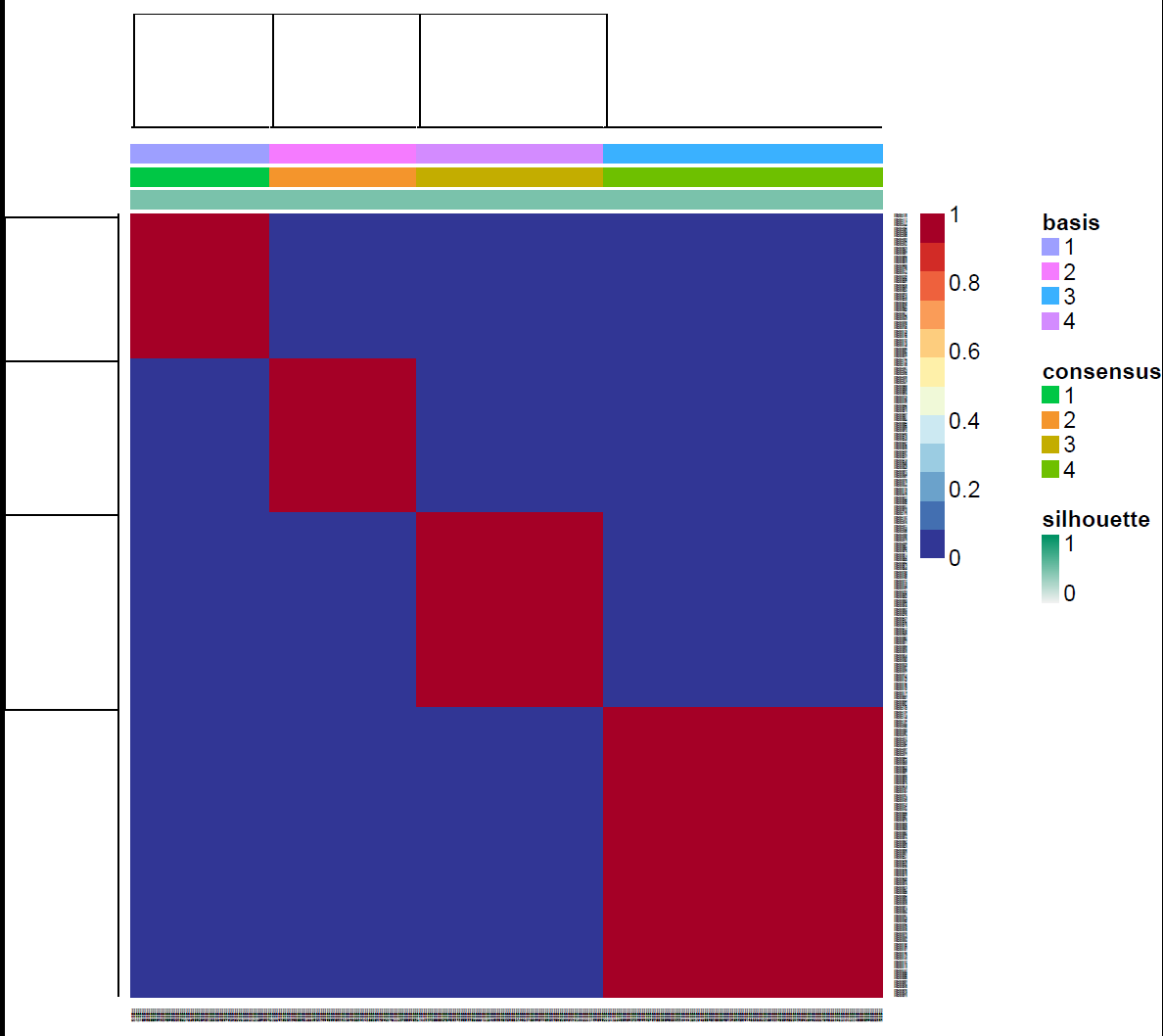


**Figure S18**: Consensus matrix resulting from repeated sNMF clustering of PPMI patients (n = 358)


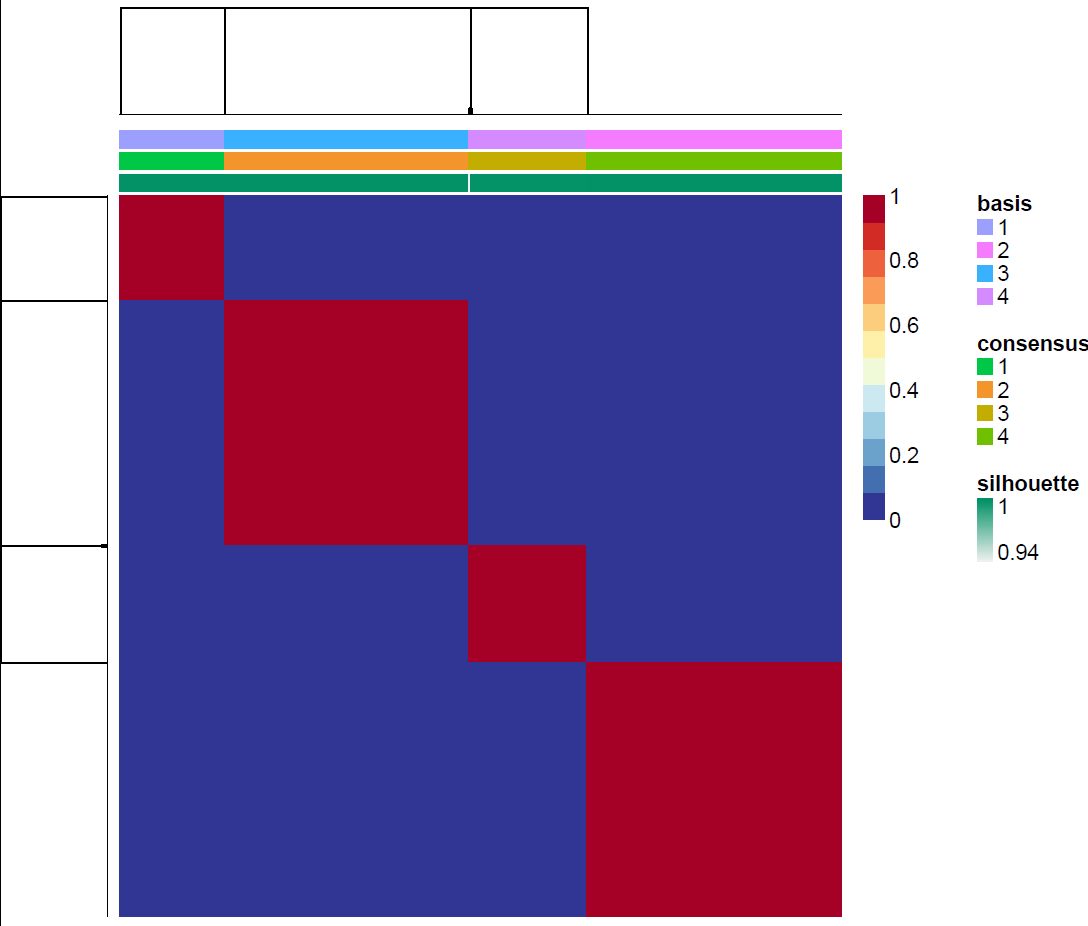


**Figure S19**: Consensus matrix resulting from repeated sNMF clustering of joint ADNI+PPMI patients (n = 844)

**Table S3**: Consensus clustering of ADNI data (n = 486) based on genotype: proportion of ambiguously clustered pairs (PAC), silhouette index and cophenetic correlation. The orange row corresponds to the number of selected clusters.

| **clusters** | **PAC** | **Cophenetic** | **Cophenetic.random.mean** | **CI.cophenetic.random** | **Silhouette** | **Silhouette.random.mean** | **CI.silhouette.random** |
| --- | --- | --- | --- | --- | --- | --- | --- |
| 2 | 0.008 | 0.99984 | 0.99961 | 0.9994; 0.99982 | 0.99783 | 0.99861 | 0.99791; 0.99931 |
| 3 | 0.003 | 0.99982 | 0.99046 | 0.98405; 0.99687 | 0.99907 | 0.9597 | 0.93636; 0.98304 |
| 4 | 0 | 0.99999 | 0.97638 | 0.9672; 0.98556 | 0.99932 | 0.88269 | 0.84571; 0.91967 |
| 5 | 0.003 | 0.99952 | 0.96494 | 0.95185; 0.97803 | 0.99796 | 0.84097 | 0.79243; 0.88951 |
| 6 | 0.152 | 0.97168 | 0.94557 | 0.92733; 0.96381 | 0.83316 | 0.75388 | 0.70059; 0.80717 |
| 7 | 0 | 1 | 0.9184 | 0.89759; 0.93921 | 1 | 0.66462 | 0.604; 0.72524 |
| 8 | 0 | 1 | 0.91409 | 0.88702; 0.94116 | 1 | 0.65546 | 0.5841; 0.72682 |
| 9 | 0.162 | 0.95037 | 0.90153 | 0.87956; 0.9235 | 0.73043 | 0.58483 | 0.52758; 0.64208 |
| 10 | 0.138 | 0.9446 | 0.88305 | 0.85638; 0.90972 | 0.68215 | 0.53971 | 0.47208; 0.60734 |

**Table S4**: Consensus clustering of PPMI data (n = 358) based on genotype: proportion of ambiguously clustered pairs (PAC), silhouette index and cophenetic correlation. The orange row corresponds to the number of selected clusters.

| **clusters** | **PAC** | **Cophenetic** | **Cophenetic.random.mean** | **CI.cophenetic.random** | **Silhouette** | **Silhouette.random.mean** | **CI.silhouette.random** |
| --- | --- | --- | --- | --- | --- | --- | --- |
| 2 | 0.501 | 0.97224 | 0.98071 | 0.96895; 0.99247 | 0.84694 | 0.93947 | 0.91065; 0.96829 |
| 3 | 0.003 | 0.9993 | 0.98869 | 0.98008; 0.9973 | 0.9968 | 0.95374 | 0.92444; 0.98304 |
| 4 | 0 | 1 | 0.98135 | 0.97044; 0.99226 | 1 | 0.91311 | 0.87414; 0.95208 |
| 5 | 0 | 1 | 0.95517 | 0.9384; 0.97194 | 1 | 0.80688 | 0.75227; 0.86149 |
| 6 | 0 | 1 | 0.95365 | 0.93305; 0.97425 | 1 | 0.8007 | 0.73501; 0.86639 |
| 7 | 0 | 0.99832 | 0.94777 | 0.92967; 0.96587 | 0.9591 | 0.74836 | 0.69105; 0.80567 |
| 8 | 0 | 1 | 0.92941 | 0.91251; 0.94631 | 1 | 0.67116 | 0.61671; 0.72561 |
| 9 | 0 | 1 | 0.90935 | 0.88665; 0.93205 | 1 | 0.62315 | 0.55908; 0.68722 |
| 10 | 0 | 1 | 0.89304 | 0.86991; 0.91617 | 1 | 0.56605 | 0.5033; 0.6288 |

**Table S5**: Consensus clustering of joint ADNI + PPMI data (n = 844) based on genotype: proportion of ambiguously clustered pairs (PAC), silhouette index and cophenetic correlation. The orange row corresponds to the number of clusters selected for further downstream analysis according to the criteria explained in the main document.

| **clusters** | **PAC** | **Cophenetic** | **Cophenetic.random.mean** | **CI.cophenetic.random** | **Silhouette** | **Silhouette.random.mean** | **CI.silhouette.random** |
| --- | --- | --- | --- | --- | --- | --- | --- |
| 2 | 0.5 | 0.98147 | 0.97227 | 0.96206; 0.98248 | 0.87903 | 0.90644 | 0.87639; 0.93649 |
| 3 | 0.001 | 0.99997 | 0.98537 | 0.97509; 0.99565 | 0.99944 | 0.94428 | 0.91043; 0.97813 |
| 4 | 0 | 0.99999 | 0.9728 | 0.95895; 0.98665 | 0.99971 | 0.88233 | 0.83519; 0.92947 |
| 5 | 0.001 | 0.99991 | 0.96555 | 0.95417; 0.97693 | 0.99858 | 0.83565 | 0.79201; 0.87929 |
| 6 | 0 | 1 | 0.93395 | 0.9126; 0.9553 | 1 | 0.73794 | 0.67289; 0.80299 |
| 7 | 0 | 1 | 0.94486 | 0.92926; 0.96046 | 1 | 0.75139 | 0.69935; 0.80343 |
| 8 | 0 | 1 | 0.94585 | 0.92795; 0.96375 | 1 | 0.73722 | 0.68234; 0.7921 |
| 9 | 0.001 | 0.99972 | 0.9168 | 0.89922; 0.93438 | 0.99841 | 0.64023 | 0.58561; 0.69485 |
| 10 | 0.123 | 0.9418 | 0.93011 | 0.90956; 0.95066 | 0.663 | 0.68003 | 0.61353; 0.74653 |


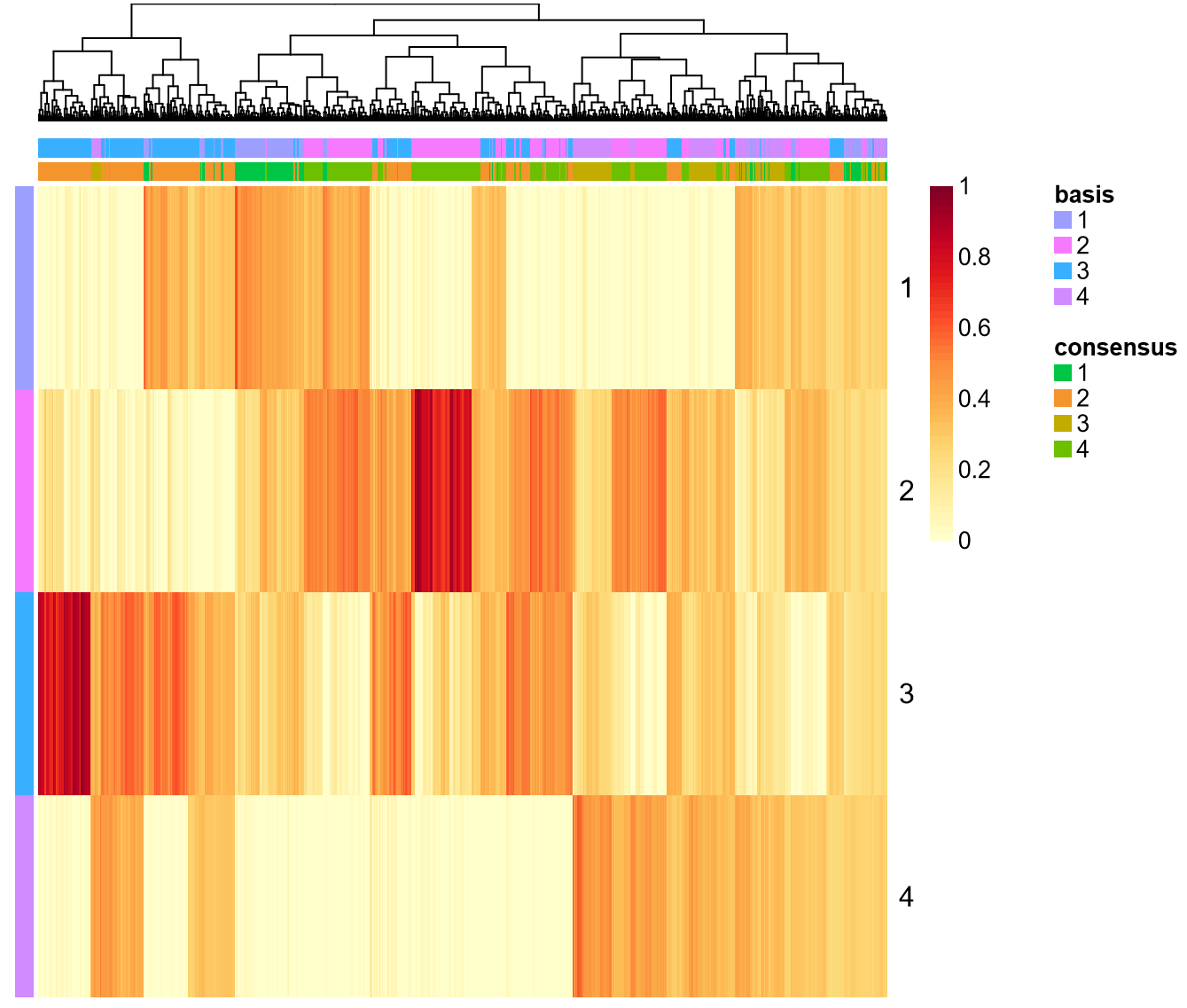


**Figure S20**: Coefficient matrix corresponding to the best among 30 repeats of sNMF clustering of joint ADNI + PPMI patients. A darker color visualizes stronger confidence of a patient falling into the cluster indicated in each row. The result of the consensus clustering is shown with an additional color bar.


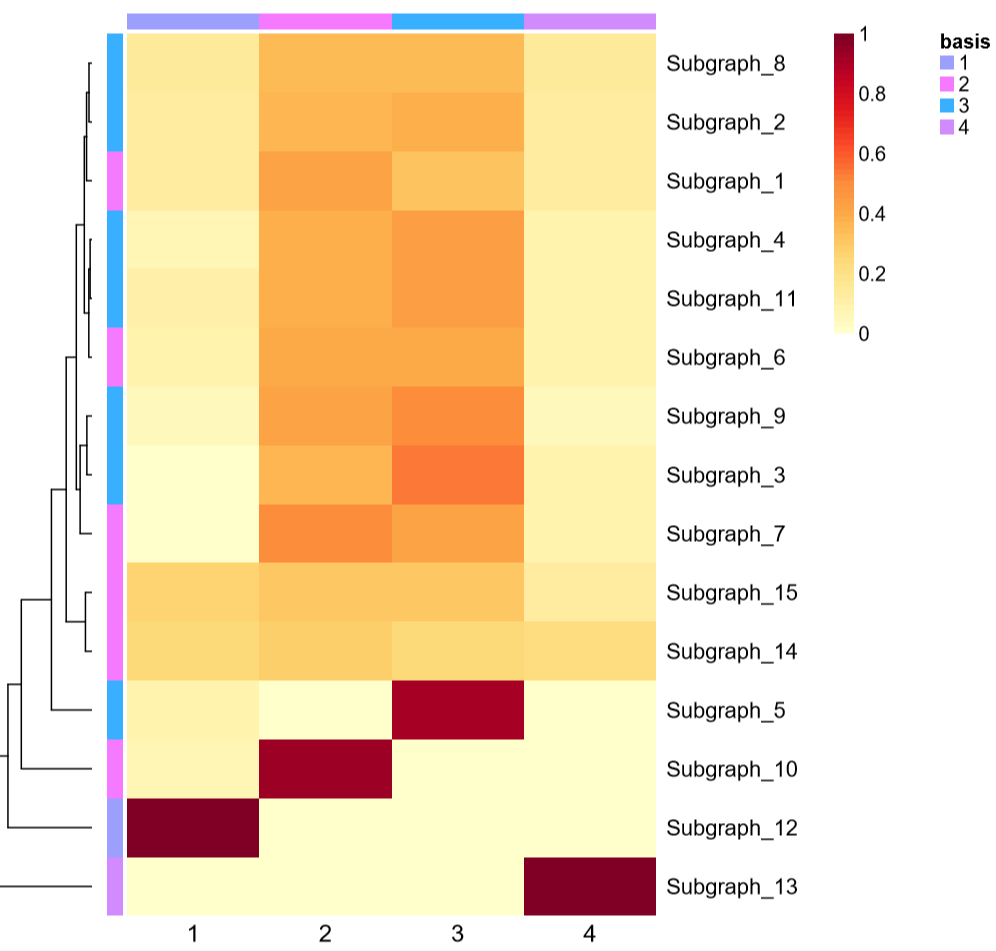


**Figure S21**: Basis matrix corresponding to the best among 30 repeats of sNMF clustering of joint ADNI + PPMI patients. A darker color visualizes a stronger contribution of a mechanism (= cause-effect relationship subgraph) to the cluster indicated in each column. Subgraphs are numbered in agreement to the “Feature ID” in Table 1.


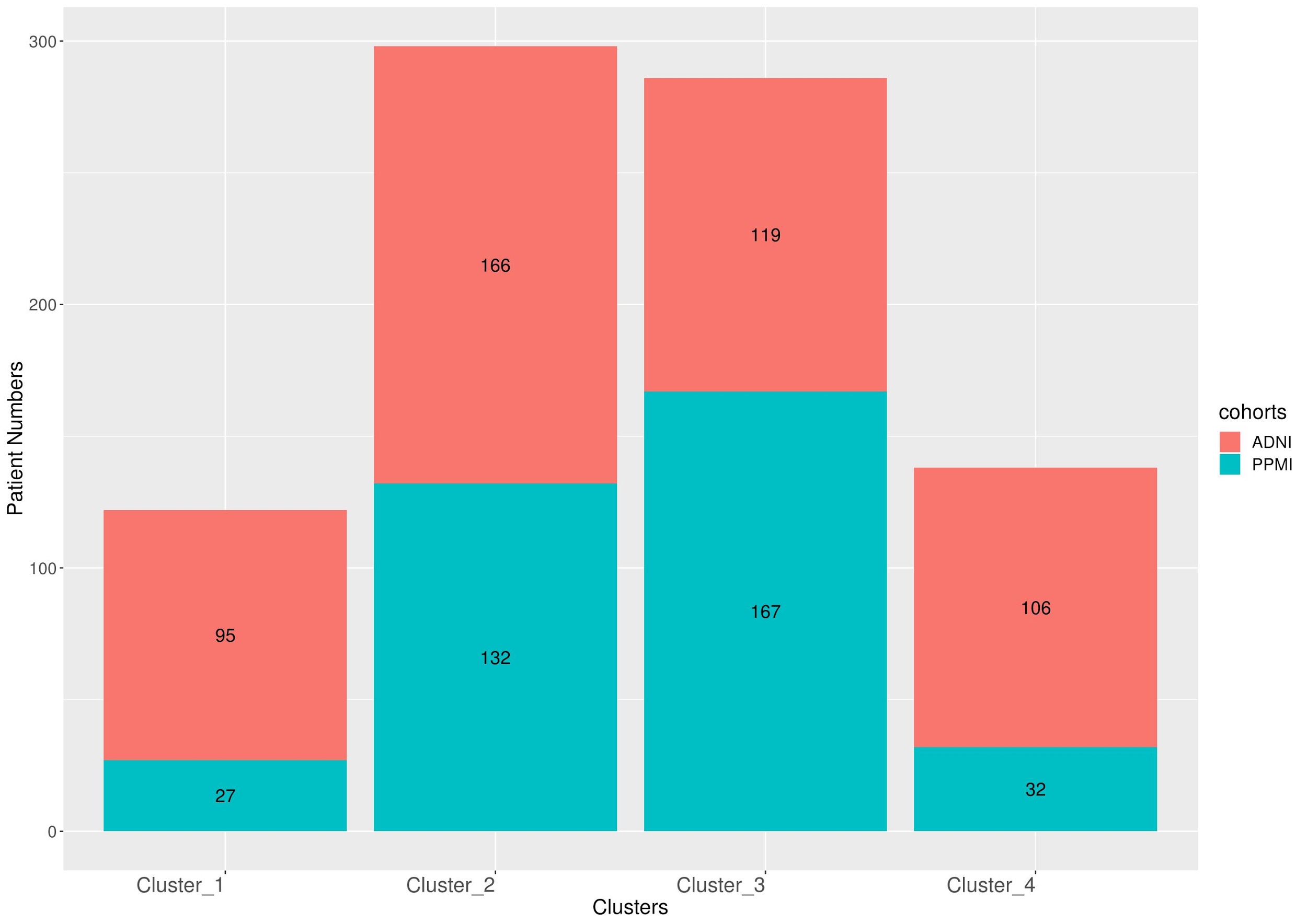


**Figure S22**: Composition of clusters in the joint ADNI + PPMI data.

# Validation of Patient Subtypes in Independent Studies

Consensus sNMF clustering of patients in the merged integrated AETIONOMY AD and PD datasets was run using the same methodology that is described for the discovery phase. Our defined criteria for selecting an optimal number of clusters suggest 4 clusters in the merged validation cohort (Tables S6), and these clusters showed a mixture of AD and PD patients (Figure S23).

As described in the main document we next developed a supervised machine learning classifier for assigning patients in our validation studies to clusters established on the basis of the discovery cohorts (ADNI + PPM). That means we trained that lasso classifier based on ADNI + PPMI data and then applied it to further validation data. As explained in the main text the coherence between predicted cluster membership of validation data and existing clusters in the discovery cohorts was assessed via the in-group proportion (IGP) measure [(Kapp and Tibshirani, 2007)](https://www.zotero.org/google-docs/?7n4L7L). Separate IGP assessments for the integrated AETIONOMY AD and PD cohorts are shown in Figures S24, S25.

To assess the prediction accuracy of the lasso classifier we conducted a repeated, nested cross-validation evaluation based on ADNI + PPMI data. Figure 2E in the main document shows a boxplot of the estimated prediction performance of the lasso classifier in terms of the multi-class area under ROC curve (AUC), which is the average of all 6 AUCs for possible pairwise binary separations [(Hand and Till, 2001)](https://www.zotero.org/google-docs/?72hXSp). The prediction performance was estimated via a 10 times repeated 10-fold cross-validation, and within each cross-validation loop hyper-parameters of the lasso classifier were tuned via an inner 10-fold cross-validation loop based on the training data. Furthermore, training and tuning of the autoencoder network was also done within the cross-validation loop.

As an additional validation of the classifier we assessed the agreement between the patient subgroups identified by re-clustering of our validation data with the predicted clusters from our machine learning classifier via a $\chi2$-test, yielding a (weakly) significant result (p = 0.054). This finding demonstrates that patient subgroups can be predicted via machine learning.

**Table S6**: Consensus clustering of merged validation cohort (n = 561) based on genotype: proportion of ambiguously clustered pairs (PAC), silhouette index and cophenetic correlation. The orange row corresponds to the number of clusters selected for further downstream analysis according to the criteria explained in the main document.

| **clusters** | **PAC** | **Cophenetic** | **Cophenetic.random** | **CI.cophenetic.random** | **Silhouette** | **Silhouette.random** | **CI.silhouette.random** |
| --- | --- | --- | --- | --- | --- | --- | --- |
| 2 | 0.007 | 0.99912 | 0.99151 | 0.98409; 0.99893 | 0.99754 | 0.97496 | 0.95305; 0.99687 |
| 3 | 0.403 | 0.99465 | 0.98606 | 0.97809; 0.99403 | 0.94249 | 0.93581 | 0.90668; 0.96494 |
| 4 | 0 | 1 | 0.98144 | 0.96891; 0.99397 | 1 | 0.92232 | 0.87804; 0.9666 |
| 5 | 0 | 0.99999 | 0.96094 | 0.94253; 0.97935 | 0.99969 | 0.83137 | 0.77391; 0.88883 |
| 6 | 0 | 1 | 0.9623 | 0.94539; 0.97921 | 1 | 0.82205 | 0.77004; 0.87406 |
| 7 | 0 | 0.99999 | 0.94265 | 0.92427; 0.96103 | 0.99938 | 0.74131 | 0.6818; 0.80082 |
| 8 | 0.139 | 0.99683 | 0.92457 | 0.90859; 0.94055 | 0.94109 | 0.66282 | 0.61399; 0.71165 |
| 9 | 0.2 | 0.95326 | 0.92323 | 0.90417; 0.94229 | 0.69535 | 0.65617 | 0.59981; 0.71253 |
| 10 | 0.117 | 0.99216 | 0.91505 | 0.8936; 0.9365 | 0.88991 | 0.63121 | 0.56357; 0.69885 |

**
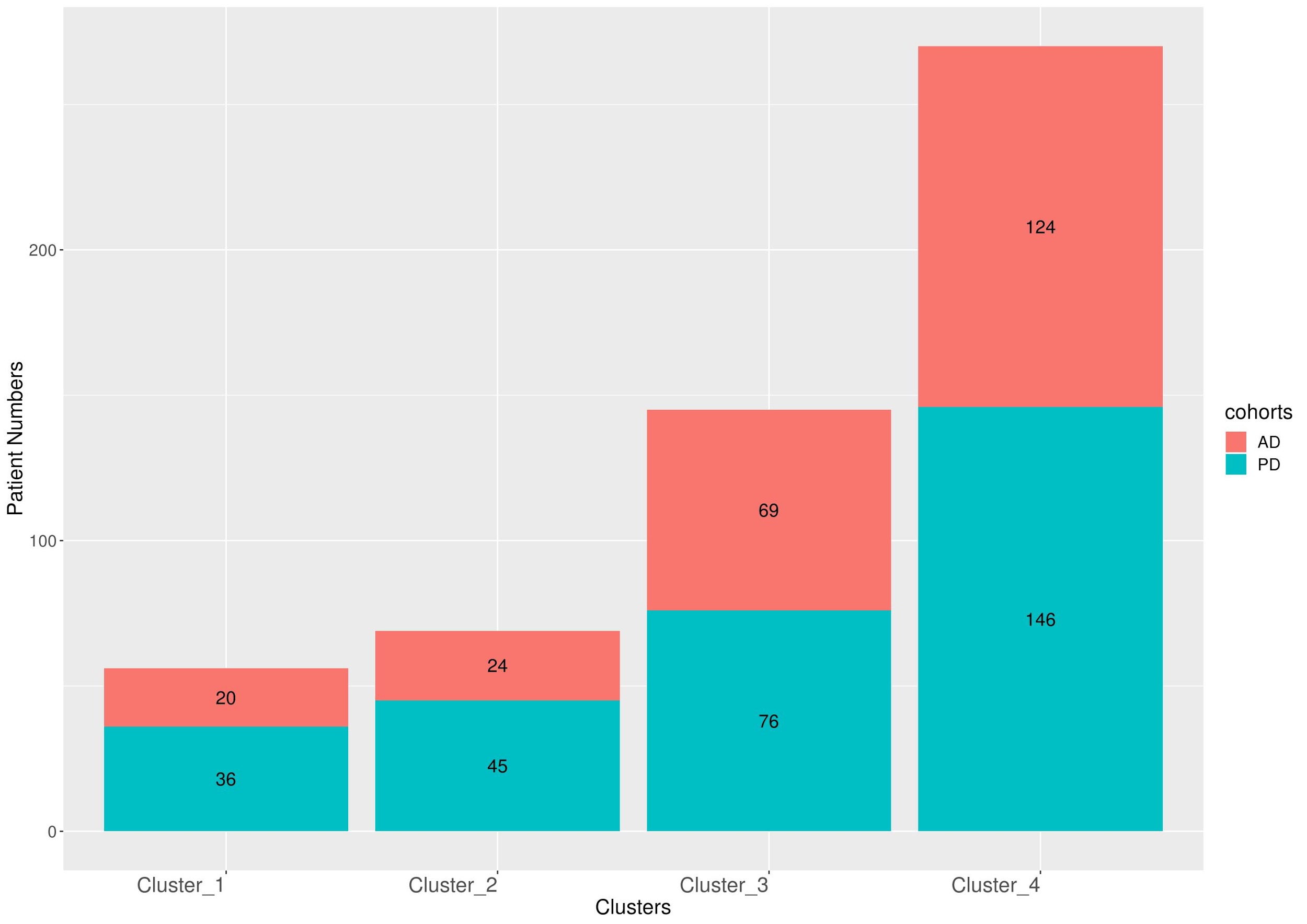
**

**Figure S23**: Composition of clusters in the joint validation cohorts


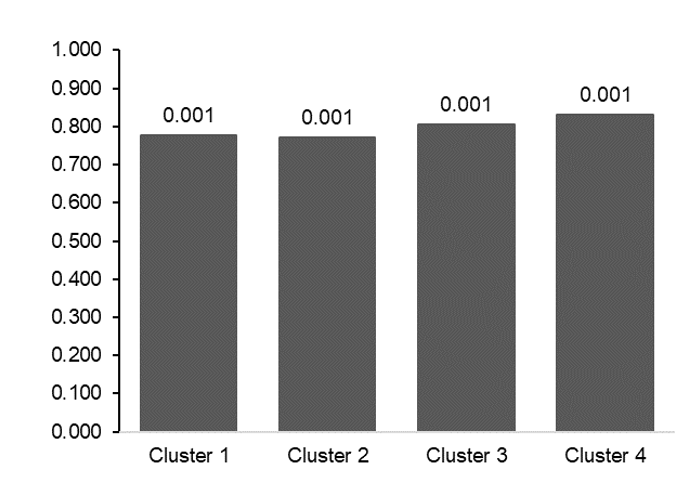


**Figure S24:** In-group proportion (IGP) of patients in integrated AETIONOMY AD cohort after predicting membership to the most likely cluster in the discovery cohort. The p-value of the IGP is shown on top of each bar.


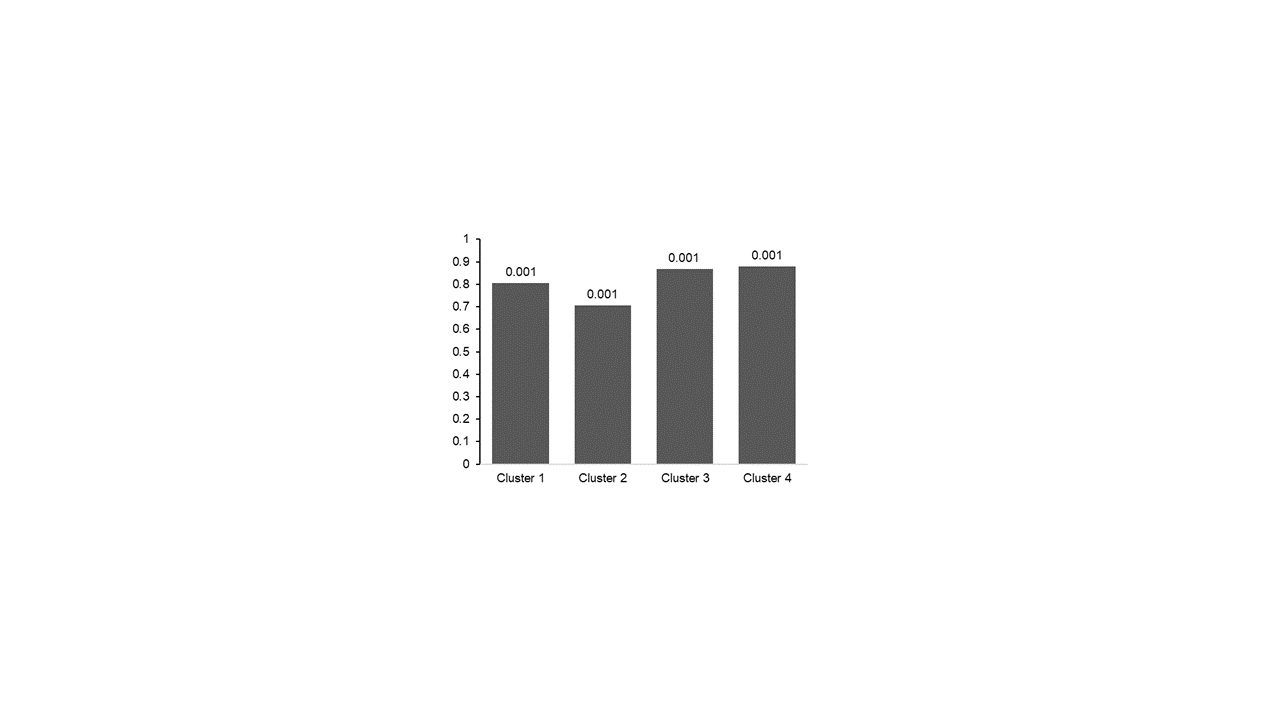


**Figure S25:** In-group proportion (IGP) of patients in integrated AETIONOMY PD cohort after predicting membership to the most likely cluster in the discovery cohort. The p-value of the IGP is shown on top of each bar.

# Statistical Analysis of Clusters

## Clinical Outcome Measures (Baseline)

## We have summarized important demographic and clinical variables of the studied samples from both AD (ADNI, ROSMAP, IDIBAPS) and PD (PPMI, AETIONOMY PD, DIGPD, ICEBERG) cohorts categorized by each of the four clusters below in Tables S7 and S8.

**Table S7:** Demographic and clinical variable distributions of AD cohorts per cluster [mean (SD) / median (IQR)].

| **Cohort** | **Variable** | **Cluster_1** | **Cluster_2** | **Cluster_3** | **Cluster_4** |
| --- | --- | --- | --- | --- | --- |
| **ADNI** | **Age** | 74.01 (7.96) | 74.4 (10.8) | 75.53 (7.37) | 76.85 (9.05) |
|  |  | 74.06 (7.16) | 73.9 (10.15) | 75.41 (6.54) | 75.95 (8.95) |
|  | **Gender (m/f)** | 52/43 | 104/62 | 73/46 | 59/47 |
|  | **Education** | 15.35 (3.43) | 16 (5) | 15.58 (2.93) | 16 (4) |
|  |  | 15.29 (2.62) | 16 (5) | 15.19 (2.97) | 16 (4.75) |
|  | **MMSE** | 23.59 (2.9) | 24 (3.75) | 23.44 (2.84) | 24 (4) |
|  |  | 23.45 (2.69) | 24 (4) | 23.46 (2.58) | 24 (3) |
|  | **CDRSB** | 4.36 (1.58) | 4.5 (2.5) | 4.43 (1.8) | 4.5 (1.5) |
|  |  | 4.35 (1.61) | 4 (1.5) | 4.11 (1.75) | 4 (2) |
|  | **ADAS11** | 17.36 (6.56) | 16.33 (8.67) | 18.11 (6.62) | 18 (8.17) |
|  |  | 17.55 (5.77) | 16.67 (7.84) | 17.83 (6.48) | 17 (8.51) |
|  | **ADAS13** | 27.4 (8.42) | 26 (11) | 28.23 (8.29) | 28.33 (9.41) |
|  |  | 27.24 (7.1) | 27.16 (8.59) | 27.86 (7.97) | 27.33 (9.5) |
|  | **ADASQ4** | 8.17 (1.87) | 8 (3) | 8.52 (1.76) | 9 (2) |
|  |  | 8.28 (1.74) | 9 (3) | 8.43 (1.66) | 9 (2) |
|  | **RAVLT Immediate** | 23.98 (8.17) | 24 (10) | 22.92 (8.1) | 23 (10) |
|  |  | 23.71 (6.47) | 23.5 (8) | 23.39 (7.98) | 24 (10) |
|  | **RAVLT Learning** | 2.15 (1.98) | 2 (2) | 1.85 (1.91) | 2 (2) |
|  |  | 2.04 (1.84) | 2 (2) | 1.68 (1.88) | 2(2) |
| **ROSMAP** | **Age** | 85.8 (5.51) | 86.73 (6.6) | 85.96 (4.73) | 87.99 (6.74) |
|  |  | 85.86 (4.94) | 87.35 (6.37) | 86.25 (4.61) | 88.11 (6.04) |
|  | **Gender (m/f)** | 5/13 | 3/16 | 13/42 | 31/71 |
|  | **Education** | 16.22 (3.17) | 16 (3.25) | 15.74 (4.66) | 16 (5) |
|  |  | 16.02 (3.71) | 16 (7) | 16.34 (3.56) | 16 (4) |
|  | **Global Cognition Score** | -0.26 (0.47) | -0.22 (0.68) | -0.38 (0.63) | -0.44 (0.7) |
|  |  | -0.24 (0.66) | -0.05 (1.04) | -0.18 (0.61) | -0.11 (0.73) |
| **IDIBAPS** | **Age** | 66.11 (0.09) | 58.98 (1.64) | 60.65 (10.88) | 63.32 (9.04) |
|  |  | 66.11 (0.13) | 59.66 (5.41) | 61.38 (5.9) | 62.9 (7.32) |
|  | **Gender (m/f)** | 1/1 | 2/3 | 6/8 | 6/16 |
|  | **Education** | NA | 16 (4) | 17 (6) | 8.5 (3.75) |
|  |  | NA | 14.67 (4.16) | 14.83 (6.49) | 9.38 (3.7) |
|  | **MMSE** | 15.5 (8.5) | 19 (2) | 19 (8) | 18 (6) |
|  |  | 15.5 (12.02) | 19 (3.74) | 16.36 (9.68) | 16.81 (7.26) |

**Table S8:** Demographic and clinical variable distributions of PD cohorts per cluster [mean (SD) / median (IQR)].

| **Cohort** | **Variable** | **Cluster 1** | **Cluster 2** | **Cluster 3** | **Cluster 4** |
| --- | --- | --- | --- | --- | --- |
| **PPMI** | **Age** | 61.39 (9.1) | 63.67 (12.17) | 63.06 (9.22) | 63.12 (14.21) |
|  |  | 60.69 (10.31) | 61.42 (15.5) | 61.81 (9.76) | 63.25 (14.23) |
|  | **Gender (m/f)** | 18/9 | 85/47 | 111/56 | 24/8 |
|  | **UPDRS1** | 5.44 (3.23) | 6 (3.5) | 5.22 (3.89) | 5 (5) |
|  |  | 5.36 (4.3) | 5 (5) | 5.84 (3.51) | 6 (3.5) |
|  | **UPDRS2** | 6.74 (4.36) | 6 (7.5) | 5.13 (3.71) | 4 (5) |
|  |  | 5.91 (4.28) | 5 (6) | 5.19 (3.8) | 4 (4.25) |
|  | **UPDRS3** | 21.41 (10.66) | 20 (15.5) | 20.58 (8.25) | 19 (10) |
|  |  | 20.51 (9.17) | 20 (12) | 19 (7.13) | 19 (8.25) |
|  | **MOCA** | 26.93 (2.13) | 27 (3) | 27.42 (2.05) | 28 (3) |
|  |  | 26.94 (2.46) | 28 (4) | 27.28 (2.4) | 28 (3) |
|  | **ESS** | 5.96 (3.16) | 6 (4.5) | 5.88 (3.23) | 6 (5) |
|  |  | 5.52 (3.39) | 5 (5) | 5.09 (2.88) | 4.5 (4) |
|  | **RBD** | 4 (2.5) | 3 (3) | 4.16 (2.76) | 3 (4) |
|  |  | 3.89 (2.58) | 3 (3) | 4.44 (2.71) | 4 (3.25) |
| **AETIONOMY PD** | **Age** | 64 (7.58) | 64 (8) | 64.7 (6.45) | 65.5 (9.75) |
|  |  | 59.83 (10.33) | 62 (16.25) | 63.07 (8.13) | 64 (10) |
|  | **Gender (m/f)** | 8/3 | 7/3 | 17/7 | 27/16 |
|  | **MDS-UPDRS 1** | 5.27 (3.13) | 4 (3) | 10.6 (4.03) | 10.5 (7.25) |
|  |  | 8.42 (5.35) | 9 (8.25) | 6.59 (4.26) | 7 (6) |
|  | **MDS-UPDRS 2** | 8.18 (4.77) | 7 (6.5) | 9.4 (3.95) | 10 (5.75) |
|  |  | 7.75 (5.52) | 7 (8.5) | 7.24 (4.89) | 7 (8) |
|  | **MDS-UPDRS3 off** | 40.29 (13.8) | 38 (22.5) | 27.33 (11.68) | 31 (16) |
|  |  | 27.06 (14.54) | 28 (20) | 26.53 (11.55) | 28 (16.75) |
|  | **MDS-UPDRS3 on** | 32.27 (11.63) | 30 (13) | 25.17 (11.53) | 30 (13.5) |
|  |  | 26.53 (16.52) | 23 (15) | 21.97 (9.37) | 24 (9) |
|  | **Total MMSE** | 27.73 (1.95) | 28 (3.5) | 29 (1.15) | 29 (1) |
|  |  | 28.32 (1.91) | 28.5 (2) | 28.79 (1.57) | 29 (2) |
|  | **Total MOCA** | 25 (3.22) | 26 (4) | 27.4 (2.37) | 28 (2.25) |
|  |  | 26.09 (3.7) | 27 (4.75) | 26.69 (2.77) | 27 (3) |
|  | **Schwab-England score** | 2.45 (0.69) | 2 (1) | 2.1 (0.32) | 2 (0) |
|  |  | 2.12 (0.54) | 2 (0) | 1.83 (0.7) | 2 (1) |
|  | **HADS anxiety** | 4.18 (2.64) | 5 (3.5) | 4.9 (3.93) | 6.5 (7) |
|  |  | 6.29 (4.12) | 5.5 (3.5) | 4.88 (3.89) | 4 (4.75) |
| **DIGPD** | **Age** | 58.32 (11.37) | 61 (15.75) | 62.14 (8.54) | 62 (12) |
|  |  | 60.97 (7.58) | 61 (10.5) | 59.53 (10) | 60 (13.5) |
|  | **Gender (m/f)** | 14/8 | 17/12 | 19/20 | 50/33 |
|  | **MDS-UPDRS 1** | 7.45 (5.41) | 6 (6) | 6.97 (3.84) | 6 (5) |
|  |  | 7.26 (4.87) | 7 (8) | 6.89 (4.13) | 7 (6) |
|  | **MDS-UPDRS 2** | 5.64 (3.23) | 6 (3.75) | 6.62 (3.12) | 6 (5) |
|  |  | 5.87 (4.3) | 6 (6.5) | 5.96 (3.71) | 5 (5) |
|  | **MDS-UPDRS3 off** | 16.25 (6.18) | 16 (5.25) | 32 (NA) | 32 (0) |
|  |  | 21.67 (9.07) | 23 (9) | 19.4 (7.64) | 21 (9) |
|  | **MDS-UPDRS3 on** | 16.88 (7.67) | 19.5 (14.25) | 18.96 (9.35) | 19 (11) |
|  |  | 16.78 (7.17) | 19 (10.25) | 15.92 (8.03) | 14 (11.25) |
|  | **Total MMSE** | 28.73 (1.78) | 29 (1.75) | 27.86 (2.09) | 28 (2.25) |
|  |  | 28.74 (1.55) | 29 (2) | 28.14 (2.22) | 29 (3) |
|  | **Total MOCA** | NA | NA | NA | NA |
|  |  | NA | NA | NA | NA |
|  | **Schwab-England score** | 1.64 (0.49) | 2 (1) | 1.93 (0.65) | 2 (0) |
|  |  | 1.76 (0.71) | 2 (1) | 1.66 (0.65) | 2 (1) |
|  | **HADS anxiety** | 7.45 (4.25) | 6.5 (3.75) | 7.59 (3.64) | 7 (5) |
|  |  | 6.49 (3.82) | 6 (5) | 6.99 (3.58) | 6 (4) |
| **ICEBERG** | **Age** | 66 (14.11) | 68 (14) | 54.17 (12.66) | 52.5 (21.75) |
|  |  | 59.23 (11.11) | 60 (17) | 64.6 (9.05) | 68 (12.75) |
|  | **Gender (m/f)** | 1/2 | 5/1 | 8/5 | 16/4 |
|  | **MDS-UPDRS 1** | 12.33 (4.04) | 13 (4) | 9.33 (3.83) | 9 (3.75) |
|  |  | 9.31 (2.1) | 9 (3) | 9.9 (3.67) | 10.5 (5.25) |
|  | **MDS-UPDRS 2** | 7.33 (2.52) | 7 (2.5) | 8.5 (3.62) | 8 (4.25) |
|  |  | 7 (4.36) | 6 (7) | 8.3 (3.88) | 7.5 (6.25) |
|  | **MDS-UPDRS3 off** | 30 (7.55) | 31 (7.5) | 26.67 (10.8) | 27 (18.25) |
|  |  | 27.85 (5.27) | 28 (9) | 31.45 (7.92) | 30 (11.25) |
|  | **MDS-UPDRS3 on** | 27.33 (7.37) | 30 (7) | 19 (8.66) | 14 (7.5) |
|  |  | 24.22 (4.35) | 24 (5) | 28.74 (8.34) | 27 (11.5) |
|  | **Total MMSE** | 29.33 (1.15) | 30 (1) | 29 (1.26) | 29.5 (1.75) |
|  |  | 29 (1) | 29 (2) | 28.75 (1.07) | 29 (1.25) |
|  | **Total MOCA** | 27 (3.61) | 28 (3.5) | 27 (1.55) | 26 (2.25) |
|  |  | 27.31 (1.75) | 27 (3) | 27.25 (2.15) | 27 (4) |
|  | **Schwab-England score** | 2 (0) | 2 (0) | 1.83 (0.41) | 2 (0) |
|  |  | 2 (0) | 2 (0) | 1.95 (0.39) | 2 (0) |
|  | **HADS anxiety** | 9 (1.73) | 8 (1.5) | 5.33 (2.42) | 6 (3.5) |
|  |  | 5.85 (2.44) | 7 (2) | 8.05 (3.78) | 7.5 (4) |

Based on the methodology explained in the main text, we compared major clinical outcome variables across the 4 established clusters in PD patients (Tables S7 - S9). Analysis of clinical outcome variables in ADNI did not reveal any significant differences across clusters below a false discovery rate threshold of 5% and are therefore not shown.

**Table S9**: Comparison of clinical outcome variables in idiopathic de novo PD patients (n = 42) at baseline in combined ICEBERG study between disease subtypes. *Results have been corrected for the confounding effects of gender, age at PD diagnosis, time passed since diagnosis, and L-DOPA treatment.* P-values have been adjusted for multiple testing via Benjamini and Hochberg’s method. Only variables are shown, which demonstrated nominal significance in a type III ANOVA (Analysis of Deviance).

| **Variable** | **Comparisons** | **Adjusted P-value** |
| --- | --- | --- |
| UPDRS1 | cluster 1 vs 2; cluster 1 vs 3; cluster 1 vs 4; cluster 2 vs 3;  cluster 2 vs 4  cluster 3 vs 4 | **< 0.001**  **< 0.001**  **< 0.001**  **< 0.001**  **< 0.001**  **< 0.001** |
| UPDRS3_on | cluster 1 vs 2; cluster 1 vs 3; cluster 1 vs 4; cluster 2 vs 3;  cluster 2 vs 4  cluster 3 vs 4 | **< 0.001**  **< 0.001**  **< 0.001**  **< 0.001**  **< 0.001**  **< 0.001** |
| Hospital Anxiety and Depression Scale: anxiety | cluster 1 vs 2; cluster 1 vs 3; cluster 1 vs 4; cluster 2 vs 3;  cluster 2 vs 4  cluster 3 vs 4 | **< 0.001**  **< 0.001**  **< 0.001**  **< 0.001**  **< 0.001**  **< 0.001** |

**Table S10**: Comparison of clinical outcome variables in idiopathic de novo PD patients (n = 88) at baseline in combined AETIONOMY PD study between disease subtypes. *Results have been corrected for the confounding effect of ethnicity.* P-values have been corrected for multiple testing via Benjamini and Hochberg’s method. Only variables are shown, which demonstrated nominal significance in a type III ANOVA (Analysis of Deviance).

| **Variable** | **Comparisons** | **Adjusted_P-value** |
| --- | --- | --- |
| UPDRS1 | cluster 1 vs 2; cluster 1 vs 3; cluster 1 vs 4; cluster 2 vs 3;  cluster 2 vs 4  cluster 3 vs 4 | **0.04**  0.13  0.36  0.13  0.36  0.36 |
| Schwab-England scale | cluster 1 vs 2; cluster 1 vs 3; cluster 1 vs 4; cluster 2 vs 3;  cluster 2 vs 4  cluster 3 vs 4 | 0.32  0.27  **0.02**  0.27  **0.02**  **0.02** |

**Table S11**: Comparison of clinical outcome variables in ROSMAP AD patients at baseline (n = 194) between disease subtypes. *The analysis did not reveal any confounding effects.* P-values have been corrected for multiple testing via Benjamini and Hochberg’s method. Only variables are shown, which demonstrated nominal significance in a type III ANOVA (Analysis of Deviance).

| **Variable** | **Comparisons** | **Adjusted_P-value** |
| --- | --- | --- |
| Cognition - working memory domain (average of 3 tests) | cluster 1 vs 2; cluster 1 vs 3; cluster 1 vs 4; cluster 2 vs 3;  cluster 2 vs 4  cluster 3 vs 4 | *0.07*  *0.07*  0.28  *0.07*  0.28  0.28 |

## Clinical Outcome Measures (Longitudinal)

**Table S12**: Comparison of clinical outcome variables over time (ADNI) across disease subtypes. P-values have been corrected for multiple testing via Benjamini and Hochberg’s method. Only variables are shown, which demonstrated nominal significance in a type III ANOVA (Analysis of Deviance).

| **Variable** | **Confounders** | **Contrast** | **Adjusted P-Value** |
| --- | --- | --- | --- |
| ADAS11 |  | 1-2  1-3  1-4  2-3  2-4  3-4 | 1  **<0.001**  1  1  1  1 |
| ADAS13 |  | 1-2  1-3  1-4  2-3  2-4  3-4 | **<0.001**  **<0.001**  1  1  **<0.001**  1 |
| CDRSB | Baseline diagnosis, education, marriage status | 1-2  1-3  1-4  2-3  2.4  3-4 | 1  1  1  1  1  **<0.001** |
| MMSE |  | 1-2  1-3  1-4  2-3  2.4  3-4 | **<0.001**  1  1  1  1  1 |
| RAVLT.immediate |  | 1-2  1-3  1-4  2-3  2.4  3-4 | **<0.001**  1  1  1  1  1 |
| RAVLT.learning |  | 1-2 1-3  1-4  2-3  2.4  3-4 | **<0.001**  **<0.001**  1  1  1  1 |

**Table S13**: Comparison of clinical outcome variables over time (PPMI) across disease subtypes. P-values have been corrected for multiple testing via Benjamini and Hochberg’s method. Only variables are shown, which demonstrated nominal significance in a type III ANOVA (Analysis of Deviance).

| **Variable** | **Confounders** | **Contrast** | **Adjusted P-Value** |
| --- | --- | --- | --- |
| UPDRS3 |  | 1-2  1-3  1-4  2-3  2-4  3-4 | 1  1  1  **<0.001**  1  1 |

## Brain Imaging

Table S14 compares subcortical brain volumes (as calculated by us) of AD patients at ADNI study baseline. Table S15 shows a comparison of DaTSCAN of PPMI patients for different brain regions.

**Table S14**: Significant results in comparisons of 193 subcortical brain volumes in AD diagnosed patients in ADNI between disease subtypes at baseline (n = 206) after correction for confounding effects of age and sex. P-values have been corrected for multiple testing. Only variables are shown, which demonstrated nominal significance in a type III ANOVA (Analysis of Deviance).

| **Variable** | **Comparison** | **Adjusted P-Value** |
| --- | --- | --- |
| **Left calcarine sulcus** | 4 - 1 | **0.027** |
|  | 4 - 2 | **0.027** |
|  | 4 - 3 | **0.027** |
| **Left cuneus gyrus** | 4 - 1 | **0.027** |
|  | 4 - 2 | **0.027** |
|  | 4 - 3 | **0.027** |
| **Right medial occipitotemporal gyrus** | 2 - 1 | **0.027** |
| **Right medial occipitotemporal sulcus** | 2 - 1 | **0.007** |

**Table S15**: Comparison of DaTSCAN brain imaging in de novo PD patients at baseline in PPMI study between disease subtypes. Only variables are shown, which demonstrated nominal significance in a type III ANOVA (Analysis of Deviance). The analysis did not reveal any confounding effects.

| **Variable** | **Comparisons** | **Adjusted P-value** |
| --- | --- | --- |
| Caudate elft | cluster 1 vs 2; cluster 1 vs 3; cluster 1 vs 4; cluster 2 vs 3;  cluster 2 vs 4  cluster 3 vs 4 | *0.94*  *0.36*  *0.09*  *0.36*  *0.09*  *0.09* |
| Putamen left | cluster 1 vs 2; cluster 1 vs 3; cluster 1 vs 4; cluster 2 vs 3;  cluster 2 vs 4  cluster 3 vs 4 | **0.04**  *0.09*  *0.78*  *0.09*  *0.78*  0.78 |
| Count Density Ratio: Caudate / Putamen | cluster 1 vs 2; cluster 1 vs 3; cluster 1 vs 4; cluster 2 vs 3;  cluster 2 vs 4  cluster 3 vs 4 | **0.03**  **0.03**  *0.36*  **0.03**  *0.36*  *0.36* |
| Count Density Ratio (CL):  Caudate contralateral / Putamen contralateral | cluster 1 vs 2; cluster 1 vs 3; cluster 1 vs 4; cluster 2 vs 3;  cluster 2 vs 4  cluster 3 vs 4 | **0.02**  **0.03**  *0.09*  **0.02**  *0.09*  *0.09* |

## CSF Biomarkers

**Table S16**: Comparison of CSF biomarkers in de novo PD patients at baseline in PPMI study between disease subtypes. Only variables are shown, which demonstrated nominal significance in a type III ANOVA (Analysis of Deviance). The analysis did not reveal any confounding effects.

| **Variable** | **Comparisons** | **Adjusted P-value** |
| --- | --- | --- |
| Abeta42 | cluster 1 vs 2; cluster 1 vs 3; cluster 1 vs 4; cluster 2 vs 3;  cluster 2 vs 4  cluster 3 vs 4 | **0.02**  **<0.001**  **<0.001**  **<0.001**  **<0.001**  **<0.001** |

## Genome-wide Transcriptome Analysis

Pre processed RNA-Seq raw read counts for the ROSMAP studies were downloaded from the rnaSeqReProcessing project available from the AMP-AD Knowledge portal (https://www.synapse.org/#!Synapse:syn9702085 - syn9702085): Briefly the RNA was extracted from 724 gray matter dorsolateral prefrontal cortex samples and of these 582 had a RIN score of >5 and were sent for sequencing [(Mostafavi et al., 2018)](https://www.zotero.org/google-docs/?6Cw2ER). Paired end 101 bp sequencing at 50M coverage was conducted on the Illumina HiSeq platform. Following this the rnaSeqReProcessing project (syn9702085) utilised the fastq files and conducted a standardized mapping and counting process. STAR aligner was used to align to the GENCODE24 (GRCh38) human genome on Two pass Mode set as basic. Counts were quantified using the quantMode as Gene Counts from the same STAR programme.

We utilised the raw read counts RNAseq data of 56 AD cases as well as 50 cognitively normal controls that had matching clinical and genotype information in ROSMAP. Based on the previously described consensus sNMF of the integrated AETIONOMY AD + PD cohort we could assign all 56 AD cases to one of the four clusters. A complete breakdown of the number of samples within each of our groups of interest can be seen in the table below:

**Table S17**: Number of AD cases (Codx 4 or 5) and cognitively normal controls (Cogdx = 1) with RNAseq data in ROSMAP.

| **Cluster 1** | **Cluster 2** | **Cluster 3** | **Cluster 4** | **Control** |
| --- | --- | --- | --- | --- |
| 6 | 6 | 14 | 30 | 50 |

RNA-Seq data were TMM normalized via R-package EdgeR (version 3.24.3) [(McCarthy et al., 2012)](https://www.zotero.org/google-docs/?G5P12O). Principal Component Analysis (PCA), hierarchical clustering and boxplots were employed to identify any outlying samples and potential batch effects within this dataset. Additionally, we tested for possible confounding variables (age, gender, study, race, Spanish ethnicity, disease duration and years of education) via a multinomial logistic regression model that was run to compare clinical variables across the groups of interest. None of the potential confounders was found to be significantly correlated with our clusters.

We proceeded with ranking genes with respect to log fold changes via EdgeR. One large model was built containing all samples across all groups and then pairwise differences between clusters as well as to cognitively normal controls were pulled out as contrasts. Due to the low sample size chances to identify differentially expressed genes are low, specifically in consideration of the severe multiple testing issue. We thus abstained from interpreting these results and instead focused on Gene Set Enrichment Analysis (GSEA - [(Subramanian et al., 2005)](https://www.zotero.org/google-docs/?2UMTlL)) using the Generally Applicable Gene Set Enrichment package (GAGE) in R (version 2.32.1) [(Luo et al., 2009)](https://www.zotero.org/google-docs/?x9fm7p). The idea was that in this way it would be possible to a) significantly reduce the multiple testing problem and b) to consider the fact that genes are not expressed independently. We used the most recent Gene Ontology biological process (c5.bp.v6.2.symbols.gmt) / cellular component (c5.cc.v6.2.symbols.gmt) and molecular function (c5.mf.v6.2.symbols.gmt) gene sets, which we downloaded as a gmt file from the Broad Institute website (<http://software.broadinstitute.org/gsea/downloads.jsp>) for GSEA analysis. In addition, KEGG pathways were analyzed [(Kanehisa et al., 2008)](https://www.zotero.org/google-docs/?s7jcno).

Table 19 shows a matrix with the number of significantly enriched GO terms and KEGG pathways in each pairwise comparison after correction for multiple testing via the Benjamini-Hochberg method [(Benjamini and Hochberg, 1995)](https://www.zotero.org/google-docs/?DYGz13).

To get a broader view on relevant biological processes, we visualized gene sets that were solely found as significant in one cluster as enrichment maps [(Merico et al., 2010)](https://www.zotero.org/google-docs/?nh9QX5), see Figures 25 - 28. Such an enrichment map takes the hierarchical relationship between GO terms into consideration and summarizes similar GO terms into clusters, hence facilitating interpretation. Cluster specific KEGG pathways are visualized in Figure 36.

RNA-Seq data from PPMI was processed in the same way as described before for ROSMAP. Table 20 shows the number of cases in each cluster, and Table 21 the number of significant GO terms and KEGG pathways found enriched in each comparison. Cluster specific GO terms are shown in Figures 29 - 31. **There were no GO terms solely found enriched in cluster 1 and hence no Figure is shown.** Cluster specific KEGG pathways are visualized in Figure 41.

Detailed analysis results can be downloaded from <https://clus2bio.scai.fraunhofer.de/>.

**Table S18**: Transcriptome analysis (ROSMAP): number of significant GO terms (biological process) and KEGG pathways (false discovery rate < 5%) in pairwise comparisons between clusters. The diagonal shows the number of significant terms compared to healthy controls.

|  | **Cluster 1** | **Cluster 2** | **Cluster 3** | **Cluster 4** | **vs all other clusters** |
| --- | --- | --- | --- | --- | --- |
| **Cluster 1** | GO-BP: 107; KEGG: 6 | GO-BP: 366; KEGG: 25 | GO-BP: 141; KEGG: 17 | GO-BP: 139; KEGG: 9 | GO-BP: 164; KEGG: 13 |
| **Cluster 2** | - | GO-BP: 336; KEGG: 15 | GO-BP: 0; KEGG: 1 | GO-BP: 62; KEGG: 1 | GO-BP: 51; KEGG: 1 |
| **Cluster 3** | - | - | GO-BP: 299; KEGG: 31 | GO-BP: 0; KEGG: 5 | GO-BP: 0 ; KEGG: 16 |
| **Cluster 4** | - | - | - | GO-BP: 468 ; KEGG: 27 | GO-BP: 76; KEGG: 4 |

**Table S19**: Number of PD cases and healthy normal controls with RNAseq data in PPMI.

| **Cluster 1** | **Cluster 2** | **Cluster 3** | **Cluster 4** | **Control** |
| --- | --- | --- | --- | --- |
| 24 | 146 | 24 | 112 | 151 |

**Table S20**: Transcriptome analysis (PPMI): number of significant GO terms (biological process) and KEGG pathways (false discovery rate < 5%) in pairwise comparisons between clusters. The diagonal shows the number of significant terms compared to healthy controls.

|  | **Cluster 1** | **Cluster 2** | **Cluster 3** | **Cluster 4** | **vs all other clusters** |
| --- | --- | --- | --- | --- | --- |
| **Cluster 1** | GO-BP: 0; KEGG: 1 | GO-BP: 207; KEGG: 3 | GO-BP: 205; KEGG: 12 | GO-BP: 98; KEGG: 0 | GO-BP: 177; KEGG: 4 |
| **Cluster 2** | - | GO-BP: 468; KEGG: 32 | GO-BP: 61; KEGG: 7 | GO-BP: 350; KEGG: 32 | GO-BP: 74; KEGG: 0 |
| **Cluster 3** | - | - | GO-BP: 109; KEGG: 25 | GO-BP: 57; KEGG: 9 | GO-BP: 87; KEGG: 12 |
| **Cluster 4** | - | - | - | GO-BP: 96; KEGG: 1 | GO-BP: 28; KEGG: 1 |


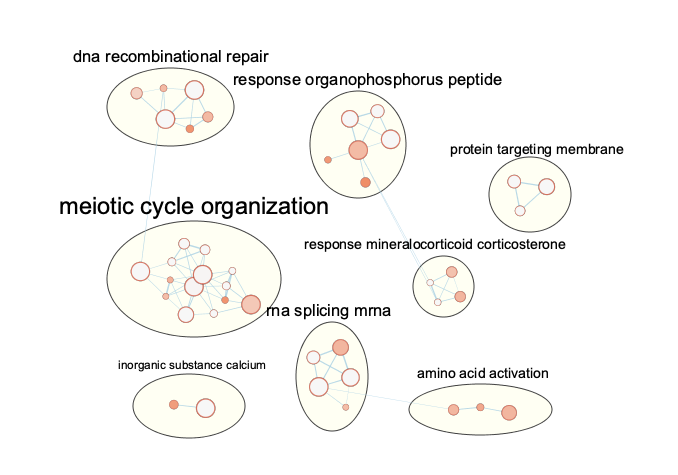


**Figure S26:** Enrichment map of top 10 cluster 1 specific GO terms (biological processes) in AD compared to healthy controls (ROSMAP, gene expression).


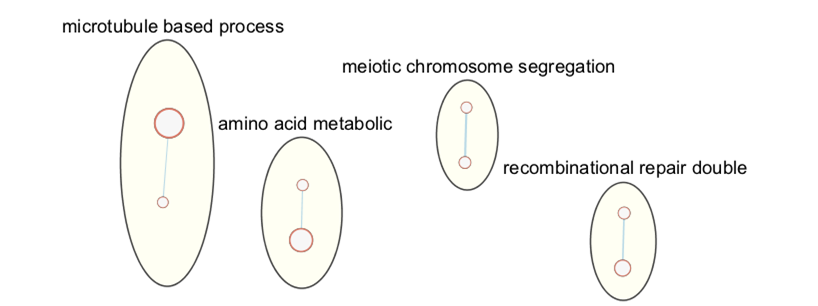


**Figure S27:** Enrichment map of top 10 cluster 2 specific GO terms (biological processes) in AD compared to healthy controls (ROSMAP, gene expression).


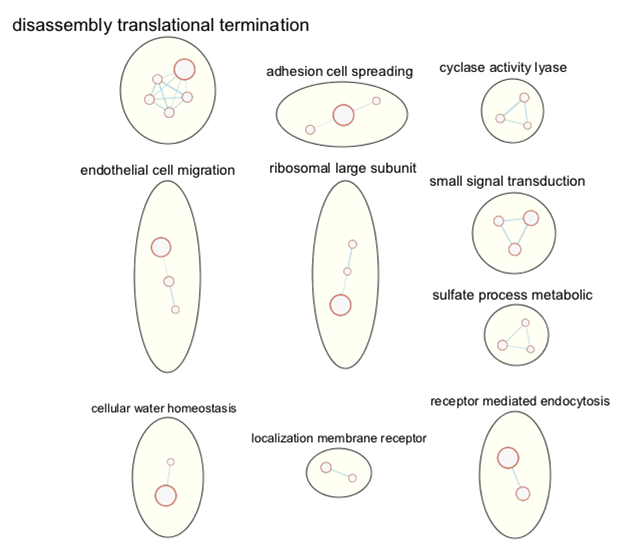


**Figure S28:** Enrichment map of top 10 cluster 3 specific GO terms (biological processes) in AD compared to healthy controls (ROSMAP, gene expression).


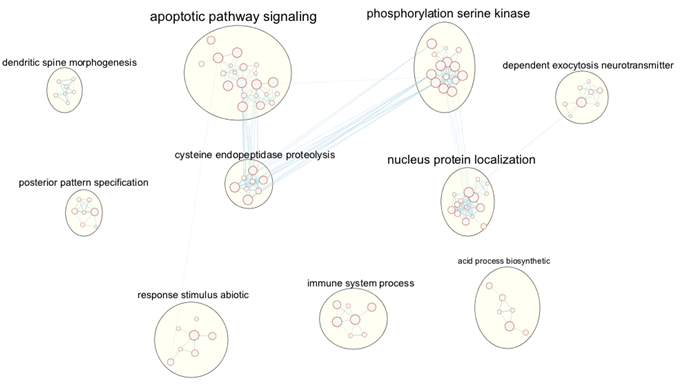


**Figure S29:** Enrichment map of top 10 cluster 4 specific GO terms (biological processes) in AD compared to healthy controls (ROSMAP, gene expression).


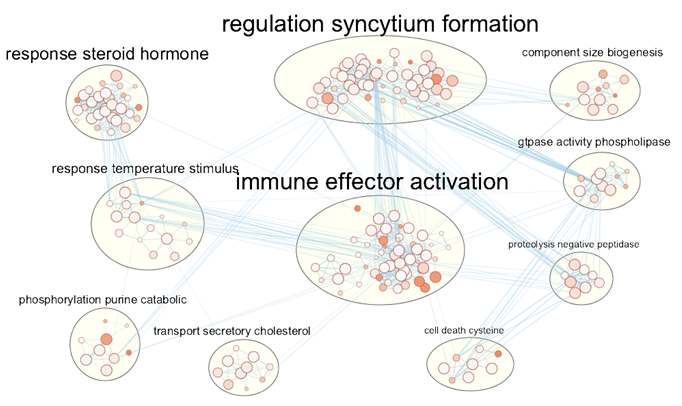


**Figure S30:** Enrichment map of top 10 cluster 2 specific GO terms (biological processes) in PD compared to healthy controls (PPMI, gene expression).


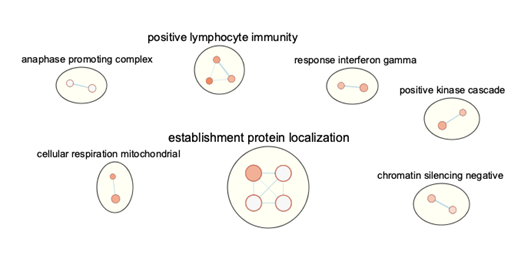


**Figure S31:** Enrichment map of top 10 of cluster 3 specific GO terms (biological processes) in PD compared to healthy controls (PPMI, gene expression).


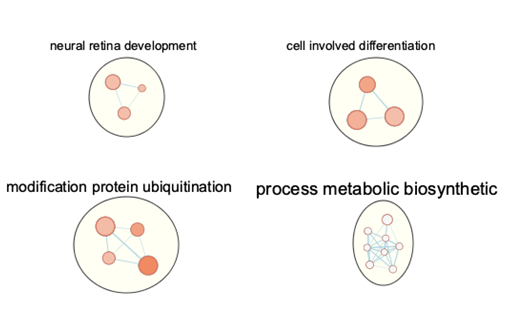


**Figure S32:** Enrichment map of top 10 of cluster 4 specific GO terms (biological processes) in PD compared to healthy controls (PPMI, gene expression).

## Genome-wide Methylome Analysis

IDAT files were downloaded from the synapse ROSMAP portal (<https://www.synapse.org/#!Synapse:syn3219045> - syn3219045). In total 761 patient samples underwent methylation analysis on the Infinium Human Methylation 450k array. DNA was extracted from the gray matter of 100-mg sections of frozen dorsolateral prefrontal cortex frozen samples and 16 ul of DNA with a concentration of 50ng/ul was used for methylation analysis [(De Jager et al., 2014)](https://www.zotero.org/google-docs/?hhmsF4).

For PD patients DNA methylation has recently been made available by PPMI (<http://www.ppmi-info.org/wp-content/uploads/2018/06/04d_Epigenetics_Singleton.pdf>) using the EPIC chip platform.

Pre-processing of DNA methylation data was done in R using minfi (version 1.24.0) [(Aryee et al., 2014)](https://www.zotero.org/google-docs/?TtB9Tl): First, CpG sites with a probe detection p-value above 0.01 were removed. Next, functional normalization was applied [(Fortin et al., 2014)](https://www.zotero.org/google-docs/?rXJ5lx) and CpGs with SNPs in the site of detection were removed. Reported beta values were logit transformed to M values for further statistical downstream analysis. No samples were excluded through quality control, but a PCA plot did reveal a potential batch effect that correlated with study (ROS or MAP). Accordingly, we decided for a batch correction via ComBat [(Johnson et al., 2007)](https://www.zotero.org/google-docs/?TplEeD). Following on from this we selected those DNA methylation samples, for which matching genotype and clinical data were available. Based on the previously described consensus sNMF of the integrated AETIONOMY AD + PD cohort we could assign all 53 AD cases to one of the four clusters. A complete breakdown of the number of samples within each of our groups of interest can be seen in the table below:

**Table S21**: Number of AD cases (Codx 4 or 5) and cognitively normal controls (Cogdx = 1) with DNA methylation data in ROSMAP.

| **Cluster 1** | **Cluster 2** | **Cluster 3** | **Cluster 4** | **Control** |
| --- | --- | --- | --- | --- |
| 7 | 7 | 14 | 25 | 34 |

Differential methylation between CpG sites was tested using limma [(Smyth, 2004)](https://www.zotero.org/google-docs/?Sgo9KC). A linear model was built utilising all samples, and contrasts were extracted in the same way as for gene expression data. Using the same rationale as for transcriptome data we focused on a gene set analysis. This was done via the MethylGSA robust rank aggregation method (version 1.0.3), because traditional RNA-Seq methods of gene set enrichment analysis have been shown to be biased due to the multiple CpGs mapping to individual genes [(Ren and Kuan, 2018)](https://www.zotero.org/google-docs/?xqr3zY). Table 19 shows the number of significant GO terms and KEGG pathways in pairwise comparisons between clusters. Cluster specific GO terms are shown in Figures 33 - 35. **There were no GO terms solely found enriched in cluster 1 and hence no Figure is shown.** Figure 36 shows significant KEGG pathways from transcriptome and methylome analysis.

For PPMI results of the same type of analysis (Illumina EPIC array) are shown in Tables 22 - 25, Figures 37 - 41.

Detailed analysis results can be downloaded from <https://clus2bio.scai.fraunhofer.de/>.

**Table S22**: Methylome analysis (ROSMAP): number of significant GO terms (biological process) and KEGG pathways (false discovery rate < 5%) in pairwise comparisons between clusters. The diagonal shows the number of significant terms compared to healthy controls.

|  | **Cluster 1** | **Cluster 2** | **Cluster 3** | **Cluster 4** | **vs all other clusters** |
| --- | --- | --- | --- | --- | --- |
| **Cluster 1** | GO-BP: 0; KEGG: 1 | GO-BP: 0; KEGG: 1 | GO-BP: 0; KEGG: 0 | GO-BP: 0; KEGG: 0 | GO-BP: 0; KEGG: 0 |
| **Cluster 2** | - | GO-BP: 100 ; KEGG: 3 | GO-BP: 0; KEGG: 0 | GO-BP: 0; KEGG: 0 | GO-BP: 0; KEGG: 3 |
| **Cluster 3** | - | - | GO-BP: 186; KEGG: 0 | GO-BP: 0; KEGG: 0 | GO-BP: 0; KEGG: 0 |
| **Cluster 4** | - | - | - | GO-BP: 356; KEGG: 6 | GO-BP: 0 ; KEGG: 0 |

**Table S23**: Number of PD cases and healthy controls with DNA methylation data in PPMI.

| **Cluster 1** | **Cluster 2** | **Cluster 3** | **Cluster 4** | **Control** |
| --- | --- | --- | --- | --- |
| 19 | 125 | 27 | 106 | 112 |

**Table S24**: Methylome analysis (PPMI): number of significant GO terms (biological process) and KEGG pathways (false discovery rate < 5%) in pairwise comparisons between clusters. The diagonal shows the number of significant terms compared to healthy controls.

|  | **Cluster 1** | **Cluster 2** | **Cluster 3** | **Cluster 4** | **vs all other clusters** |
| --- | --- | --- | --- | --- | --- |
| **Cluster 1** | GO-BP: 320; KEGG: 19 | GO-BP: 570; KEGG: 20 | GO-BP: 0; KEGG: 0 | GO-BP: 0; KEGG: 0 | GO-BP: 189; KEGG: 2 |
| **Cluster 2** | - | GO-BP: 841 ; KEGG: 31 | GO-BP: 171; KEGG: 9 | GO-BP: 33; KEGG: 2 | GO-BP: 698; KEGG: 28 |
| **Cluster 3** | - | - | GO-BP: 153; KEGG: 4 | GO-BP:0 ; KEGG: 2 | GO-BP: 26; KEGG: 9 |
| **Cluster 4** | - | - | - | GO-BP: 567; KEGG: 19 | GO-BP: 44; KEGG: 3 |

**
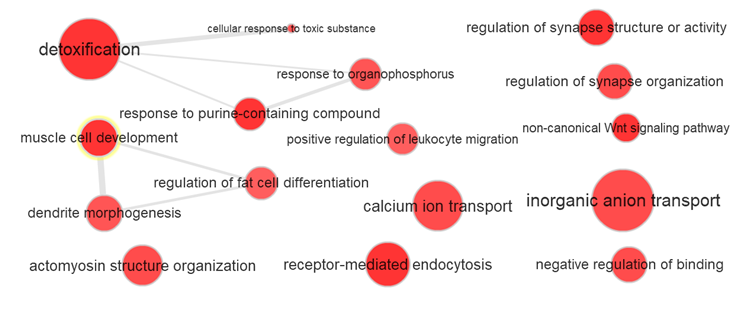
**

**Figure S33:** Enrichment map of cluster 2 specific GO terms (biological processes) in AD compared to healthy controls (ROSMAP, DNA methylation).


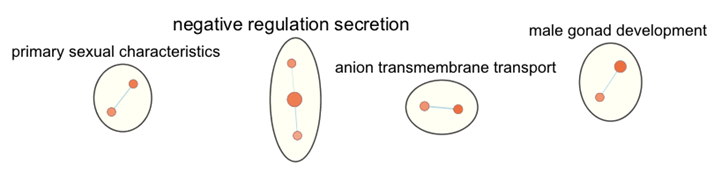


**Figure S34:** Enrichment map of top 10 of cluster 3 specific GO terms (biological processes) in AD compared to healthy controls (ROSMAP, DNA methylation).


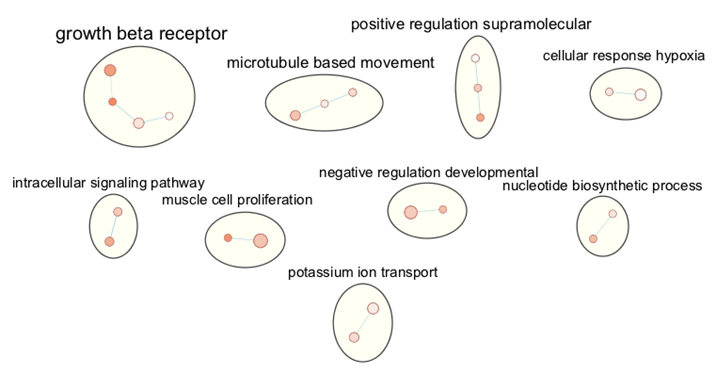


**Figure S35:** Enrichment map of top 10 of cluster 4 specific GO terms (biological processes) in AD compared to healthy controls (ROSMAP, DNA methylation).

**
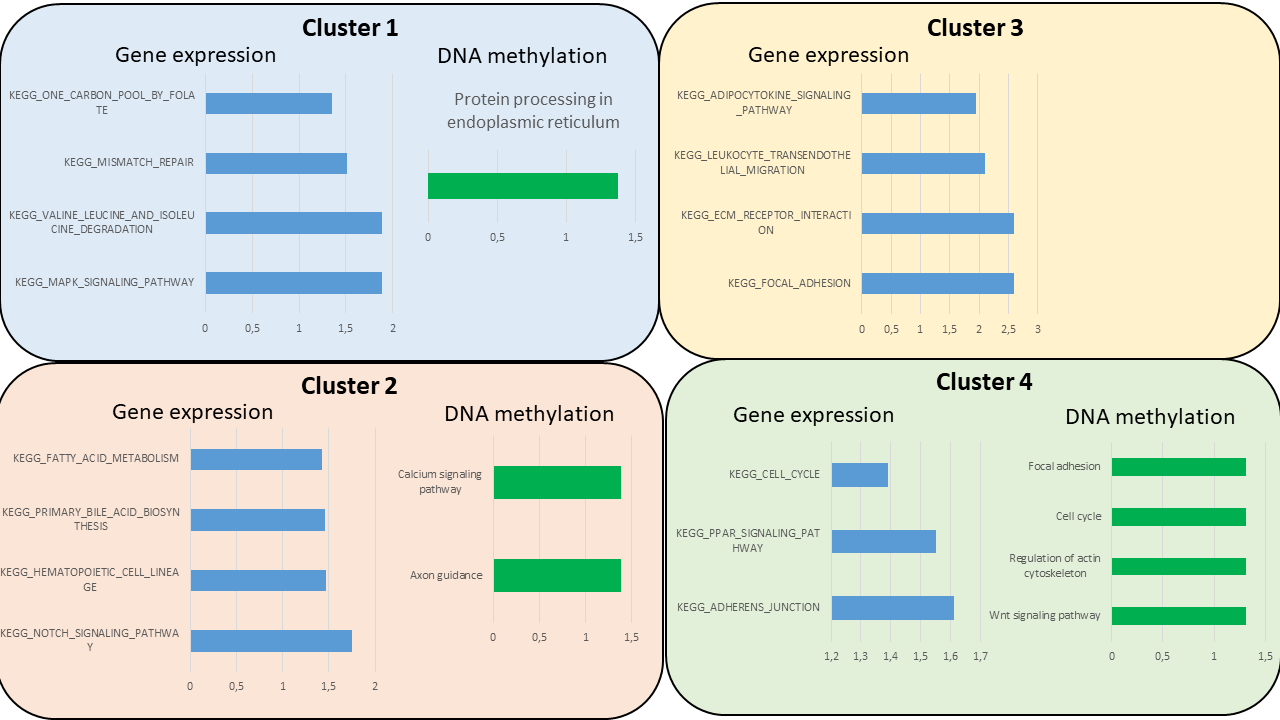
**

**Figure S36:** Overview about cluster specific KEGG pathways in ROSMAP AD data (gene expression and DNA methylation) that are solely found significant in one cluster compared to healthy control. The x-axis always shows -log10(false discovery rate).

**
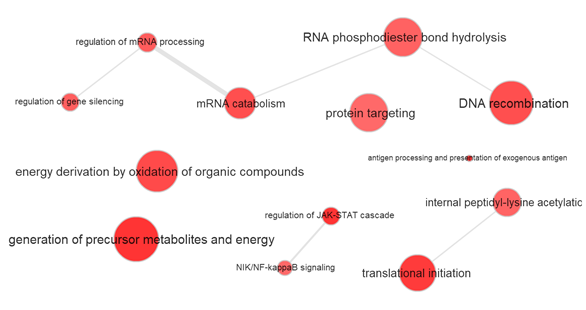
**

**Figure S37:** Enrichment map of cluster 1 specific GO terms (biological processes) in PD compared to healthy controls (PPMI, DNA methylation).


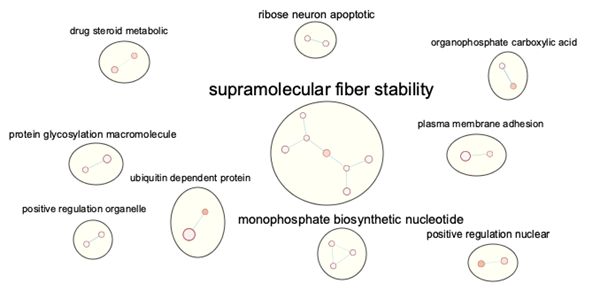


**Figure S38:** Enrichment map of top 10 of cluster 2 specific GO terms (biological processes) in PD compared to healthy controls (PPMI, DNA methylation).


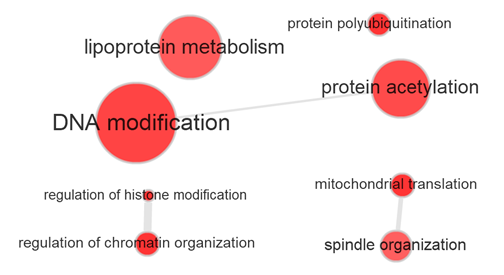


**Figure S39:** Enrichment map of cluster 3 specific GO terms (biological processes) in PD compared to healthy controls (PPMI, DNA methylation).


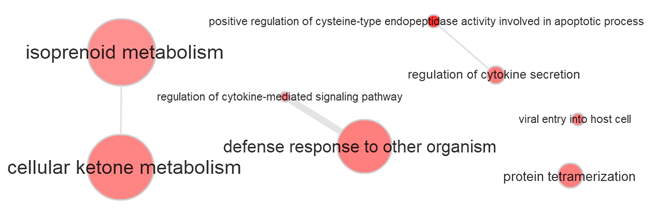


**Figure S40:** Enrichment map of cluster 4 specific GO terms (biological processes) in PD compared to healthy controls (PPMI, DNA methylation).

**
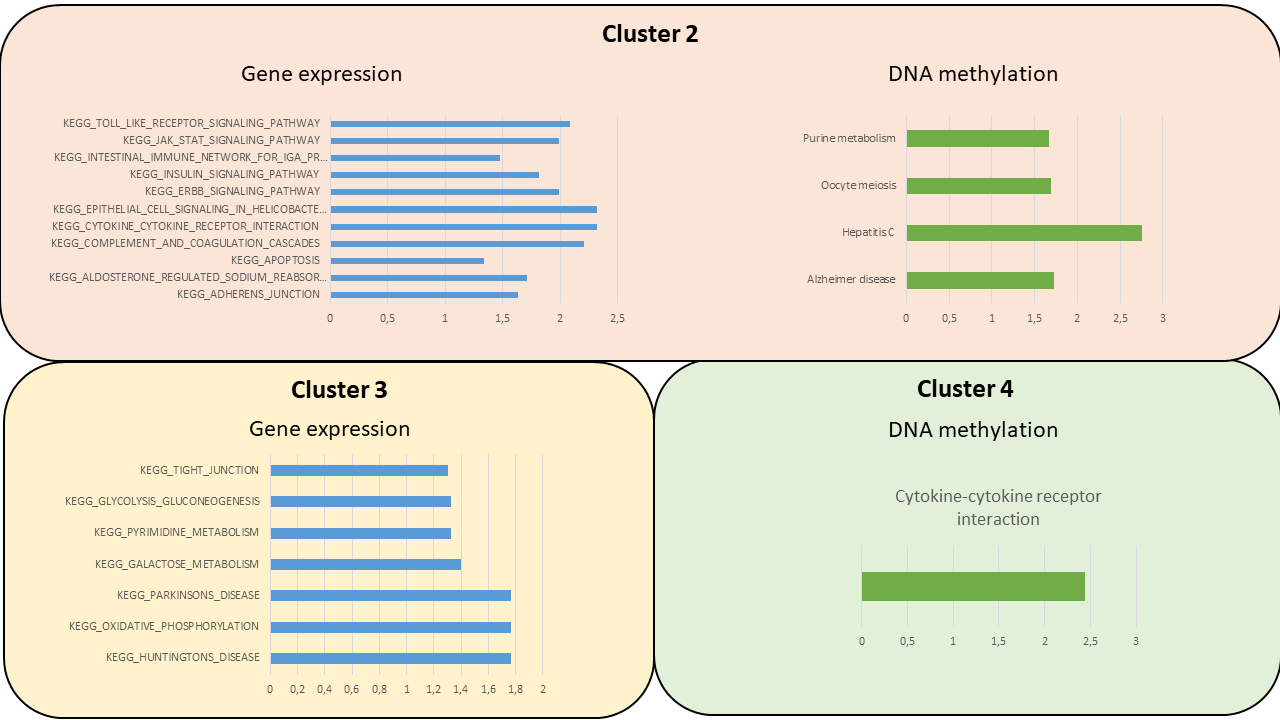
**

**Figure S41:** Overview about cluster specific KEGG pathways in PPMI PD data (gene expression and DNA methylation) that are solely found significant in one cluster compared to healthy control. The x-axis always shows -log10(false discovery rate).

# Calculation of Subcortical Brain Volumes

All available MR scans (T1-weighted scans) from the ADNI database were quantified by an open-source, automated segmentation pipeline at the Erasmus University Medical Center, The Netherlands. The number of slices of the T1w scans varied from 160 to 196 and the in-plane resolution was 256 x 256 on average, yielding an overall voxel-size of 1.2 x 1.0 x 1.0 mm. From the 1715 baseline ADNI scans, the volumes of 193 bilateral subcortical brain regions were calculated using a model- and surface-based automated image segmentation procedure, incorporated in the FreeSurfer Package (v.6.0, http://surfer.nmr.mgh.harvard.edu/). Segmentation in Freesurfer was performed by rigid-body registration and nonlinear normalization of images to a probabilistic brain atlas. In the segmentation process, each voxel of the MRI volumes was labeled automatically as a corresponding brain region based on a cortex parcellation (subdivision) guide. In this case the cortical parcellation method, implemented by [DESTRIEUX et al. (2010)](https://www.zotero.org/google-docs/?92v2U0), was used for brain segmentation. For the subdivision of the human cerebral cortex into gyral-based regions, Destrieux et al. identified subcortical regions in the individual hemispheres. This information was encoded into an atlas that was utilized to automatically label ROIs.

# Potential Implications for Drug Development

## Alzheimer’s Disease


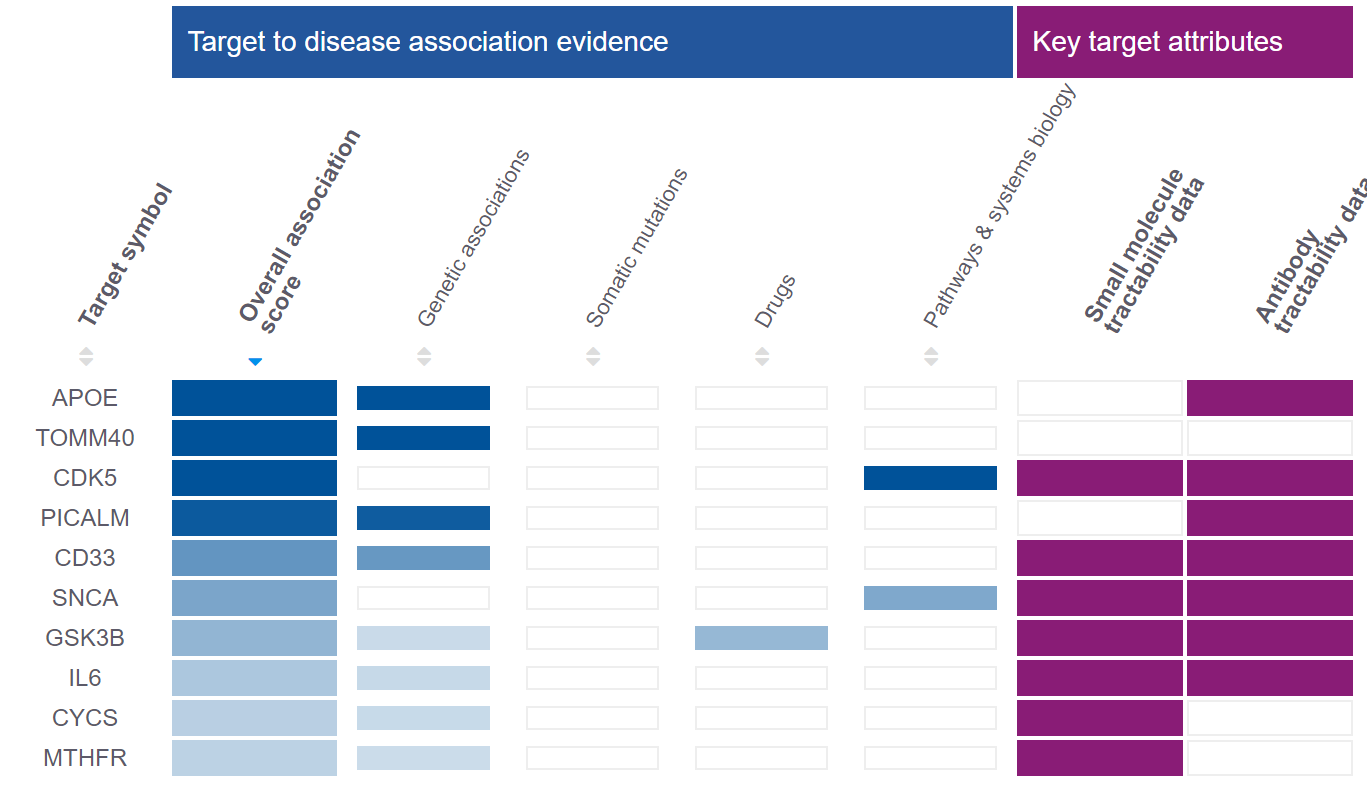


**Figure S42:** Target prioritization view of genes contained in mechanism based clustering according to Open Targets. Darker color indicates a stronger literature based evidence (e.g. due to the existence of ongoing clinical trials).

## Parkinson’s Disease


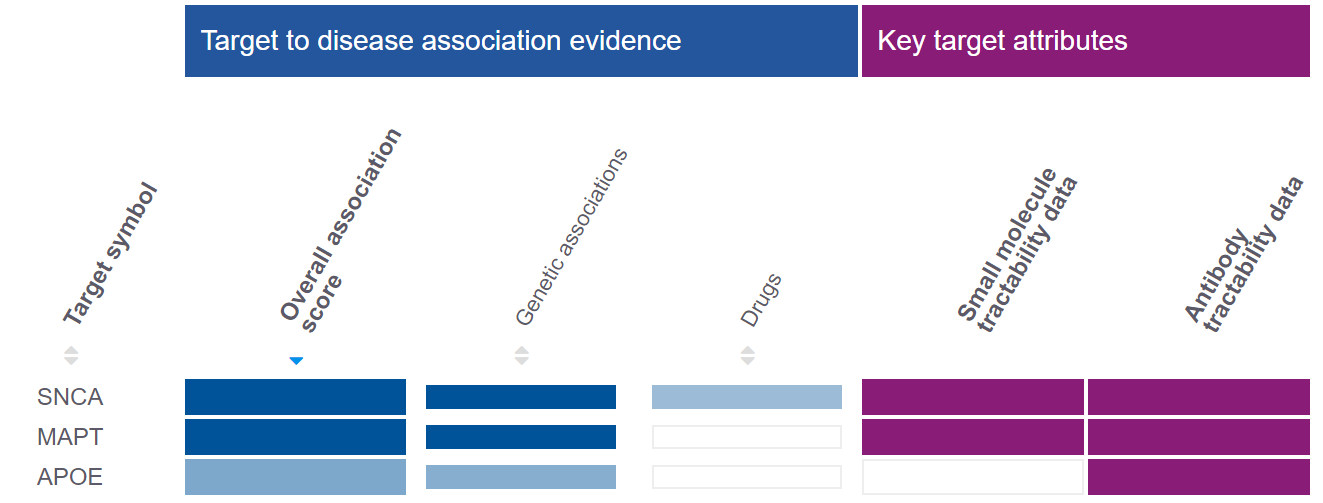


**Figure S43:** Target prioritization view of genes contained in mechanism based clustering according to Open Targets. Darker color indicates a stronger literature based evidence (e.g. due to the existence of ongoing clinical trials).

# Overview about Clinical Study Groups

## AETIONOMY

The members of the AETIONOMY Clinical Consortium include (in alphabetical order of their country affiliations):

Sarah Bujac, UCB Pharma SA, Belgium

Bethan Clarke, UCB Pharma SA, Belgium

Jacqueline Marovac, UCB Pharma SA, Belgium

Phil Scordis, UCB Pharma SA, Belgium

Stephanie Carvalho, Institut du Cerveau et de la Moelle épinière, Paris, France

Jean-Christophe Corvol, Institut du Cerveau et de la Moelle épinière, Paris, France

Bruno Dubois, Institut du Cerveau et de la Moelle épinière, France

Cecile Gaudebout, Institut du Cerveau et de la Moelle épinière, Paris, France

Graziella Mangone, Institut du Cerveau et de la Moelle épinière, Paris, France

Sylvie Forlani, Banque ADN & cellules, Institut du Cerveau et de la Moelle épinière, Paris, France

Ludmila Jornea, Banque ADN & cellules, Institut du Cerveau et de la Moelle épinière, Paris, France

Philippe Martin-Hardy, Banque ADN & cellules, Institut du Cerveau et de la Moelle épinière, Paris, France

Yassaman Ghassab, Banque ADN & cellules, Institut du Cerveau et de la Moelle épinière, Paris, France

Eloi Magnin, CHU Besançon, France

Alexandra Foubert-Samier, IMNc, Hôpital Pellegrin, CHU Bordeaux, France

Brice Laurens, IMNc, Hôpital Pellegrin, CHU Bordeaux, France

Wassilios Meissner, Hopital Pellegrin Bordeaux, France

Umberto Spampinato, Hôpital Pellegrin, CHU Bordeaux, France

Sylvain Vergnet, IMNc, Hôpital Pellegrin, CHU Bordeaux, France

Olivier Rascol, CHU Toulouse, France

Claire Thalamas, CHU Toulouse, France

Monique Galitzky, CHU Toulouse, France

Fabienne Calvas, CHU Toulouse, France

Fabienne Ory-Magne, CHU Toulouse, France

Christine Brefel-Courbon, CHU Toulouse, France

Michael Heneka, Universitaetsklinikum Bonn, Germany

Pawel Tacik, Universitaetsklinikum Bonn, Germany

Ullrich Wüllner, Universitaetsklinikum Bonn, Germany

Martin Hofmann-Apitius, Fraunhofer Institute for Algorithms and Scientific Computing SCAI, Germany

José Luis Molinuevo, Barcelonabeta Brain Research Center, Spain

Mircea Balasa, Consorci Institut d’Investigacions Biomediques August Pi i Sunyer, Spain

Beatriz Bosch, Consorci Institut d'Investigacions Biomediques August Pi i Sunyer, Spain

Spain Raquel Sánchez-Valle, Consorci Institut d'Investigacions Biomediques August Pi i Sunyer, Spain

Ioanna Markaki, Karolinska Institutet, Stockholm, Sweden

Per Svenningsson, Karolinska Institutet, Stockholm, Sweden

Panagiota Tsitsi, Karolinska Institutet, Stockholm, Sweden

## DIGPD

Steering committee: Jean-Christophe Corvol, MD, PhD (Pitié-Salpêtrière Hospital, Paris, principal investigator of DIGPD), Alexis Elbaz, MD, PhD (CESP, Villejuif, member of the steering committee), Marie Vidailhet, MD (Pitié-Salpêtrière Hospital, Paris, member of the steering committee), Alexis Brice, MD (Pitié-Salpêtrière Hospital, Paris, member of the steering committee and PI for genetic analysis) ;

Statistical analyses: Alexis Elbaz, MD, PhD (CESP, Villejuif, PI for statistical analyses), Fanny Artaud, PhD (CESP, Villejuif, statistician);

Principal investigators for sites (alphabetical order): Frédéric Bourdain, MD (CH Foch, Suresnes, PI for site), Jean-Philippe Brandel, MD (Fondation Rothschild, Paris, PI for site), Jean-Christophe Corvol, MD, PhD (Pitié-Salpêtrière Hospital, Paris, PI for site), Pascal Derkinderen, MD, PhD (CHU Nantes, PI for site), Franck Durif, MD (CHU Clermont-Ferrand, PI for site), Richard Levy, MD, PhD (CHU Saint-Antoine, Paris, PI for site), Fernando Pico, MD (CH Versailles, PI for site), Olivier Rascol, MD (CHU Toulouse, PI for site);

Co-investigators (alphabtical order): Anne-Marie Bonnet, MD (Pitié-Salpêtrière Hospital, Paris, site investigator), Cecilia Bonnet, MD, PhD (Pitié-Salpêtrière Hospital, Paris, site investigator), Christine Brefel-Courbon, MD (CHU Toulouse, site investigator), Florence Cormier-Dequaire, MD (Pitié-Salpêtrière Hospital, Paris, site investigator), Bertrand Degos, MD, PhD (Pitié-Salpêtrière Hospital, site investigator), Bérangère Debilly, MD (CHU Clermont-Ferrand, site investigator), Alexis Elbaz, MD, PhD (Pitié-Salpêtrière Hospital, Paris, site investigator), Monique Galitsky (CHU de Toulouse, site investigator), David Grabli, MD, PhD (Pitié-Salpêtrière Hospital, Paris, site investigator), Andreas Hartmann, MD, PhD (Pitié-Salpêtrière Hospital, Paris, site investigator), Stephan Klebe, MD (Pitié-Salpêtrière Hospital, Paris, site investigator), Julia Kraemmer, MD (Pitié-Salpêtrière Hospital, site investigator), Lucette Lacomblez, MD (Pitié-Salpêtrière Hospital, Paris, site investigator), Sara Leder, MD (Pitié-Salpêtrière Hospital, Paris, site investigator), Graziella Mangone, MD, PhD (Pitié-Salpêtrière Hospital, Paris, site investigator), Louise-Laure Mariani, MD (Pitié-Salpêtrière Hospital, Paris, site investigator), Ana-Raquel Marques, MD (CHU Clermont Ferrand, site investigator), Valérie Mesnage, MD (CHU Saint Antoine, Paris, site investigator), Julia Muellner, MD (Pitié-Salpêtrière Hospital, Paris, site investigator), Fabienne Ory-Magne, MD (CHU Toulouse, site investigator), Violaine Planté-Bordeneuve, MD (Henri Mondor Hospital, Créteil, site investigator), Emmanuel Roze, MD, PhD (Pitié-Salpêtrière Hospital, Paris, site investigator), Melissa Tir, MD (CH Versailles, site investigator), Marie Vidailhet, MD (Pitié-Salpêtrière Hospital, Paris, site investigator), Hana You, MD (Pitié-Salpêtrière Hospital, Paris, site investigator);

Neuropsychologists: Eve Benchetrit, MS (Pitié-Salpêtrière Hospital, Paris, neuropsychologist), Julie Socha, MS (Pitié-Salpêtrière Hospital, Paris, neuropsychologist), Fanny Pineau, MS (Pitié-Salpêtrière Hospital, Paris, neuropsychologist), Tiphaine Vidal, MS (CHU Clermont-Ferrand, neuropsychologist), Elsa Pomies (CHU de Toulouse, neuropsychologist), Virginie Bayet (CHU de Toulouse, neuropsychologist);

Genetic core: Alexis Brice (Pitié-Salpêtrière Hospital, Paris, PI for genetic studies), Suzanne Lesage, PhD (INSERM, ICM, Paris, genetic analyses), Khadija Tahiri, PhD (INSERM, ICM, Paris, lab technician) Hélène Bertrand, MS (INSERM, ICM, Paris, lab technician), Graziella Mangone, MD, PhD (Pitié-Salpêtrière Hospital, Paris, genetic analyses);

Sponsor activities and clinical research assistants: Alain Mallet, PhD (Pitié-Salpêtrière Hospital, Paris, sponsor representative), Coralie Villeret (Hôpital Saint Louis, Paris, Project manager), Merry Mazmanian (Pitié-Salpêtrière Hospital, Paris, project manager), Hakima Manseur (Pitié-Salpêtrière Hospital, Paris, clinical research assistant), Mostafa Hajji (Pitié-Salpêtrière Hospital, Paris, data manager), Benjamin Le Toullec, MS (Pitié-Salpêtrière Hospital, Paris, clinical research assistant), Vanessa Brochard, PhD (Pitié-Salpêtrière Hospital, Paris, project manager), Monica Roy, MS (CHU de Nantes, clinical researh assistant), Isabelle Rieu, PhD (CHU Clermont-Ferrand, clinical research assistant), Stéphane Bernard (CHU Clermont-Ferrand, clinical research assistant), Antoine Faurie-Grepon (CHU de Toulouse, clnical research assistant).

## ICEBERG

Steering committee: Marie Vidailhet, MD, PhD, (Pitié-Salpêtrière Hospital, Paris, principal investigator of ICEBERG), Jean-Christophe Corvol, MD, PhD (Pitié-Salpêtrière Hospital, Paris, scientific lead), Isabelle Arnulf, MD, PhD (Pitié-Salpêtrière Hospital, Paris, member of the steering committee), Stéphane Lehericy, MD, PhD (Pitié-Salpêtrière Hospital, Paris, member of the steering committee);

Clinical data : Marie Vidailhet, MD, PhD, (Pitié-Salpêtrière Hospital, Paris, coordination), Graziella Mangone, MD, PhD (Pitié-Salpêtrière Hospital, Paris, co-coordination), Jean-Christophe Corvol, MD, PhD (Pitié-Salpêtrière Hospital, Paris), Isabelle Arnulf, MD, PhD (Pitié-Salpêtrière Hospital, Paris), Jonas Ihle, MD (Pitié-Salpêtrière Hospital, Paris), Caroline Weill, MD, (Pitié-Salpêtrière Hospital, Paris), David Grabli, MD, PhD (Pitié-Salpêtrière Hospital, Paris); Florence Cormier-Dequaire, MD (Pitié-Salpêtrière Hospital, Paris); Louise Laure Mariani, MD, PhD (Pitié-Salpêtrière Hospital, Paris), Bertrand Degos, MD, PhD (Avicenne Hospital, Bobigny);

Neuropsychological data : Richard Levy, MD (Pitié-Salpêtrière Hospital, Paris, coordination), Fanny Pineau, MS (Pitié-Salpêtrière Hospital, Paris, neuropsychologist), Julie Socha, MS (Pitié-Salpêtrière Hospital, Paris, neuropsychologist), Eve Benchetrit, MS (La Timone Hospital, Marseille, neuropsychologist), Virginie Czernecki, MS (Pitié-Salpêtrière Hospital, Paris, neuropsychologist);

Eye movement : Sophie Rivaud-Pechoux, PhD (ICM, Paris, coordination); Elodie Hainque, MD, PhD (Pitié-Salpêtrière Hospital, Paris);

Sleep assessment: Isabelle Arnulf, MD, PhD (Pitié-Salpêtrière Hospital, Paris, coordination), Smaranda Leu Semenescu, MD (Pitié-Salpêtrière Hospital, Paris), Pauline Dodet, MD (Pitié-Salpêtrière Hospital, Paris);

Genetic data: Jean-Christophe Corvol, MD, PhD (Pitié-Salpêtrière Hospital, Paris, coordination), Graziella Mangone, MD, PhD (Pitié-Salpêtrière Hospital, Paris, co-coordination), Samir Bekadar, MS (Pitié-Salpêtrière Hospital, Paris, biostatistician), Alexis Brice, MD (ICM, Pitié-Salpêtrière Hospital, Paris), Suzanne Lesage, PhD (INSERM, ICM, Paris, genetic analyses);

Metabolomics: Fanny Mochel, MD, PhD (Pitié-Salpêtrière Hospital, Paris, coordination), Farid Ichou, PhD (ICAN, Pitié-Salpêtrière Hospital, Paris), Vincent Perlbarg, PhD, Pierre and Marie Curie University), Benoit Colsch, PhD (CEA, Saclay), Arthur Tenenhaus, PhD (Supelec, Gif-sur-Yvette, data integration);

Brain MRI data : Stéphane Lehericy, MD, PhD (Pitié-Salpêtrière Hospital, Paris, coordination), Rahul Gaurav, MS, (Pitié-Salpêtrière Hospital, Paris, data analysis), Nadya Pyatigorskaya, MD, PhD, (Pitié-Salpêtrière Hospital, Paris, data analysis); Lydia Yahia-Cherif, PhD (ICM, Paris, Biostatistics), Romain Valabregue, PhD (ICM, Paris, data analysis), Cécile Galléa, PhD (ICM, Paris);

Datscan imaging data: Marie-Odile Habert, MCU-PH (Pitié-Salpêtrière Hospital, Paris, coordination);

Voice recording: Dijana Petrovska, PhD (Telecom Sud Paris, Evry, coordination), Laetitia Jeancolas, MS (Telecom Sud Paris, Evry);

Study management: Vanessa Brochard (Pitié-Salpêtrière Hospital, Paris, coordination), Alizé Chalançon (Pitié-Salpêtrière Hospital, Paris, clinical research assistant), Carole Dongmo-Kenfack (Pitié-Salpêtrière Hospital, Paris, clinical research assistant);

# References

1. Bradshaw, E. M., *et al.* (2013). CD33 Alzheimer's disease locus: altered monocyte function and amyloid biology. *Nature neuroscience*, *16*(7), 848.
2. Kim, J., Basak, J. M., & Holtzman, D. M. (2009). The role of apolipoprotein E in Alzheimer's disease. *Neuron*, *63*(3), 287-303.
3. Kim, W. S., Kågedal, K., & Halliday, G. M. (2014). Alpha-synuclein biology in Lewy body diseases. *Alzheimer's research & therapy*, *6*(5), 73.
4. Letoha, T., *et al.* (2019). Contribution of syndecans to cellular internalization and fibrillation of amyloid-β (1–42). *Scientific reports*, *9*(1), 1393.
5. Licking, N., *et al.* (2017). Homocysteine and cognitive function in Parkinson's disease. *Parkinsonism & related disorders*, *44*, 1-5.
6. Lyall, D. M., *et al.* (2014). Alzheimer's disease susceptibility genes APOE and TOMM40, and brain white matter integrity in the Lothian Birth Cohort 1936. *Neurobiology of aging*, *35*(6), 1513-e25.
7. Morris, M. S. (2003). Homocysteine and Alzheimer's disease. *The Lancet Neurology*, *2*(7), 425-428.
8. Ran, C., Westerlund, M., Anvret, A., Willows, T., Sydow, O., Galter, D., & Belin, A. C. (2011). Genetic studies of the protein kinase AKT1 in Parkinson's disease. *Neuroscience letters*, *501*(1), 41-44.
9. Rickle, A., Bogdanovic, N., Volkman, I., Winblad, B., Ravid, R., & Cowburn, R. F. (2004). Akt activity in Alzheimer's disease and other neurodegenerative disorders. *Neuroreport*, *15*(6), 955-959.
10. Roman, G. C. (2015). MTHFR Gene Mutations: A Potential Marker of Late-Onset Alzheimer’s Disease?. *Journal of Alzheimer's Disease*, *47*(2), 323-327.
11. Ohta, E., Kawakami, F., Kubo, M., & Obata, F. (2011). LRRK2 directly phosphorylates Akt1 as a possible physiological substrate: Impairment of the kinase activity by Parkinson's disease‐associated mutations. *FEBS letters*, *585*(14), 2165-2170.
12. Xu, W., *et al.* (2016). The impact of PICALM genetic variations on reserve capacity of posterior cingulate in AD continuum. *Scientific reports*, *6*, 24480.
13. Leek JT, Storey JD (2007) Capturing Heterogeneity in Gene Expression Studies by Surrogate Variable Analysis. PLoS Genet 3(9): e161. <https://doi.org/10.1371/journal.pgen.0030161>
14. Aaron R. Quinlan, Ira M. Hall (2010), BEDTools: a flexible suite of utilities for comparing genomic features, *Bioinformatics*, Volume 26, Issue 6, 15 March 2010, Pages 841–842,<https://doi.org/10.1093/bioinformatics/btq033>
15. [Aryee, M.J., Jaffe, A.E., Corrada-Bravo, H., Ladd-Acosta, C., Feinberg, A.P., Hansen, K.D., and Irizarry, R.A. (2014). Minfi: a flexible and comprehensive Bioconductor package for the analysis of Infinium DNA methylation microarrays. Bioinformatics *30*, 1363–1369.](https://www.zotero.org/google-docs/?LWhQiX)
16. [Benachir, D., Hosseini, S., Deville, Y., Karoui, M.S., and Hameurlain, A. (2013). Modified independent component analysis for initializing non-negative matrix factorization: An approach to hyperspectral image unmixing. In 2013 IEEE 11th International Workshop of Electronics, Control, Measurement, Signals and Their Application to Mechatronics, pp. 1–6.](https://www.zotero.org/google-docs/?LWhQiX)
17. [Benjamini, Y., and Hochberg, Y. (1995). Controlling the False Discovery Rate: A Practical and Powerful Approach to Multiple Testing. J. Royal Statist. Soc., Series B *57*, 289–300.](https://www.zotero.org/google-docs/?LWhQiX)
18. [Carithers, L.J., Ardlie, K., Barcus, M., Branton, P.A., Britton, A., Buia, S.A., Compton, C.C., DeLuca, D.S., Peter-Demchok, J., Gelfand, E.T., et al. (2015). A Novel Approach to High-Quality Postmortem Tissue Procurement: The GTEx Project. Biopreservation and Biobanking *13*, 311–319.](https://www.zotero.org/google-docs/?LWhQiX)
19. [Das, S., Forer, L., Schönherr, S., Sidore, C., Locke, A.E., Kwong, A., Vrieze, S.I., Chew, E.Y., Levy, S., McGue, M., et al. (2016). Next-generation genotype imputation service and methods. Nat Genet *48*, 1284–1287.](https://www.zotero.org/google-docs/?LWhQiX)
20. [De Jager, P.L., Srivastava, G., Lunnon, K., Burgess, J., Schalkwyk, L.C., Yu, L., Eaton, M.L., Keenan, B.T., Ernst, J., McCabe, C., et al. (2014). Alzheimer’s disease: early alterations in brain DNA methylation at ANK1, BIN1, RHBDF2 and other loci. Nat. Neurosci. *17*, 1156–1163.](https://www.zotero.org/google-docs/?LWhQiX)
21. [DESTRIEUX, C., FISCHL, B., DALE, A., and HALGREN, E. (2010). Automatic parcellation of human cortical gyri and sulci using standard anatomical nomenclature. Neuroimage *53*, 1–15.](https://www.zotero.org/google-docs/?LWhQiX)
22. [Domingo-Fernandez, D., Kodamullil, A.T., Iyappan, A., Naz, M., Emon, M.A., Raschka, T., Karki, R., Springstubbe, S., Ebeling, C., and Hofmann-Apitius, M. (2017). Multimodal Mechanistic Signatures for Neurodegenerative Diseases (NeuroMMSig): a web server for mechanism enrichment. Bioinformatics.](https://www.zotero.org/google-docs/?LWhQiX)
23. [Fortin, J.-P., Labbe, A., Lemire, M., Zanke, B.W., Hudson, T.J., Fertig, E.J., Greenwood, C.M., and Hansen, K.D. (2014). Functional normalization of 450k methylation array data improves replication in large cancer studies. Genome Biology *15*, 503.](https://www.zotero.org/google-docs/?LWhQiX)
24. [Gaujoux, R., and Seoighe, C. (2010). A flexible R package for nonnegative matrix factorization. BMC Bioinformatics *11*, 367.](https://www.zotero.org/google-docs/?LWhQiX)
25. [Gedeon, T.D. (1997). Data mining of inputs: analysing magnitude and functional measures. Int J Neural Syst *8*, 209–218.](https://www.zotero.org/google-docs/?LWhQiX)
26. [Hand, D.J., and Till, R.J. (2001). A Simple Generalisation of the Area Under the ROC Curve for Multiple Class Classification Problems. Machine Learning *45*, 171–186.](https://www.zotero.org/google-docs/?LWhQiX)
27. [Johnson, W.E., Li, C., and Rabinovic, A. (2007). Adjusting batch effects in microarray expression data using empirical Bayes methods. Biostat *8*, 118–127.](https://www.zotero.org/google-docs/?LWhQiX)
28. [Kanehisa, M., Araki, M., Goto, S., Hattori, M., Hirakawa, M., Itoh, M., Katayama, T., Kawashima, S., Okuda, S., Tokimatsu, T., et al. (2008). KEGG for linking genomes to life and the environment. Nucleic Acids Res. *36*, 480–484.](https://www.zotero.org/google-docs/?LWhQiX)
29. [Kapp, A.V., and Tibshirani, R. (2007). Are clusters found in one dataset present in another dataset? Biostatistics *8*, 9–31.](https://www.zotero.org/google-docs/?LWhQiX)
30. [Kim, H., and Park, H. (2007). Sparse non-negative matrix factorizations via alternating non-negativity-constrained least squares for microarray data analysis. Bioinformatics *23*, 1495–1502.](https://www.zotero.org/google-docs/?LWhQiX)
31. [Luo, W., Friedman, M.S., Shedden, K., Hankenson, K.D., and Woolf, P.J. (2009). GAGE: generally applicable gene set enrichment for pathway analysis. BMC Bioinformatics *10*, 161.](https://www.zotero.org/google-docs/?LWhQiX)
32. [McCarthy, D.J., Chen, Y., and Smyth, G.K. (2012). Differential expression analysis of multifactor RNA-Seq experiments with respect to biological variation. Nucleic Acids Res. *40*, 4288–4297.](https://www.zotero.org/google-docs/?LWhQiX)
33. [Merico, D., Isserlin, R., Stueker, O., Emili, A., and Bader, G.D. (2010). Enrichment Map: A Network-Based Method for Gene-Set Enrichment Visualization and Interpretation. PLOS ONE *5*, e13984.](https://www.zotero.org/google-docs/?LWhQiX)
34. [Mostafavi, S., Gaiteri, C., Sullivan, S.E., White, C.C., Tasaki, S., Xu, J., Taga, M., Klein, H.-U., Patrick, E., Komashko, V., et al. (2018). A molecular network of the aging human brain provides insights into the pathology and cognitive decline of Alzheimer’s disease. Nature Neuroscience *21*, 811.](https://www.zotero.org/google-docs/?LWhQiX)
35. [Ren, X., and Kuan, P.F. (2018). methylGSA: a Bioconductor package and Shiny app for DNA methylation data length bias adjustment in gene set testing. Bioinformatics.](https://www.zotero.org/google-docs/?LWhQiX)
36. [Smyth, G. (2004). Linear models and empirical Bayes methods for assessing differential expression in microarray experiments. Statistical Applications in Genetics and Molecular Biology *3*.](https://www.zotero.org/google-docs/?LWhQiX)
37. [Srivastava, N., Hinton, G., Krizhevsky, A., Sutskever, I., and Salakhutdinov, R. (2014). Dropout: A Simple Way to Prevent Neural Networks from Overfitting. Journal of Machine Learning Research *15*, 1929–1958.](https://www.zotero.org/google-docs/?LWhQiX)
38. [Subramanian, A., Tamayo, P., Mootha, V.K., Mukherjee, S., Ebert, B.L., Gillette, M.A., Paulovich, A., Pomeroy, S.L., Golub, T.R., Lander, E.S., et al. (2005). Gene set enrichment analysis: a knowledge-based approach for interpreting genome-wide expression profiles. Proc Natl Acad Sci U S A *102*, 15545–15550.](https://www.zotero.org/google-docs/?LWhQiX)
39. [the Haplotype Reference Consortium, McCarthy, S., Das, S., Kretzschmar, W., Delaneau, O., Wood, A.R., Teumer, A., Kang, H.M., Fuchsberger, C., Danecek, P., et al. (2016). A reference panel of 64,976 haplotypes for genotype imputation. Nature Genetics *48*, 1279–1283.](https://www.zotero.org/google-docs/?LWhQiX)
